# Viologen-based solution-processable ionic porous polymers for electrochromic applications†

**DOI:** 10.1039/d4sc01408a

**Published:** 2024-04-23

**Authors:** Hongya Miao, Ling Chen, Fangfang Xing, Huijie Li, Thomas Baumgartner, Xiaoming He

**Affiliations:** a Key Laboratory of Applied Surface and Colloid Chemistry (Ministry of Education), School of Chemistry and Chemical Engineering, Shaanxi Normal University Xi'an 710119 P. R. China xmhe@snnu.edu.cn; b Department of Chemistry, York University 4700 Keele Street Toronto Ontario M3J 1P3 Canada tbaumgar@yorku.ca

## Abstract

Electrochromic porous thin films are promising for applications in smart windows and energy-efficient optical displays. However, their generally poor processing ability and excessive processing times remain grand challenges. Herein, we report the design and convenient synthesis of core-altered *N*-arylated viologens with aldehyde groups (πV-CHO) as new building blocks to prepare soluble, viologen-embedded ionic porous polymers. We also demonstrate that these polymers can be easily solution-processed by drop-coating to fabricate high-quality electrochromic films with tunable optoelectronic properties in a cost-effective fashion. The prepared films exhibit excellent electrochromic performance, including a low driving voltage (1.2–1.4 V), fast switching times (0.8–1.7 s), great coloration efficiency (73–268 cm^2^ C^−1^), remarkably high optical contrast up to 95.6%, long cycling stability, and tunable oxidation and reduction colors. This work sheds important light on a new molecular engineering approach to produce redox-active polymers with combined properties of intrinsic porosity, reversible and tunable redox activity, and solution processability. This provides the materials with an inherently broad utility in a variety of electrochemical devices for energy storage, sensors, and electronic applications.

## Abstract 2

A series of *N*-arylated viologens with terminal aldehyde groups has been synthesized. The new building blocks were successfully used to construct solution-processible, redox-active ionic porous polymers, for high-performance electrochromic films.

## Graphical abstract

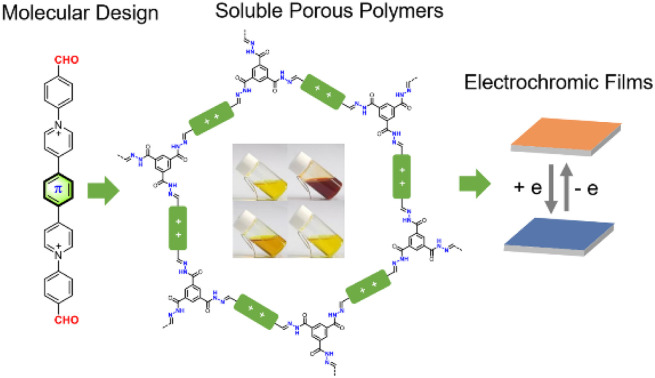

## Introduction

Electrochromic (EC) materials that can reversibly change colors by applying an electric potential, have attracted significant research interest in various areas, such as smart windows, rear-view mirrors, electrochromic e-skins, and information storage.^1–3^ Transparent EC thin films with tunable colored and bleached states are a key requirement for achieving critical device performance. Most state-of-the-art electrochromic materials are based on inorganic transition metal oxides (*e.g.* WO_3_).^4^ While these inorganic materials have been demonstrated to operate at low voltages with efficient electrical energy consumption and show high optical contrast, their limited color variation and the high cost of film fabrication, however, pose great challenges to extend their application scope. Therefore, exploration of new materials for cost-effective electrochromic film fabrication continues to be at the forefront of EC research.

Organic materials have gained considerable attention for EC applications owing to their structural versatility and optoelectronic tunability, as well as their outstanding processability. To date, various redox-active organic materials, such as small-molecule viologens and linear conjugated polymers with tunable optoelectronic properties have successfully been developed.^5,6^ However, their practical application is hampered by long switching speeds (>3 s) and relatively poor stability, due to sluggish ion- and electron-transport during operation. Recent developments toward high-performance EC thin films have led to porous polymers with improved switching time and coloration, when compared with conventional, dense electrochromic membranes.^7–12^ The presence of nanopores in the films provides effective pathways for efficient ion diffusion and mass transport, making the redox sites more accessible to metal ions and analytes. For instance, Dincă *et al.* have developed films based on a mesoporous electrochromic metal–organic framework (MOF) that can reversibly switch between transparent and colored states.^11^ Bein and coworkers reported a fully organic, porous covalent–organic framework (COF) film with high coloration efficiency and short switching time.^9^ However, the reported procedures for fabricating such porous thin films typically rely on solvothermal methods and liquid–liquid interface polymerization.^7–12^ These possessing steps add increased complexity and cost to the fabrication process, and cause difficulties with regard to reproduction or scale-up. Hence, development of solution-processible, redox-active porous polymers is highly desirable for ease and large-scale fabrication of electrochromic films.

Towards this goal, the rational design of redox-active building blocks and development of efficient reaction conditions are very important. So far, most COFs and MOFs are constructed from neutral building blocks and the resulting materials are insoluble in most solvents due to the strong interlayer π–π interaction. This poses a significant challenge for the design of suitable materials. Alleviating the strong interlayer interactions has been demonstrated as an effective strategy to improve the stability of two-dimensional porous polymers in solution by electrostatic charge repulsion. For instance, Jiang and coworkers report a versatile synthesis of highly soluble, charged COF nanosheets from a single solution by using dynamic covalent bonds.^13^ The obtained COF solutions enable facile casting of thin films for proton exchange. Such emerging ionic porous polymers (IPPs) have attracted increasing attention and show promising utility in ion exchange membranes, energy-storage, and -conversion devices.^14^ However, the limited availability of suitable redox-active ionic building blocks restricts their broad exploration as functional electrochromic materials.

Viologens have attracted our attention, due to their cationic skeletons and promising electrochromic properties.^5,6^ This scaffold can typically undergo two reversible reduction processes at low potentials that are accompanied by intense color changes. At present, most studies mainly concentrate on *N*-substitution and core extension strategies for the synthesis of small-molecule based viologen derivatives, to tune their electrochromic features.^15–20^ One obvious weakness of molecular viologen-based EC devices is the necessity of a solution electrolyte, that potentially causes problematic leakage during long-term cycling. We anticipated that functionalization of viologens with suitable reactive groups would enable the construction of solution-processible IPPs for electrochromic films with improved performance at low cost.

**Scheme 1:**
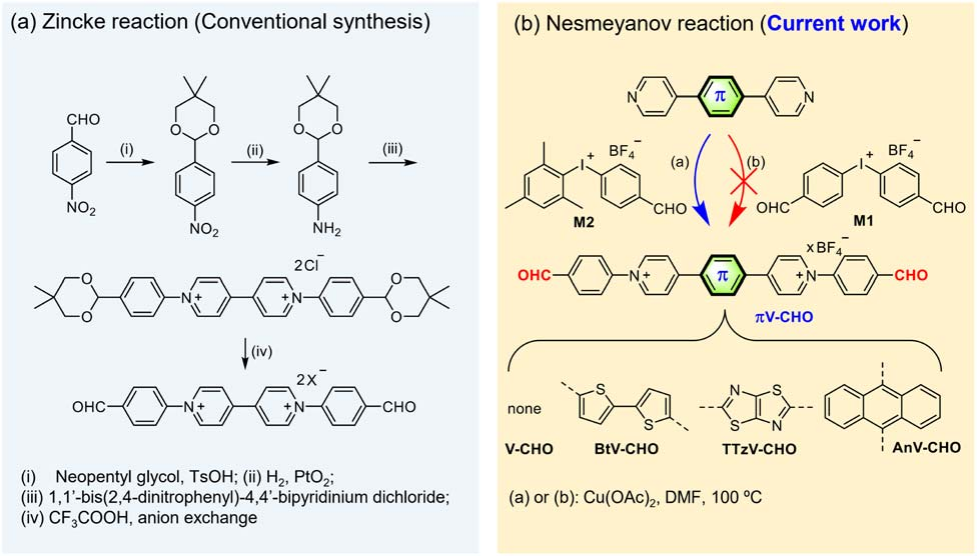
Synthesis of CHO-functionalized *N*-arylated viologens. (a) Zincke reaction (conventional synthesis) and (b) Nesmeyanov reaction (current work).

Herein, we report a convenient synthesis toward a new class of aldehyde-functionalized *N*-arylated viologen building blocks (πV-CHO, Scheme 1). It should be noted that it is inherently very challenging to incorporate aldehyde groups within electron-deficient viologen building blocks, and only one, fairly complex multi-step synthesis has been reported to date. Our new procedure, on the other hand, is simple and versatile, allowing us to access to an entire library of core-extended viologens with tunable optoelectronic properties. Furthermore, we utilize dynamic covalent bonding to build a series of viologen-integrated redox-active polymers, that can be readily solution-processed into high-quality EC films. These obtained films exhibit promising electrochromic properties, such as low driving voltage, fast switching times, great coloration efficiency, remarkably high optical contrast, long cycling stability, and color controls. We believe that this solution-process approach toward viologen-embedded polymers opens a new pathway to functional films for diverse purposes.

## Results and discussion

### Design and synthesis of building blocks

Literature reports on CHO-substituted, *N*-arylated viologens are very limited, and only one example with 4,4′-bipyridinium central core has been reported to date.^21–27^ However, this synthesis involves the utilization of the classic Zincke reaction and tedious multistep protection–deprotection sequence (Scheme 1a). To the best of our knowledge, CHO-functionalized, *N*-arylated viologens with core-extended skeletons have not been reported. The lack of examples could be attributed to the fact that *N*-arylated electron-deficient “π-extended viologens” are difficult to synthesize by the Zincke route.^15^

Previous work by us and others has demonstrated that the Cu(ii)-catalyzed Nesmeyanov reaction *via* diaryliodonium salts is a powerful protocol for the single-step preparation of *N*-arylated viologens.^15,28–30^ This approach provides an accessible means to prepare π-extended viologens with electron-deficient *N*-aryl substitutions. Based on the previous work, we first attempted the quaternization of 4,4′-bipyridine with symmetric bis(4-formylphenyl)iodonium tetrafluoroborate (M1) in the presence of Cu(OAc)_2_ as the catalyst in DMF at 100 °C for 24 h (Scheme 1b). However, the desired product was not obtained. We ascribe this observation to the reduced reactivity of the electron-efficient iodine(iii) reagent and its premature decomposition in solution.^15^

To overcome the decomposition challenge, we then focused on utilizing the asymmetric diaryliodonium salt [Mes-I-PhCHO]BF_4_ (M2) (Scheme 1b).^31^ The introduction of an electron-donating mesityl group was expected to increase the stability of diaryliodonium reagent. Moreover, previous literature suggests that the bulky mesityl group could serve as a “dummy ligand” toward a chemoselective reaction.^29,32^ To our satisfaction, the reaction of 4,4′-bipyridine with the asymmetric M2 in the presence of Cu(OAc)_2_ immediately provided the desired product V-CHO in quantitative yield and high purity. Conveniently, due to the high reaction efficiency, no tedious column chromatography is needed, and the product can be isolated by direct precipitation into diethyl ether. Fig. S1† illustrates the reaction mechanism, involving the typical mechanism for a Cu-catalyzed *N*-arylation by diaryliodonium salts through Cu^III^ species, according to Gao.^29^ The side product 2,4,6-trimethyliodobenzene (MesI) was identified by single-crystal X-ray diffractometry (Fig. S1†) and also characterized by ^1^H NMR spectroscopy (Fig. S2†), supporting the proposed mechanism.

Subsequently, we tested the scope of the *N*-arylation with a range of π-extended pyridines bridged with different linkers, such as anthracene (An), the electron-withdrawing thiazolothiazole (TTz) and the electron-donating bithiophene (Bt). The reactivities are unaffected by the aromatic linker. The Cu(ii)-catalyzed process transfers the PhCHO functional groups to the *N*-atoms with excellent chemoselectivity and in excellent yields (95–100%). Even for the synthesis of TTzV-CHO containing the electron-deficient TTz linker, the reaction is completed within 24 hours, indicating its fast reaction kinetics. All compounds were characterized by ^1^H and ^13^C NMR spectroscopy, as well as high-resolution mass spectrometry (details in the ESI†). Overall, we provide an unprecedented, straightforward and valuable approach for synthesis of a family of CHO-functionalized *N*-arylated π-extended viologens in very high yield.

### Electrochemical and photophysical properties of πV-CHO

**Fig. 1:**
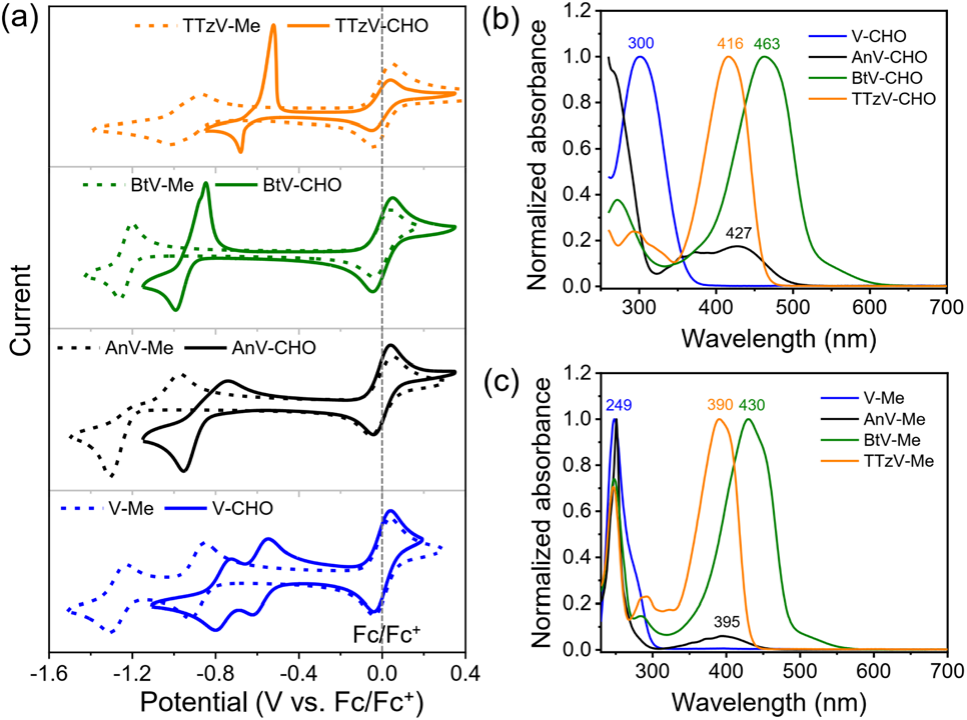
Electrochemical and photophysical properties of πV-CHO and πV-Me. (a) CV and (b), (c) normalized UV-vis spectra of the πV-CHO series and their *N*-methylated congeners πV-Me. CVs were conducted in DMF solution (*c* = 1 mM) with 0.1 M TBAPF_6_ as the electrolyte. UV-vis spectra were measured in CH_3_CN.

Cyclic voltammetry (CV) experiments were carried out to investigate the electrochemical properties of the πV-CHO building blocks. The CV profiles, reduction potentials and reversibility of the redox events were found to depend strongly on the central viologen cores (Fig. 1a). The results are summarized in Table S1.†V-CHO shows two successive reversible reduction events in DMF (*E*_red,1_ = −0.59 V and *E*_red,2_ = −0.76 V, *vs.* Fc/Fc^+^). By introducing bridging π-linkers between the two terminal pyridinium groups, the other three linear πV-CHO species exhibit single-step two-electron reduction processes, due to the loss of electronic communication between the two electroactive sites.^32,33^ The reduction potentials follow the trend BtV-CHO (*E*_red_ = −0.92 V *vs.* Fc/Fc^+^) < AnV-CHO (*E*_red_ = −0.85 V *vs.* Fc/Fc^+^) < TTzV-CHO (*E*_red_ = −0.60 V *vs.* Fc/Fc^+^). This trend is consistent with the electron-accepting properties of the π-linkers. The redox signals of πV-CHO are retained very well, when using various scanning speeds from 100 mV s^−1^ to 1000 mV s^−1^, indicating excellent electrochemical reversibility (Fig. S3†). When compared to their *N*,*N*′-dimethylated counterparts (πV-Me: V-Me, AnV-Me, BtV-Me and TTzV-Me, structures shown in Fig. S4†), the electron injection in πV-CHO is facilitated by 0.3–0.4 V, as evidenced by higher reduction potentials (Fig. 1a and Table S1†). These results demonstrate that the new πV-CHO building blocks display strong electron-acceptor character, which will be highly beneficial for electrochromic applications with low-driving voltages.

The photophysical properties of πV-CHO can also effectively be tuned by the central π linkers (Fig. 1b). Compared to V-CHO (*λ*_max_ = 300 nm), the extended conjugated species show lower-energy absorption bands (AnV-CHO: *λ*_max_ = 427 nm; BtV-CHO: *λ*_max_ = 463 nm; TTzV-CHO: *λ*_max_ = 416 nm). The electron-donating bithiophene linker has the strongest influence on the optical properties with a red shift of Δ*λ*_max_ = 163 nm, and its shallow absorption band was attributed to intramolecular charge transfer. The HOMO–LUMO energy gaps (*E*_g_) were estimated from the absorption onset of UV-vis absorption spectra (Fig. S4†) to be 3.44 eV (V-CHO) > 2.66 eV (TTzV-CHO) > 2.51 eV (AnV-CHO) > 2.34 eV (BtV-CHO). Meanwhile, the *N*-arylated species display an obvious red shift in their absorption maxima by 20–50 nm and smaller HOMO–LUMO energy gap than πV-Me (Fig. 1c and S5†), as a result of the peripheral extension of conjugation.

### Synthesis and characterization of IPPs

With the new series of πV-CHO in hand, the reactivity of CHO-groups with primary amine and acylhydrazine were then tested (details in the ESI†). Reaction with *p*-toluidine or benzohydrazide leads to the corresponding imine or acylhydrazone species in high yield, respectively. Notably, condensation reactions between linear πV-CHO and benzohydrazide with a molar ratio of 1 : 2 is completed within several hours, even at room temperature, supporting the high efficiency of the process (Fig. S6–S8†).

**Fig. 2:**
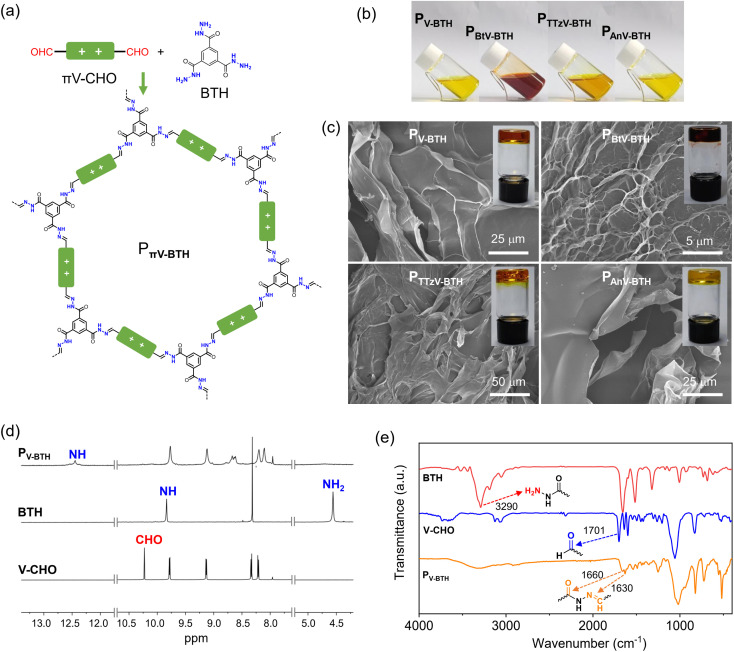
Characterization of P_πV-BTH_. (a) Synthesis and structure of P_πV-BTH_. (b) Pictures of four P_πV-BTH_ solutions in DMSO (2 mg mL^−1^ based on πV-CHO). (c) SEM images of xerogels based on P_V-BTH_, P_BtV-BTH_, P_TTzV-BTH_ and P_AnV-BTH_ formed in DMSO (10 mg mL^−1^ based on πV-CHO). Insets show the photographs of the corresponding P_πV-BTH_ organogels. (d) ^1^H NMR and (e) IR spectra of V-CHO, BTH and P_V-BTH_.

Encouraged by the facile formation of stable hydrazones, we exploited the preparation of IPPs by combination of πV-CHO with a trigonal benzene-1,3,5-tricarbohydrazide (BTH), as shown in Fig. 2a. Upon mixing the linear πV-CHO and BTH precursors in DMSO at a molar ratio of 3 : 2, the solution color immediately turned darker, indicating a rapid reaction between these monomers. The fluidity of the solution was found to depend on the overall concentration of the two precursors πV-CHO and BTH. At low concentration (2 mg mL^−1^ based on πV-CHO), the obtained solutions of P_πV-BTH_ retain homogeneity (Fig. 2b) even over several weeks. At higher concentrations (10 mg mL^−1^ based on πV-CHO), transparent organogels formed after several hours, even at room temperature (Fig. 2c). Scanning Electron Microscopy (SEM) analysis of the four P_πV-BTH_ xerogels clearly reveals 2D morphologies (Fig. 2c). We found that the formed organogels do not revert back to the solution state even upon heating or diluting the mixture. Importantly, gelation was not observed at low concentration (2 mg mL^−1^ based on πV-CHO) even after one month. The above concentration-dependent sol/gel states with different fluidity and solubility can be attributed to the formation of oligomers (or molecular cages) at low concentration and polymers with higher degree of polymerization (DP) at high concentration. We tentatively attribute the transformation from sol to gel to the reconstruction of the dynamic acylhydrazone bond that is facilitated by increased concentration.

To verify the formation of the above species, the reactivity was monitored using ^1^H NMR by combining the πV-CHO and BTH precursors in d6-DMSO at a molar ratio of 3 : 2 after heating at 50 °C for 24 h. At low concentration of πV-CHO (2 mg mL^−1^), a new set of sharp resonances was observed for P_πV-BTH_. As shown in Fig. 2d, the characteristic ^1^H NMR peaks of CHO at 10.22 ppm and NH_2_ at 4.56 ppm from the two precursors (V-CHO and BTH) completely disappear. Instead, a new peak at 8.65 ppm resulting from the of CH=N group of P_V-BTH_ appears, along with a pronounced downfield shift of the NH group from 9.84 ppm (for BTH) to 12.4 ppm (for P_V-BTH_). Similar studies on the other three P_πV-BTH_ polymers (Fig. S9–S12†) support the high efficiency of the polymerization process. Moreover, the ^1^H NMR spectra of P_πV-BTH_ are consistent with their small molecular models (Fig. S6–S8†). The above results suggest a complete reaction between two precursors and the well-resolved NMR signals suggest the formation of oligomers or molecular cage, based on previous research.^33,34^ In contrast, the ^1^H NMR spectrum of the organogel in d6-DMSO exhibits broad and non-discernable peaks, probably as a result of formation of a polymer with higher DP and stronger π–π interactions (Fig. S13†). The identity of the P_πV-BTH_ polymers was also confirmed by FT-IR. As shown in Fig. 2e, the FT-IR spectra of the four P_V-BTH_ show the disappearance of the N–H stretching band of BTH at 3290 cm^−1^ and stretching vibration of aldehydes of V-CHO at *ca.* 1700 cm^−1^, supporting the completeness of the reaction. New peaks at *ca.* 1630 cm^−1^ (C=N) and 1660 cm^−1^ (C=O) belonging to the acylhydrazone bonds were observed instead. Similar changes were also observed in the IR spectra for other three P_πV-BTH_ polymers (Fig. S14†).

Overall, the πV-CHO species are highly valuable building blocks. The presence of the aldehyde groups allows reaction with BTH to efficiently form dynamic acylhydrazone bonds under mild conditions. At low concentration, the dynamic covalent chemistry leads to dispersed oligomers or molecular cages, which has been well-established in the literature.^33–36^ In contrast, at high concentration, the reversible cleavage/formation of dynamic bonds forms polymers with high DP. This supports the above concentration-dependent sol and gel states. It is worth noting that P_πV-BTH_ displays good stability in DMSO, and no precipitation is observed during the entire synthesis, which is in stark contrast to many reported neutral porous polymers, such as COFs.^7–12^ Since no aliphatic chains commonly used to increase the solubility are present, we attribute the high solubility of the material to the presence of cationic charges that provide the colloidal stability through electrostatic repulsion.

### Fabrication of electrochromic films

**Fig. 3:**
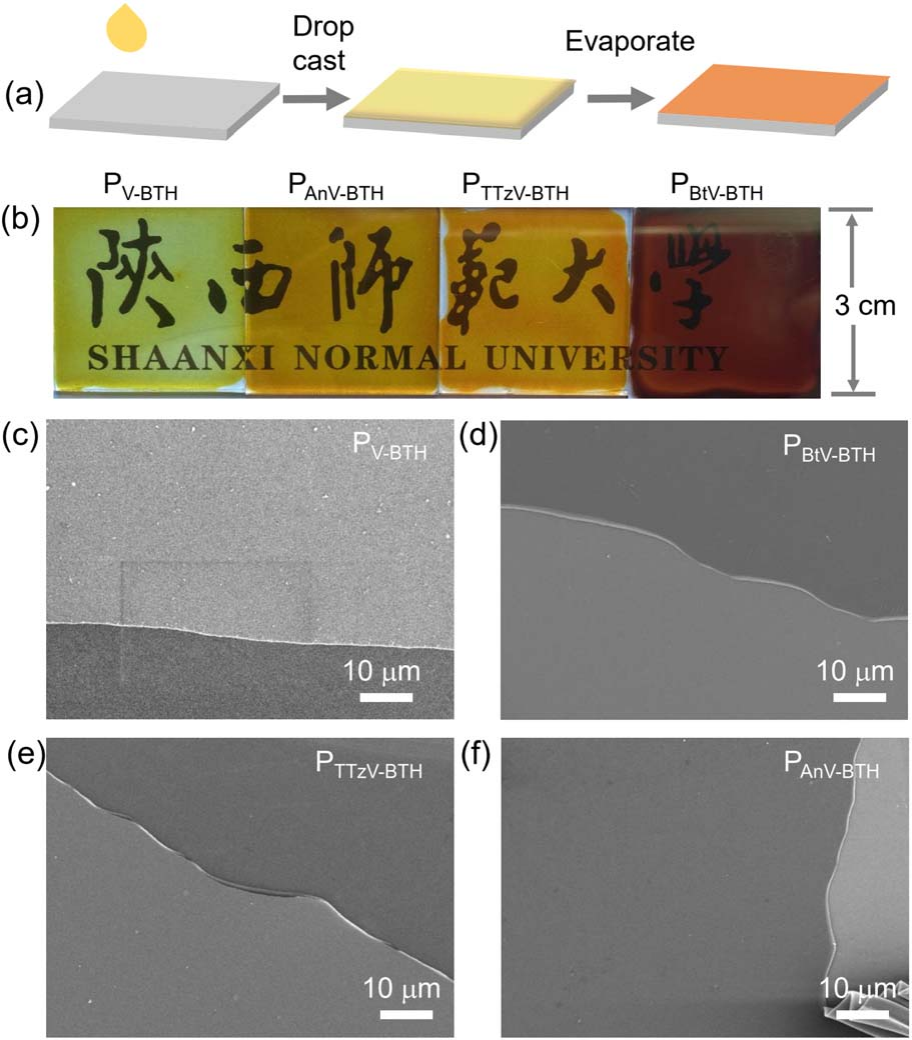
Fabrication of P_πV-BTH_ films. (a) Schematic illustration of the preparation of the P_πV-BTH_ electrochromic thin film. (b) Pictures and (c)–(f) SEM images of the four prepared thin films.

As shown in Fig. 3a, the EC films were prepared by drop-casting a solution of oligomeric P_πV-BTH_ (2 mg mL^−1^ in DMSO) onto a conductive fluorine-doped tin oxide (FTO) electrode, followed by solvent evaporation under heating. Upon slow evaporation, a viscous gel layer was observed on the FTO surface. This phenomenon could be attributed to the formation of cross-linked polymer with high DP, similar to the above concentration-dependent sol and gel states in solution. This strategy also allows easy fabrication of thin-films at large scale (10 cm × 10 cm, Fig. S15†). Although such solution-based fabrication of a high-quality film by the surface sol–gel process has been reported on inorganic metal oxide,^37^ it is unprecedented for organic polymer systems.

To our satisfaction, the obtained films were found to be transparent, strongly adhere to the FTO surface, and are resistant to delamination in the presence of solvents (Fig. 3b). As confirmed by SEM (Fig. 3c–f), the prepared films are highly uniform and without any defects. Their thickness was measured to be 400–500 nm, when the areal mass weights were 0.1 mg cm^−2^ (Fig. S16†). Water contact angles for the four P_πV-BTH_ films were measured to be 54–76°, supporting their hydrophilic character (Fig. S17†). X-ray diffraction (XRD) patterns of all the films show broad peaks at 20–50°, indicating some degree of amorphous morphology (Fig. S18†), and energy dispersive X-ray (EDX) elemental mapping showed uniform distribution for the element composition over the entire films (Fig. S19–S21†). Compared to πV-CHO, the UV-vis spectra of the P_πV-BTH_ films generally exhibit obvious red shifts, consistent with an extended network structure (Fig. S22†). High-resolution TEM (HRTEM) analysis of four P_πV-BTH_ films reveals the presence of nanopores (Fig. S23†). Brunauer–Emmett–Teller (BET) surface areas of P_V-BTH_ film CONs and COTs were found to be less than 1 m^2^ g^−1^ (Fig. S24†). The low surface areas of the P_πV-BTH_ films can attributed to the following two reasons: (i) viologen-based ionic porous polymers in the bulk state typically have low surface areas (less than 50 m^2^ g^−1^), due to the pores being blocked by counter anions;^38–40^ (ii) recent reports have shown that 2D nanoporous films synthesized through a DMSO/air interface have low surface areas (10–30 m^2^ g^−1^), due to the flexible linkers as well as the strong intermolecular interactions of film prepared on 2D surface.^41–43^

### Electrochromic properties of P_V-BTH_ films

**Fig. 4:**
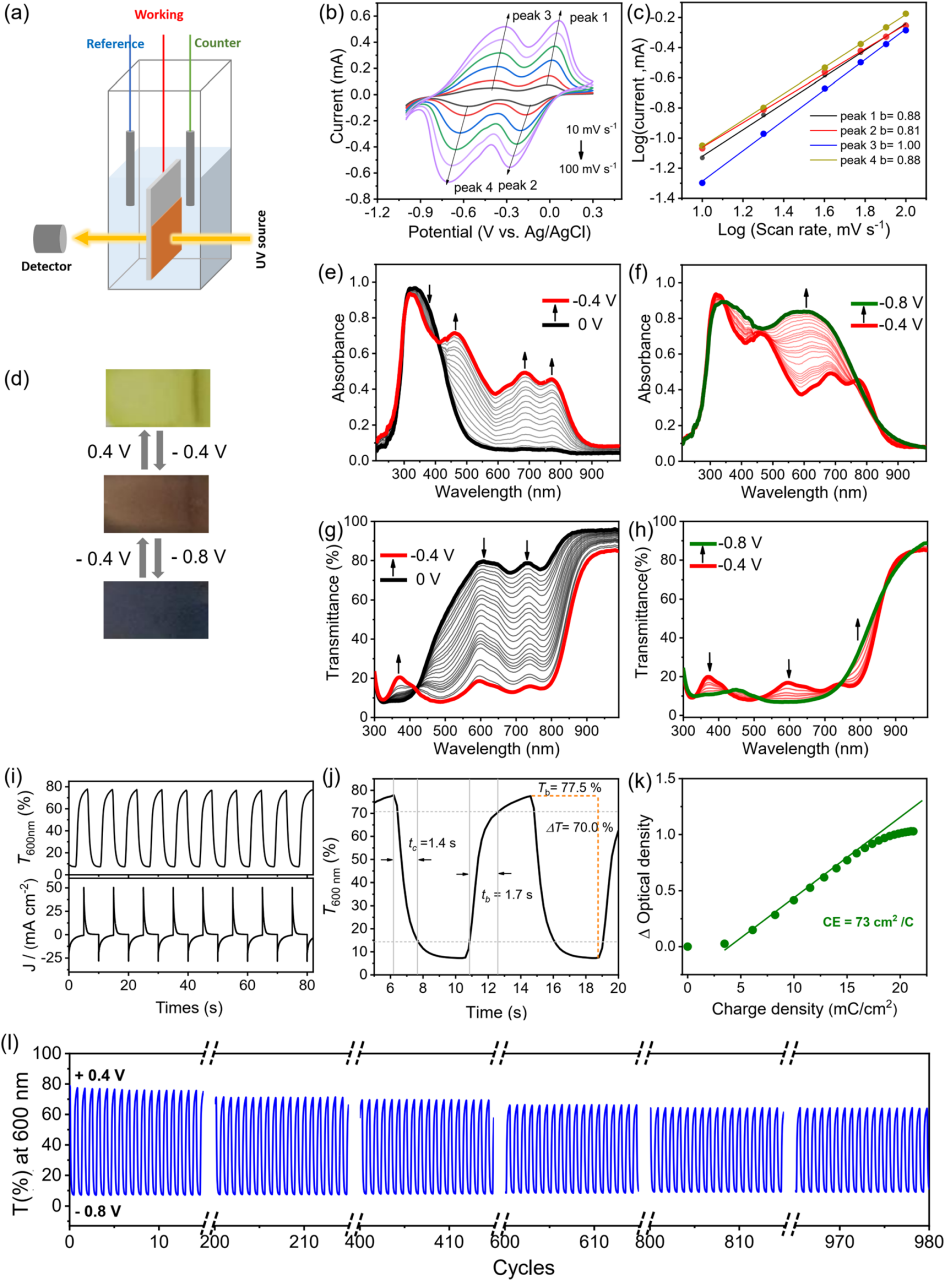
Electrochromic characterization of P_V-BTH_ film. (a) Schematic illustration of the electrochemical and *in situ* spectroelectrochemical measurements. (b) CV curves of the P_V-BTH_ film measured in 0.1 M LiCl aqueous electrolyte at scan rates of 10, 20, 40, 60, 80, and 100 mV s^−1^. (c) Log(*i*) *versus* log(*v*) plots to determine the *b* values of different peaks. (d) Pictures showing the reversible color changes of P_V-BTH_ EC film. (e), (f) UV-vis and (g), (h) transmittance spectra changes of the P_V-BTH_ film in 0.1 M LiCl aqueous electrolyte recorded during the spectroelectrochemistry. The applied potentials are referenced to Ag/AgCl. (i) Plots of transmittance at 600 nm and current *vs.* time with alternating voltage of −0.8 V and +0.4 V. (j) Coloration/bleaching transmission spectrum of the P_V-BTH_ film at 600 nm with an applied voltage of −0.8 V and +0.4 V. (k) Plots of the optical density *vs.* charge density and the slope as coloration efficiency. (l) Cycling stability of the P_V-BTH_ film between −0.8 V and +0.4 V.

The electrochromic properties of the FTO-supported P_V-BTH_ film were then evaluated in a typical three-electrode system for the electrochemical and spectroelectrochemical experiments (Fig. 4a). Cyclic voltammetry (CV) of the P_V-BTH_ film was performed in 0.1 M aqueous LiCl electrolyte with Pt mesh as counter electrode and Ag/AgCl as reference electrode (Fig. 4b). The P_V-BTH_ film showed two reversible reduction peaks at −0.09 and −0.50 V (*vs.* Ag/AgCl) over a potential range of 0.3 to −1.0 V, leading to the formation of the radial cation (V˙^+^) and neutral species (V), respectively.

It is important to note that the LiClO_4_/propylene carbonate (PC) electrolyte solution, commonly used for 2D COF films with neutral skeleton, is not suitable for our materials. As shown in Fig. S25,† the CV of the P_V-BTH_ film exhibits a broad redox pair at −1.11/0.35 V with a large peak-to-peak separation (1.45 V), indicating high polarization, probably due to the slow diffusion of Li^+^ through the film pores in the organic solvent. We surmise that in an aqueous electrolyte, Li^+^ ions could efficiently pass through the hydrophilic pores of the film to access the cationic viologen moieties for the redox reactions more effectively.

To better understand the electrochemical kinetics, the CVs of the P_V-BTH_ film at different scan rates were recorded. Upon increasing the scan rates from 10 to 100 mV s^−1^, the current response also increases, and all multiple redox profiles retain very well, supporting desirable electrochemical stability. According to the power law (*i* = *av*^*b*^), where *i* represents the current of the CV profile, *v* is the sweep rate, and *a* and *b* are adjustable parameters.^44^ It has been suggested that the charge-storage process is mainly dependent on the *b* value. If *b* equals 0.5, the process can be considered faradaic, while if *b* has a value of 1, the process is capacitive. As shown in Fig. 4c, the linear fit of log(*v*) and log(*i*) reveals slopes in the range of 0.8–1.0 for *b* of both the anodic and cathodic peaks that indicates capacitance-dominant kinetics for the electrochromic chemistry. This result supports fast ion transport that benefits from the porous structure of the film.

Electrochemical reduction of the P_V-BTH_ film causes a gradual color change from initially pale yellow (V^2+^) to brown (V˙^+^), and then to dark grey/black (V), as shown in Fig. 4d. The corresponding change in the UV-vis spectra upon stepwise reduction were studied by *in situ* spectroelectrochemistry. As shown in Fig. 4e and f, the pristine P_V-BTH_ film is pale yellow and transparent, exhibiting an intense absorption peak at 333 nm. Applying potentials from 0 V to −0.4 V leads to the gradual formation of intense peaks at 483, 685 and 773 nm. During the process, a clear isosbestic point was observed at 410 nm, indicating the clean and gradual generation of a single new species. This process corresponds to the first reduction of the viologen moiety, leading to the formation of radical cation (V˙^+^). When the applied potential was further adjusted from −0.4 V to −0.8 V, an intense new band emerged at around 610 nm, while the characteristic peaks for the radical cation significantly decrease. These spectral changes can be attributed to the second reduction of viologen, forming the neutral species (V). Importantly, the electrochromic reduction is reversible, and the initial P_V-BTH_ spectrum can be recovered by reversing the applied voltage back to +0.4 V.

**Fig. 5:**
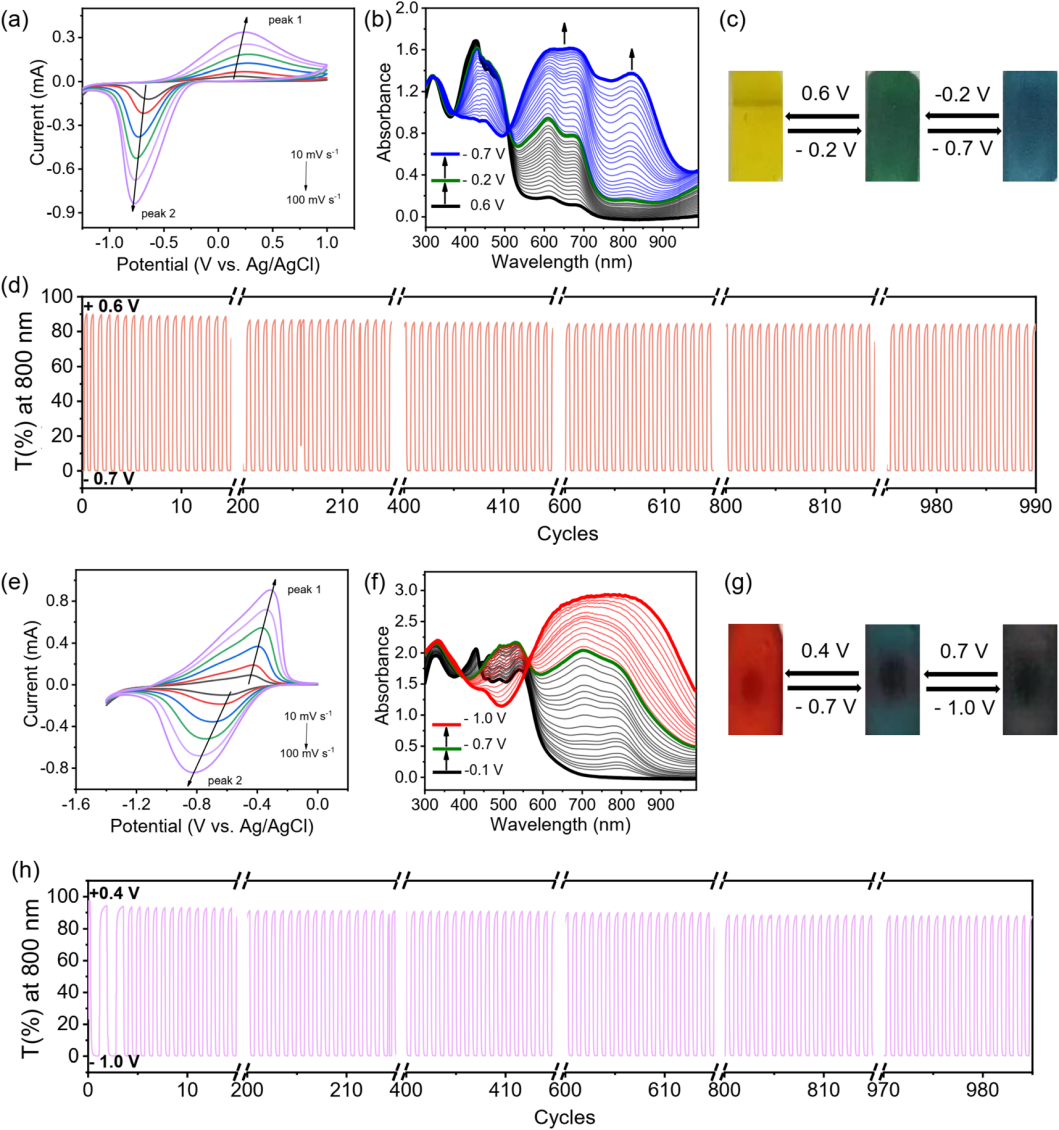
Electrochromic characterization of P_TTzV-BTH_ and P_BtV-BTH_ films. (a) CV curves of the P_TTzV-BTH_ films measured in 0.1 M aqueous LiCl electrolyte at scan rates of 10, 20, 40, 60, 80, and 100 mV s^−1^. (b) UV-vis spectra changes of an P_TTzV-BTH_ film in 0.1 M aqueous LiCl electrolyte recorded during spectroelectrochemistry. (c) Pictures showing the reversible color changes of an P_TTzV-BTH_ EC film. (d) Cycling stability of an P_TTzV-BTH_ film between −0.7 V and +0.6 V. (e) CVs of an P_BtV-BTH_ film measured in 0.1 M aqueous LiCl electrolyte at scan rates of 10, 20, 40, 60, 80, and 100 mV s^−1^. (f) UV-vis spectral changes of an P_BtV-BTH_ film in 0.1 M aqueous LiCl electrolyte recorded during spectroelectrochemistry. (g) Pictures showing the reversible color changes of the EC P_BtV-BTH_ film. (h) Cycling stability of an P_BtV-BTH_ film between −1.0 V and +0.4 V.

These changes are also clearly observed in the corresponding transmission spectra (Fig. 4g and h). By switching the voltages between +0.4 V and −0.8 V (*vs.* Ag/AgCl), the initial contrast ratios (Δ*T*%) between yellow and dark states was determined to be 70.0% at 600 nm. This value only dropped only by *ca.* 14% after 1000 cycles, suggesting excellent stability of the EC P_V-BTH_ film. The response time for the switching is determined by Δ*T*% experiments when the contrast ratio reaches over 90% of its maximum between bleached and colored states. The coloration (*t*_c_) and bleaching (*t*_b_) time of P_V-BTH_ film at 600 nm were calculated to be 1.4 s and 1.7 s, respectively (Fig. 5j). The coloration efficiency (CE) at 600 nm was calculated to be 73 cm^2^ C^−1^, respectively, according to the equation.
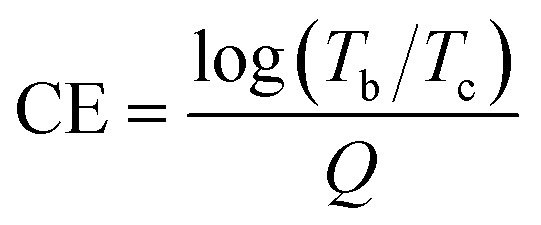
where *Q* is the charge density.

### Dependence of the electrochromism on the viologen skeleton

The electrochromic properties can be readily tailored by the viologen skeleton. In contrast to the P_V-BTH_ film, the other three P_πV-BTH_ films incorporating TTzV, BtV and AnV exhibit one-step two-electron reduction peaks under aqueous conditions. These observations are in line with the CVs of their corresponding πV-CHO precursors (Fig. 1), as well as other previously reported small molecules with similar skeletons.^17–19^ As shown in Fig. 5, S26 and S27,† the redox couples for P_TTzV-BTH_ and P_BtV-BTH_ were determined to be −0.68/0.24 V and −0.64/−0.43 V (*vs.* Ag/AgCl) at 20 mV s^−1^. The *b* values of both anodic and cathodic peaks were determined to be in the range of 0.83–1.0, indicating capacitance-dominant kinetics of the electrochromic chemistry here as well. Upon reduction, P_TTzV-BTH_ and P_BtV-BTH_ films also exhibit dramatic color changes. However, due to the large steric effects from bulky anthracene linker, the radical species cannot be properly delocalized throughout the anthracene moiety, and hence, the no obvious electrochromic behavior was observed for the P_AnV-BTH_ film (Fig. S28†).

For P_TTzV-BTH_ (Fig. 5a–d), three reversible color states, yellow (TTzV^2+^), green (TTzV˙^+^) and blue (TTzV), are observed during electrochemical cycling and by the spectroelectrochemistry. The first reduction from TTzV^2+^ to TTzV˙^+^ by applying potentials from +0.6 V to −0.2 V, leads to the formation of new absorption bands at 609 and 677 nm. Upon further reduction (−0.2 V to −0.7 V), a strong absorption band appears at 820 nm, characteristic of the TTzV neutral species. It should be noted that the characteristic peaks of TTzV˙^+^ also continue to increase during further reduction, due to the closely spaced two-electron reduction processes that essentially occur in parallel during the spectroelectrochemistry experiments.

For the P_BtV-BTH_ film (Fig. 5e–h), formation of the reduced species is accompanied by a color change from the initial red color, to deep green and then to dark grey/black. Reduction to the radical cation gives rise to a new absorption band in the 600–950 nm range that continues to grow with increasing negative voltages, supporting the continued presence of the radical cation that is likely re-generated during the process *via* comproportionation.

The EC properties of the three P_πV-BTH_ films are summarized in Table 1. All films display fast response times (coloration and bleaching) of less than 2 s. The two films of core-extended viologen-based IPPs display improved Δ*T*%, cycling stability, and high color efficiency. The P_TTzV-BTH_ and P_BtV-BTH_ films have initial contrast ratios (Δ*T*%) of 89.8% and 95.6%. These values were reduced by only 6% and 7% after 1000 consecutive on-off switching cycles, demonstrating their excellent long-term cycling stability. Their outstanding cycling performance is attributed to the non-fluidity of viologen in rigid film that reduces the possibility of dimerization. The color efficiencies of the EC P_TTzV-BTH_ and P_BtV-BTH_ films were 268 and 235 cm^2^ C^−1^, a three-fold increase over that of P_V-BTH_ (73 cm^2^ C^−1^). The improved color efficiencies of P_TTzV-BTH_ and P_BtV-BTH_ films can be partially attributed to the reduction-induced large spectra modulation, as a result of large conjugated viologen centers. We also found that P_TTzV-BTH_ and P_BtV-BTH_ films had improved electronic conductivity over that of the P_V-BTH_ film with shorter conjugated backbone. As shown in the electrochemical impedance spectroscopy (EIS), the P_TTzV-BTH_ and P_BtV-BTH_ films have smaller charge transfer resistance (Fig. S29†). Overall, we demonstrate a class of easily processible films that exhibit excellent EC performances that are superior to those of the state-of-the-art 2D COFs and comparable with many reported viologen-based EC materials (Table S2†).^5^ These foundational results indicate that there is plenty of room to further improve the EC performance, by tuning the central viologen cores and the amine linker.

**Table 1:** ECD performance of P_πV-BTH_ films

EC films	Color^a^ (O)	Color^b^ (R)	*V*_c_/*V*_b_^c^ (V)	*t*_c_/*t*_b_^d^ (s)	Δ*T*%^e^ (at nm)	Stability (cycles/Δ*T*% drop)	CE^f^ (cm^2^ C^−1^)
P_V-BTH_	Yellow	Dark	−0.4/+0.8	1.4/1.7	70.0 (600)	1000/14%	73
P_TTzV-BTH_	Yellow	Blue	−0.7/+0.6	1.0/0.8	89.8 (800)	1000/6%	268
P_BtV-BTH_	Red	Dark	−1.0/+0.4	0.8/1.3	95.6 (800)	1000/7%	235

a Color at oxidized state.

b Color at reduced state.

c Bleaching (*V*_b_) and coloration potential (*V*_c_).

d Bleaching (*t*_b_) and coloration time (*t*_c_).

e Transmittance change during coloration and bleaching process.

f Coloration efficiency.

## Conclusions

In summary, we report a novel design strategy to effectively fabricate redox-active 2D porous polymers, involving a convenient synthesis of aldehyde-functionalized *N*-arylated viologens (πV-CHO) with tunable chemical structures, and the use of dynamic covalent acylhydrazone bonds that offer fast reaction times and provide self-healing. Benefitting from a “charge-induced dispersion”, these 2D ionic porous polymers have excellent stability in DMSO, and importantly, can be readily solution-processed into high-quality EC films. The films exhibit low driving voltages (1.2–1.4 V), fast response times (0.8–1.7 s), excellent coloration efficiencies of 73–268 cm^2^ C^−1^, remarkably high optical contrasts up to 95.6%, and tunable oxidation and reduction colors dependent on their respective viologen cores. We believe that the solution processability, tunable redox and photophysical property as well as intrinsic porosity offered by these IPPs now effectively addresses some of most challenging problems, such as cost factor, large-scale, and simple fabrication in electrochromic applications. This research may also inspire the development of advanced materials for energy storage and photonic devices.

## Author contributions

H. Miao synthesized the materials and performed the electrochromic experiments. L. Chen and F. Xing assisted in the discussion of the electrochromic performance. H. Li helped with the optimization of the film fabrication and electrochemical characterization. T. Baumgartner revised the paper and provided helpful discussion. X. He supervised the project and prepared the manuscript with input from all the other authors.

## Conflicts of interest

There are no conflicts to declare.

## Supplementary Material

SC-015-D4SC01408A-s001
