# Supplementary material for: Viologen-based solution-processable ionic porous polymers for electrochromic applications
Source: Chem Sci. 2024 Apr 23;15(20):7576–85. doi: 10.1039/d4sc01408a (PMC11110146; doi:10.1039/d4sc01408a)
Supplement: SC-015-D4SC01408A-s001 [file SC-015-D4SC01408A-s001.pdf]

Electronic Supplementary Material (ESI) for Chemical Science.
This journal is © The Royal Society of Chemistry 2024

Supplementary Information

Viologen-Based Solution-Processible Ionic Porous Polymers for Electrochromic Applications

Hongya Miao,[a] Ling Chen,[a] Fangfang Xing,[a] Huijie Li,[a] Thomas Baumgartner[b]* and Xiaoming He[a]*

a Key Laboratory of Applied Surface and Colloid Chemistry (Ministry of Education), School of Chemistry and Chemical Engineering, Shaanxi Normal University, Xi'an 710119, P.R. China

b Department of Chemistry, York University, 4700 Keele Street, Toronto, Ontario M3J 1P3, Canada

*Corresponding Authors Email: tbaumgar@yorku.ca, xmhe@snnu.edu.cn

EXPERIMENTAL DETAILS

1. Materials and instruments

All chemical reagents were purchased from commercial sources (Aldrich, Alfa Aesar, Strem and Adamas) and were, unless otherwise noted, used without further purification. 9,10-di(pyridin-4-yl)anthracene,[S1] 5,5'-di(pyridin-4-yl)-2,2'-bithiophene,[S2] 2,5-di(pyridin-4-yl)thiazolo[5,4-*d*]thiazole,[S3] bis(4-formylphenyl)iodonium tetrafluoroborate,[S4] [Mes-I-PhCHO]BF_4[S5] were prepared according to reported procedures.

^{1}H NMR and ^{13}C NMR spectra were recorded on 400 MHz and 600 MHz BRUKER spectrometers. Chemical shifts were referenced to residual non-deuterated solvent peaks. Mass spectrometry data were collected on a Bruker maxis UHR-TOF mass spectrometer in ESI positive mode. Ultraviolet–visible (UV-vis) absorption spectra were recorded on a Cary 3500 spectrometer. Steady-state emission experiments were carried out on a Horiba FluoroMax Plus spectrofluorometer. Scanning electron microscope (SEM) images were recorded using a Hitachi SU8220 system. Powder X-ray diffraction (PXRD) patterns were recorded on a Bruker D8 Advance instrument (CuKα, 40 kV, 40 mA). Thermogravimetric analysis (TGA) was performed under a nitrogen atmosphere from 25 to 800 °C with a heating rate of 10 °C min^{-1}. The X-ray photoelectron spectra (XPS) experiments were carried out on a PHI-5400 electron spectrometer. Cyclic voltammetry (CV) was performed on a Shanghai Chenhua CHI760E instrument, with a polished glassy carbon electrode as the working electrode, a Pt-wire as counter electrode, and Ag/AgCl as a reference electrode, using ferrocene/ferrocenium (Fc/Fc^{+}) as internal standard. CV experiments for small molecules were performed in DMF solution with tetrabutylammonium hexafluorophosphate (0.1 M) as supporting electrolyte. The Brunauer−Emmett−Teller (BET) surface areas and pore size distributions were measured by nitrogen adsorption and desorption at 77.3 K using a Micromeritics ASAP 2460 volumetric adsorption analyzer. The films were detached by soaking in ethanol assisted by sonication for *ca.* 4 hours, followed by drying at 80 °C for 12 h under vacuum (10^{-5} bar) before analysis. Theoretical calculations have been carried out at the B3LYP/6-31G(d,p) level by using the GAUSSIAN 09 suite of programs.[S6]

2. Synthesis of πV-CHO

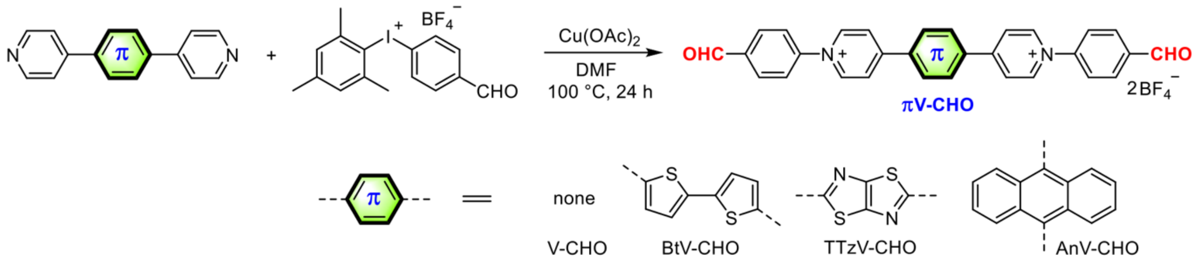

Scheme S1. Synthesis of **πV-CHO**.

Synthesis of V-CHO.

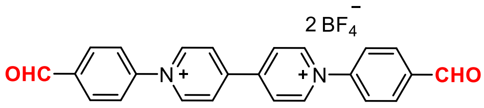

A solution of 4,4'-bipyridine (312 mg, 2 mmol, 1 eq.), [Mes-I-PhCHO]BF_4 (2.63 g, 6 mmol, 3 eq.), $Cu(OAc)_2$ (20 mg, 0.1 mmol, 5 mol %) in DMF (50 mL) was heated for 24 h at 100 °C. After cooling down to room temperature, excess diethyl ether was added to precipitate out the resulting product, which was filtered and further washed with diethyl ether and dried under a vacuum. Yield: 97%. ^{1}H NMR (400 MHz, *d*6-DMSO): δ (ppm) = 10.22 (s, 2H; C*H*O), 9.77 (d, J = 6.6 Hz, 4H; C_5H_4N), 9.12 (d, J = 6.6 Hz, 4H; C_5H_4N), 8.32 (d, J = 8.4 Hz, 4H; C_6H_4), 8.20 (d, J = 8.4 Hz, 4H; C_6H_4). ^{13}C NMR (100 MHz, *d*6-DMSO): δ (ppm) = 192.86, 154.79, 146.37, 145.97, 138.10, 131.45, 126.42, 125.59. ESI-MS: m/z calculated for $C_{24}H_{18}N_2O_2^{2+}$ [M-2BF$_4$]$^{2+}$ 183.0679, found 183.0681.

Synthesis of AnV-CHO.

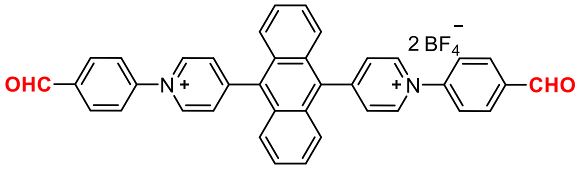

This was prepared according to the procedure for **V-CHO** except 9,10-di(pyridin-4-yl)anthracene (664 mg, 2 mmol) was used instead of 4,4'-bipyridine. Yield: 95 %. ^{1}H NMR (400 MHz, *d*6-DMSO): δ (ppm) = 10.25 (s, 2H; C*H*O), 9.68 (d, J = 6.4 Hz, 4H; C_5H_4N), 8.55 (d, J = 6.4 Hz, 4H; C_5H_4N), 8.39 (d, J = 8.4 Hz, 4H; C_6H_4), 8.29 (d, J = 8.4 Hz, 4H; C_6H_4), 7.84 (m, 4H;

anthracene Ar*H*), 7.70 (m, 4H; anthracene Ar*H*). ^{13}C NMR (100 MHz, *d*6-DMSO): δ (ppm) = 192.89, 157.52, 146.86, 145.77, 138.20, 133.46, 131.61, 130.97, 128.55, 127.86, 126.60, 126.43. ESI-MS: m/z calculated for $C_{38}H_{26}N_2O_2^{2+}$ $[M-2BF_4]^{2+}$ 271.0992, found 271.0996.

Synthesis of BtV-CHO.

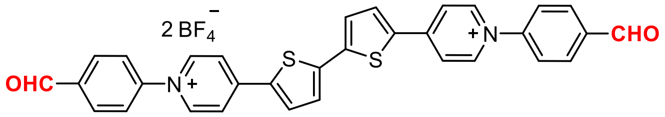

This was prepared according to the procedure for **V-CHO** except 5,5'-di(pyridin-4-yl)-2,2'-bithiophene (640 mg, 2 mmol) was used instead of 4,4'-bipyridine. Yield: 94 %. ^{1}H NMR (400 MHz, *d*6-DMSO): δ (ppm) = 10.19 (s, 2H; C*H*O), 9.33 (d, J = 7.2 Hz, 4H; C_5H_4N), 8.57 (m, 6H; C_5H_4N and C_4H_2S), 8.28 (d, J = 8.8 Hz, 4H; C_6H_4), 8.13 (d, J = 8.8 Hz, 4H; C_6H_4), 8.00 (d, J = 4.0 Hz, 2H; C_4H_2S). ^{13}C NMR (100 MHz, *d*6-DMSO): δ (ppm) = 192.78, 148.85, 146.25, 144.99, 143.39, 137.88, 137.75, 135.49, 131.48, 129.86, 126.02, 122.63. ESI-MS: m/z calculated for $C_{32}H_{22}N_2O_2S_2^{2+}$ $[M-2BF_4]^{2+}$ 265.0556, found 265.0556.

Synthesis of TTzV-CHO.

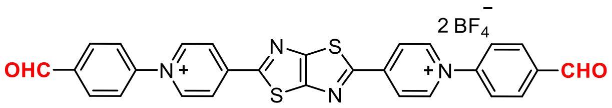

This was prepared according to the procedure for **V-CHO** except 2,5-di(pyridin-4-yl)thiazolo[5,4-*d*]thiazole (592 mg, 2 mmol) was used instead of 4,4'-bipyridine. Yield: 99 %. ^{1}H NMR (400 MHz, *d*6-DMSO): δ (ppm) = 10.21 (s, 2H; C*H*O), 9.59 (d, J = 6.4 Hz, 4H; C_5H_4N), 8.96 (d, J = 6.4 Hz, 4H; C_5H_4N), 8.31 (d, J = 8.0 Hz, 4H; C_6H_4), 8.19 (d, J = 8.0 Hz, 4H; C_6H_4). ^{13}C NMR (100 MHz, *d*6-DMSO): δ (ppm) = 192.86, 165.61, 156.53, 147.53, 146.50, 146.37, 138.20, 131.53, 126.35, 124.55. ESI-MS: m/z calculated for $C_{28}H_{18}N_4O_2S_2^{2+}$ $[M-2BF_4]^{2+}$ 253.0430, found 253.0431.

3. Reactivity of πV-CHO with model amines

3.1 Reaction with p-toluidine

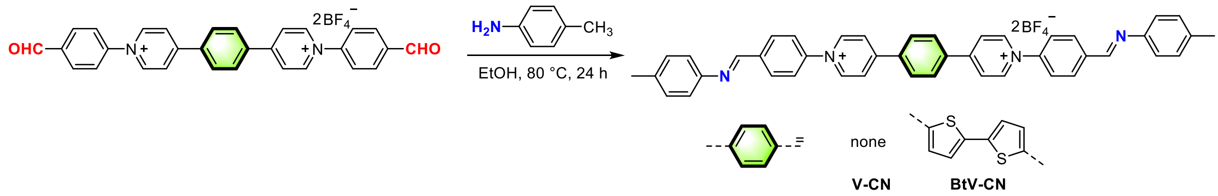

Scheme S2. Synthesis of **V-CN** and **BtV-CN**.

Synthesis of V-CN.

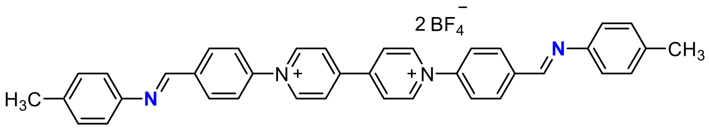

A mixture of **V-CHO** (54 mg, 0.1 mmol, 1 eq.) and p-toluidine (43 mg, 0.4 mmol, 4 eq.) in EtOH (2 mL) was stirred for 24 h at 80 °C. After cooling down to room temperature, excess diethyl ether was added to precipitate out the resulting product, which was filtered and further washed with diethyl ether and dried under a vacuum. Yield: 70 %. ^{1}H NMR (600 MHz, *d*6-DMSO): δ (ppm) = 9.77 (br, 4H; C_5H_4N), 9.11 (br, 4H; C_5H_4N), 8.88 (s, 2H; N=C*H*), 8.33 (br, 4H; C_6H_4), 8.13 (br, 4H; C_6H_4), 7.31 (br, 8H; C_6H_4), 2.37 (s, 6H; CH_3). ^{13}C NMR (150 MHz, *d*6-DMSO): δ (ppm) = 158.35, 148.50, 146.29, 144.06, 139.34, 136.86, 130.41, 130.30, 129.64, 127.19, 125.94, 121.73, 21.11. ESI-MS: m/z calculated for $C_{38}H_{32}N_4^{2+}$ $[M-2BF_4]^{2+}$ 272.1308, found 272.1313.

Synthesis of BtV-CN.

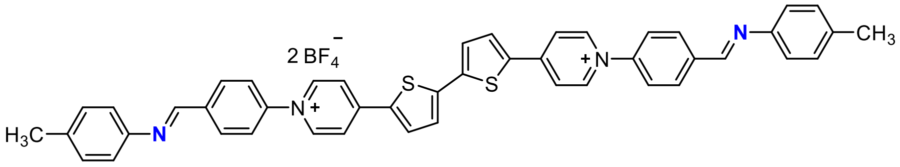

This was prepared according to the procedure for **V-CN** except **BtV-CHO** (70.4 mg, 0.1 mmol) was used instead of **V-CHO**. In addition, DMSO was used as solvent instead of EtOH in order to offer good solubility. Yield: 87 %. ^{1}H NMR (600 MHz, *d*6-DMSO): δ (ppm) = 9.33 (d, J = 6.6 Hz, 4H; C_5H_4N), 8.85 (s, 2H; N=C*H*), 8.56 (m, 6H; C_5H_4N and C_4SH_2), 8.28 (d, J = 9.0 Hz, 4H; C_6H_4), 8.06 (d, J = 9.0 Hz, 4H; C_6H_4), 8.00 (d, J = 4.2 Hz, 2H; C_4SH_2), 7.31 (s, 8H; C_6H_4), 2.36 (s, 6H; CH_3).

3.2 Reaction with benzohydrazide

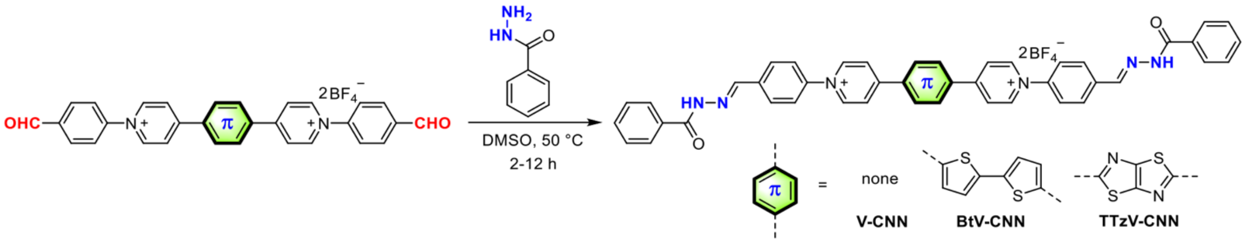

Scheme S3. Synthesis of **V-CNN**, **BtV-CNN**, and **TTzV-CNN**.

General Synthesis of πV-CNN. A mixture of **πV-CHO** (1 eq.) and benzohydrazide (2 eq.) in d6-DMSO was stirred for 2-12 h at 50 °C. The reaction was monitored by ^{1}H NMR (Figure S5-7). Results show that reaction of **πV-CHO** with benzohydrazide is very fast, and can even complete in several hours at room temperature.

4. Experimental procedures

Preparation of $P_{\pi V\text{-}BTH}$ Organogels. πV-CHO and BTH were fully dissolved in DMSO, respectively. Then equimolar BTH solution was added dropwise to mix with the **πV-CHO** solution (10 mg/mL) with gentle stirring. This single precursor solution was maintained undisturbed at 25 °C. After about 4-24 h, transparent organogels were formed.

Fabrication of Electrochromic $P_{\pi V\text{-}BTH}$ Films. πV-CHO (2mg/mL) and BTH were mixed in DMSO at a molar ratio of 3:2, and the solution was stirred at 50 °C for 24 h to ensure the complete reaction. The obtained **$P_{\pi V\text{-}BTH}$** solution was carefully dropped cast on a piece of FTO conductive glass, with a mass loading of 0.1 mg cm^{-2} (based on **πV-CHO**). For example, only 50 μL solution was needed for fabricating a 1cm×1cm film. After slowly evaporation of DMSO upon gentle heating on the hot plate at 50 °C for 3-6 h, a robust and transparent EC films were formed. Note that despite the high boiling point of DMSO (189 °C), the small solvent volume can be easily removed by gentle heating. In addition, the high boiling point of DMSO prevents fast evaporation, which allows formation of a gel layer on the surface to obtain high-quality films.

Electrochemical and Spectroelectrochemical Measurements. Cyclic voltammogram (CV) was performed using a CHI 760E electrochemical workstation by assembly in a standard three-electrode system, where **$P_{\pi V\text{-}BTH}$** film on FTO as the working electrode, a Pt wire counter electrode

and a Ag/AgCl reference electrode. 0.1 M LiCl aqueous solution was used as the electrolyte. The in-situ UV–vis spectroelectrochemical equipment was assembled with an electrochemical workstation and an Ocean optics spectroscopy based on a three-electrode system (Pt wire as the counter electrode, Ag/AgCl as the reference electrode, FTO-supported $\mathbf{P_{\pi V\text{-}BTH}}$ film as the working electrode, and 0.1 M LiCl aqueous electrolyte) in a 1×1 cm^2 quartz cell. The absorption and transmittance spectra of were record upon stepwise reduction/oxidation electrolysis.

5. Supporting Data

Table S1. Optical and Electronic Properties of the CHO-functionalized N-arylated viologens.

Compd	$E_{1/2}$ [V][a]	λ_{max} [nm][b]	λ_{onset} [nm][b]	E_g^{opt} [eV][c] (calcd)[d]	E_{HOMO}/E_{LUMO} [eV][e] (calcd)[d]
**V-CHO**	-0.59, -0.76	300	360	3.44 (3.56)	-7.68/-4.21 (-7.56/-4.00)
**AnV-CHO**	-0.85	427	494	2.51 (2.72)	-6.48/-3.95 (-5.96/-3.24)
**BtV-CHO**	-0.92	463	531	2.34 (2.71)	-6.22/-3.88 (-6.36/-3.65)
**TTzV-CHO**	-0.60	416	466	2.66 (3.10)	-6.82/-4.20 (-7.12/-4.02)
**V-Me**	-0.89, -1.27	249	298	4.16 (5.23)	-8.16/-3.91 (-8.95/-3.72)
**AnV-Me**	-1.14	250, 395	452	2.74 (2.98)	-6.35/-3.66 (-5.92/-2.94)
**BtV-Me**	-1.23	430	490	2.53 (2.86)	-6.15/-3.57 (-6.30/-3.44)
**TTzV-Me**	-0.94	390	434	2.86 (3.26)	-6.83/-3.86 (-7.10/-3.84)

[a]CV in DMF solution with [nBu$_4$N][PF$_6$] as supporting electrolyte (0.1 M), referenced to Fc/Fc$^+$. [b]UV-vis absorption in CH_3CN. [c]Energy gap values (E_g^{opt}) were calculated from the onset wavelengths (λ_{onset}) of the absorption spectra according to $E_g^{opt} = 1240/\lambda_{onset}$. [d]Theoretical calculations have been carried out at the B3LYP/6-31G(d) level with a CPCM solvation model (CH_3CN) by using the GAUSSIAN 09 suite of programs. [e]Energy levels vs vacuum level were calculated from CV data ($E_{LUMO} = -(E^{red}_{onset} + 4.8)$ eV; Fc/Fc$^+$ = -4.8 eV vs vacuum level) and from the optically determined energy gap ($E_{HOMO} = E_{LUMO} - E_g^{opt}$).

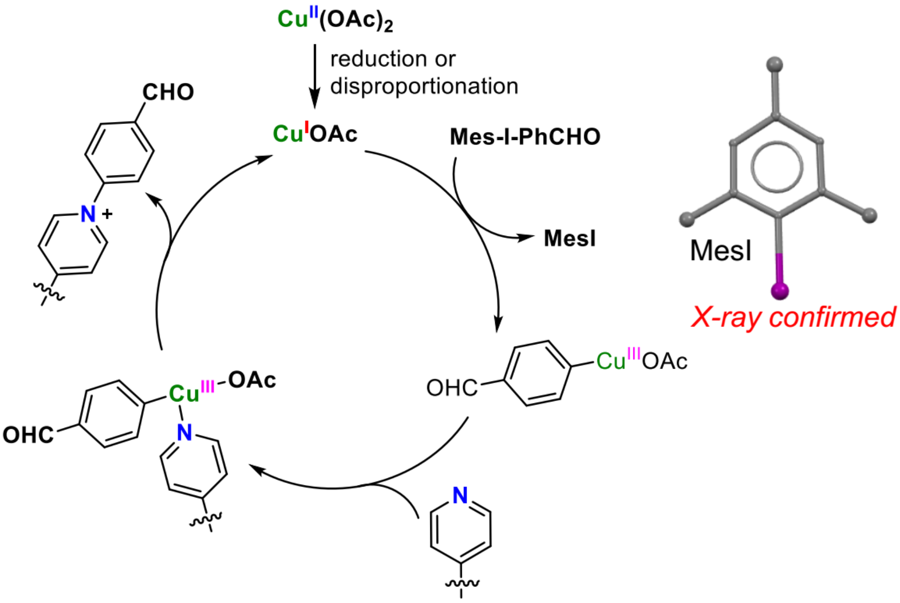

Figure S1. Proposed mechanism for the Cu(II)-catalyzed N-Arylation. MesI crystal was obtained by slow evaporation of the filtrate of reaction mixture.

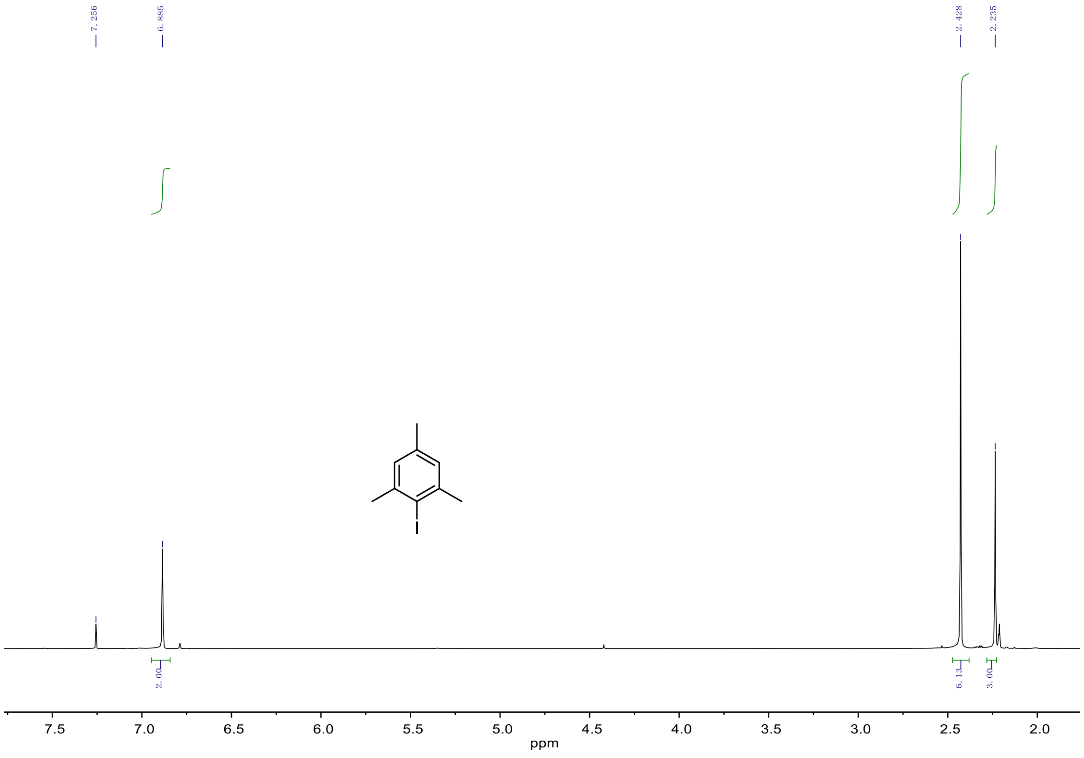

Figure S2. ^{1}H NMR spectra of MesI, that was separated as side product of the Cu(II)-catalyzed N-Arylation.

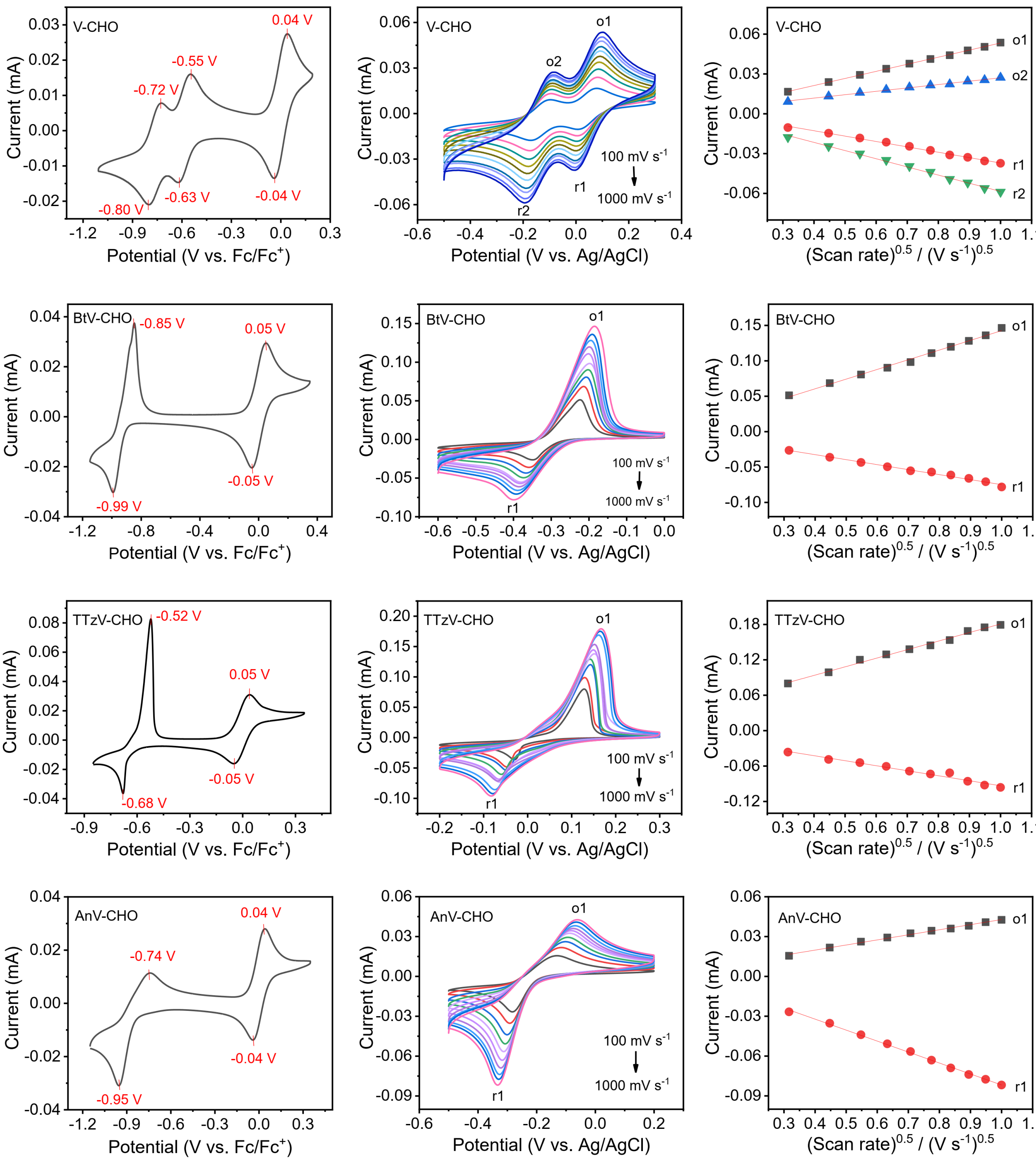

Figure S3. Electrochemistry of **πV-CHO**. (left) CV of **πV-CHO** (1 mM) in DMF solution, referenced to Fc/Fc^{+}. (middle) CV of **πV-CHO** (1 mM) in DMF solution at varying scan rates (100, 200, 300, 400, 500, 600, 700, 800, 900, 1000 mV s^{-1}, referenced to Ag/AgCl; peak height increases with increasing scan rate). (right) Linear plots of peak current *vs.* square root of the scan rate. $n\mathrm{Bu_4PF_6}$ (0.1 M) as supporting electrolyte. From top to bottom: **V-CHO**, **BtV-CHO**, **TTz-CHO** and **AnV-CHO**.

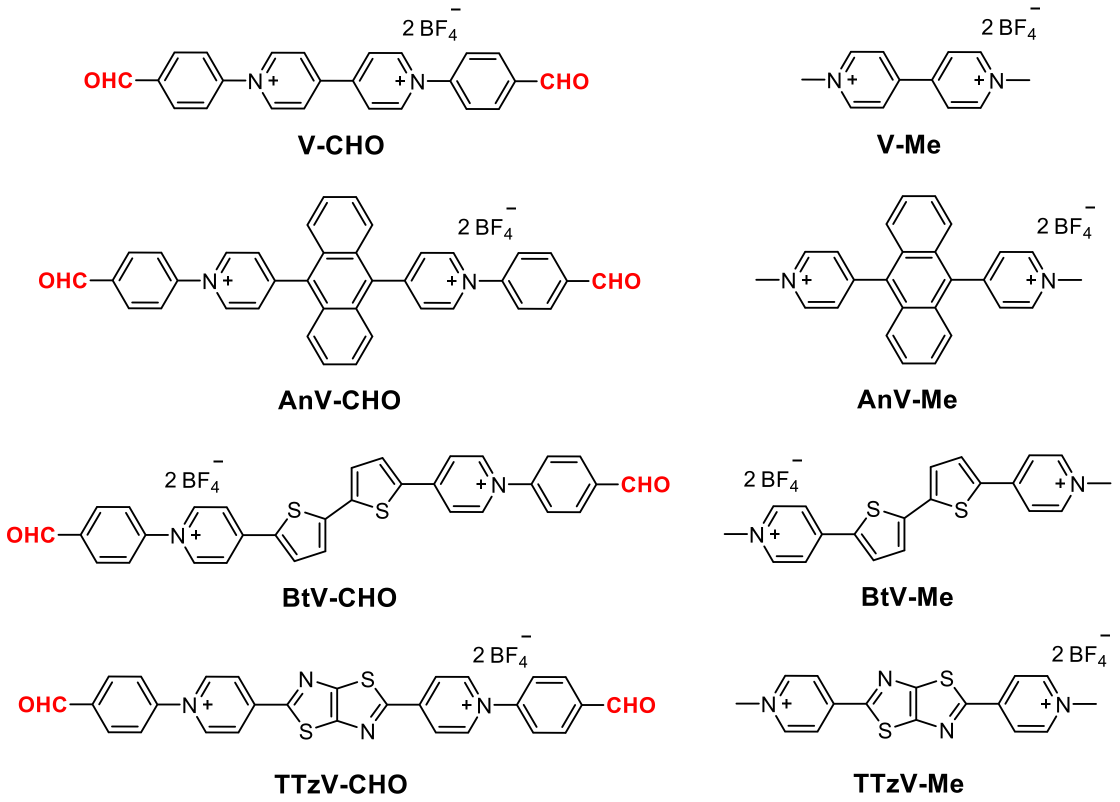

Figure S4. Chemical structures of **πV-CHO** and **πV-Me** in this study.

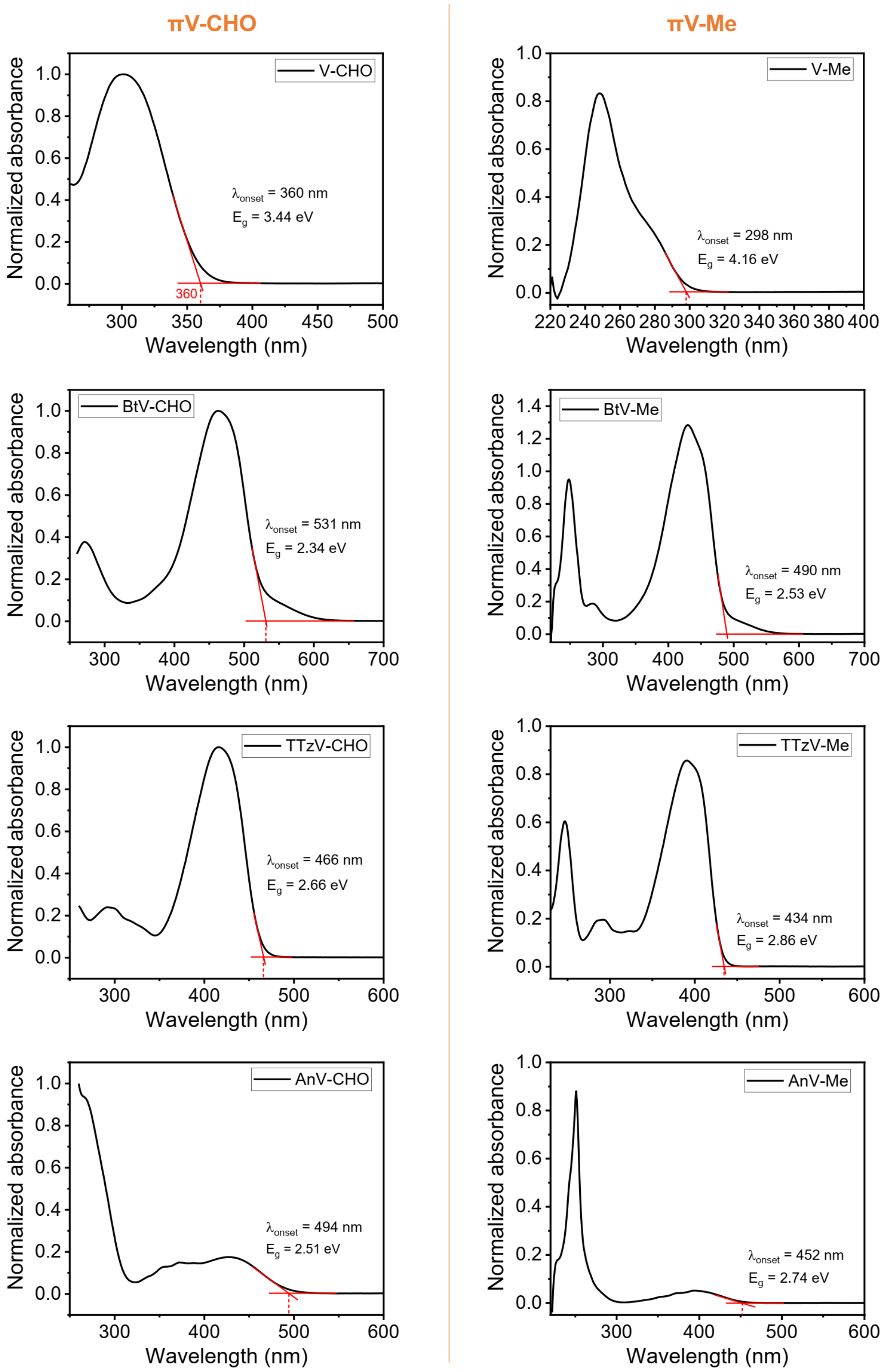

Figure S5. Calculation of the optical energy gap (E_g) from UV-vis spectra of (left) **πV-CHO** and (right) **πV-Me**. The UV-vis spectra were measured in CH_3CN solution.

The energy gap (E_g) can be evaluated from the onset of UV-Vis absorption spectrum (λ_{onset}) according to the formula: $E_g = (1240/\lambda_{onset})$ eV.

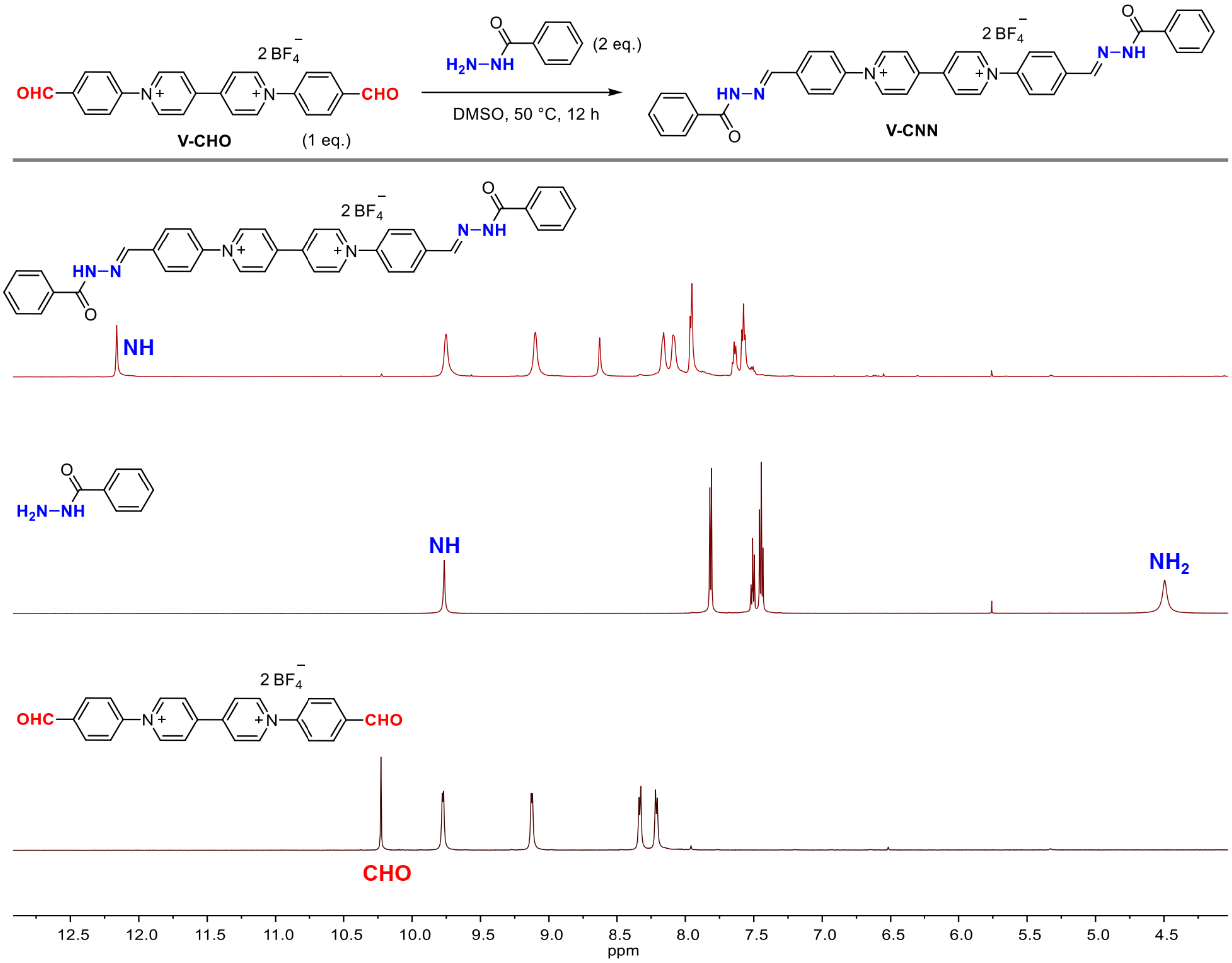

Figure S6. Monitoring the reactivity of **V-CHO** and benzohydrazide in d6-DMSO. Test condition: To a solution of **V-CHO** (2 mg/mL, 1 eq.) in d6-DMSO, was added benzohydrazide (2 eq.) in d6-DMSO. The mixture was maintained at 50 °C for 24 h.

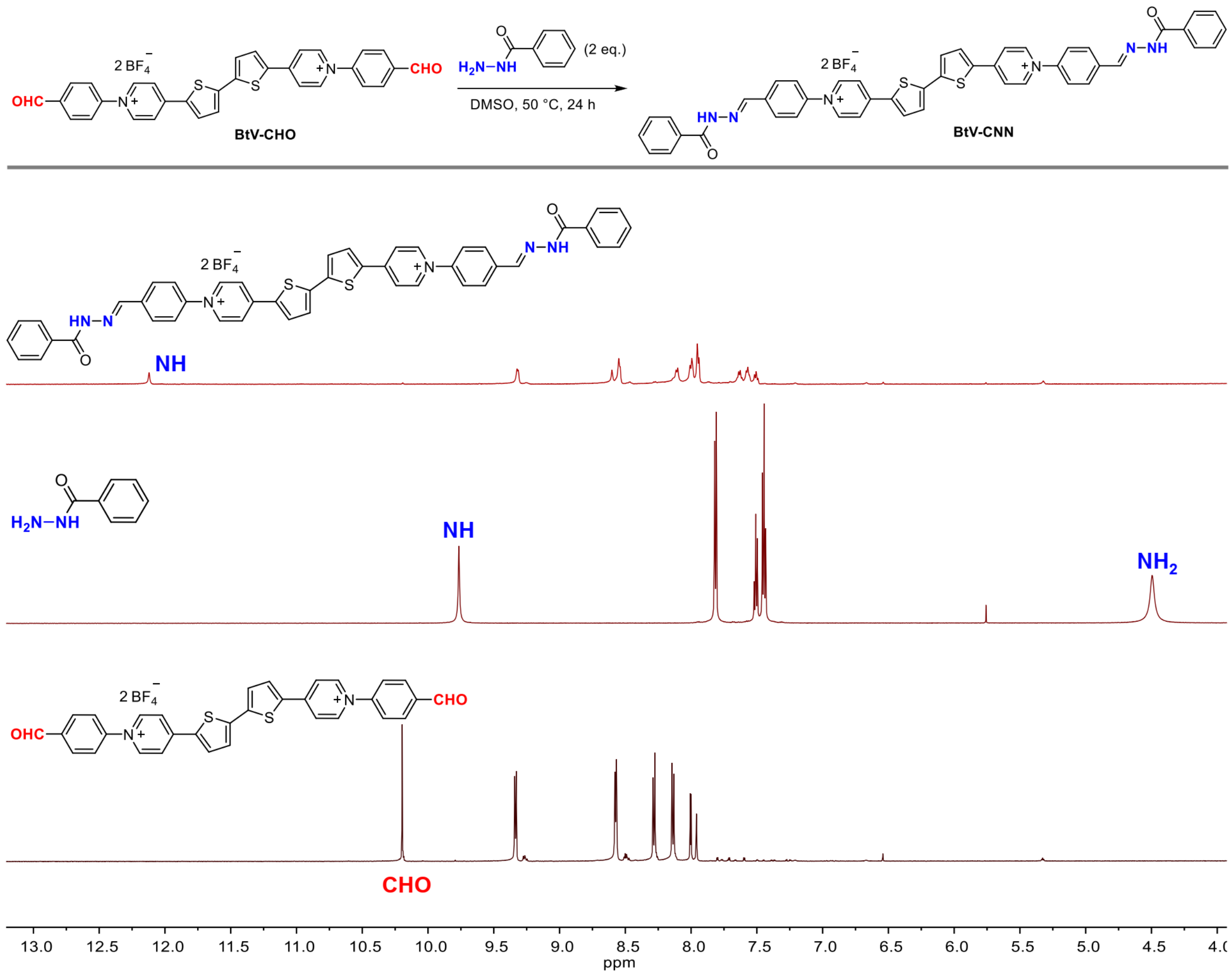

Figure S7. Monitoring the reactivity of **BtV-CHO** and benzohydrazide in d6-DMSO. Test condition: To a solution of **BtV-CHO** (2 mg/mL, 1 eq.) in d6-DMSO, was added benzohydrazide (2 eq.) in d6-DMSO. The mixture was maintained at 50 °C for 24 h.

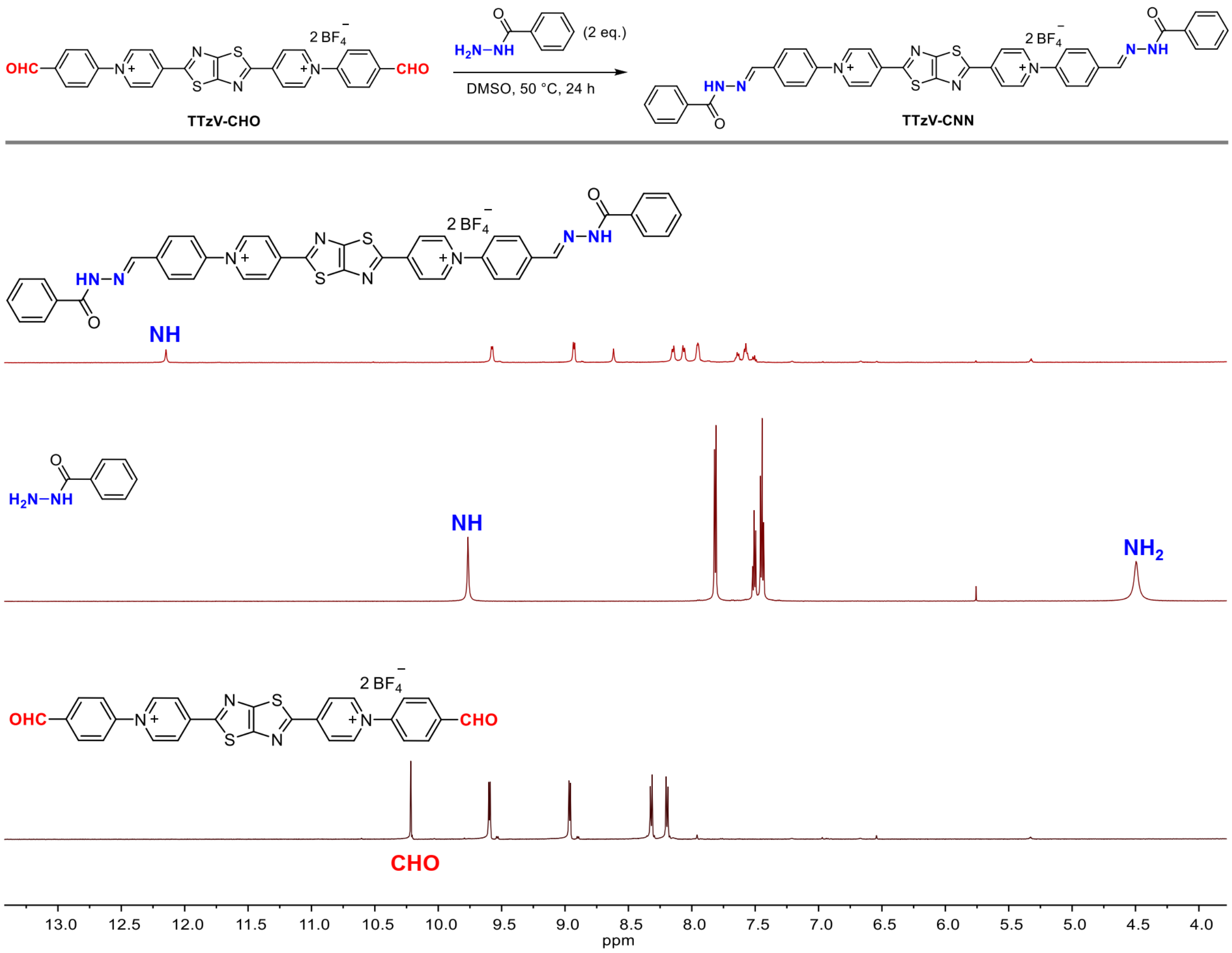

Figure S8. Monitoring the reactivity of **TTzV-CHO** and benzohydrazide in d6-DMSO. Test condition: To a solution of **TTzV-CHO** (2 mg/mL, 1 eq.) in d6-DMSO, was added benzohydrazide (2 eq.) in d6-DMSO. The mixture was maintained at 50 °C for 24 h.

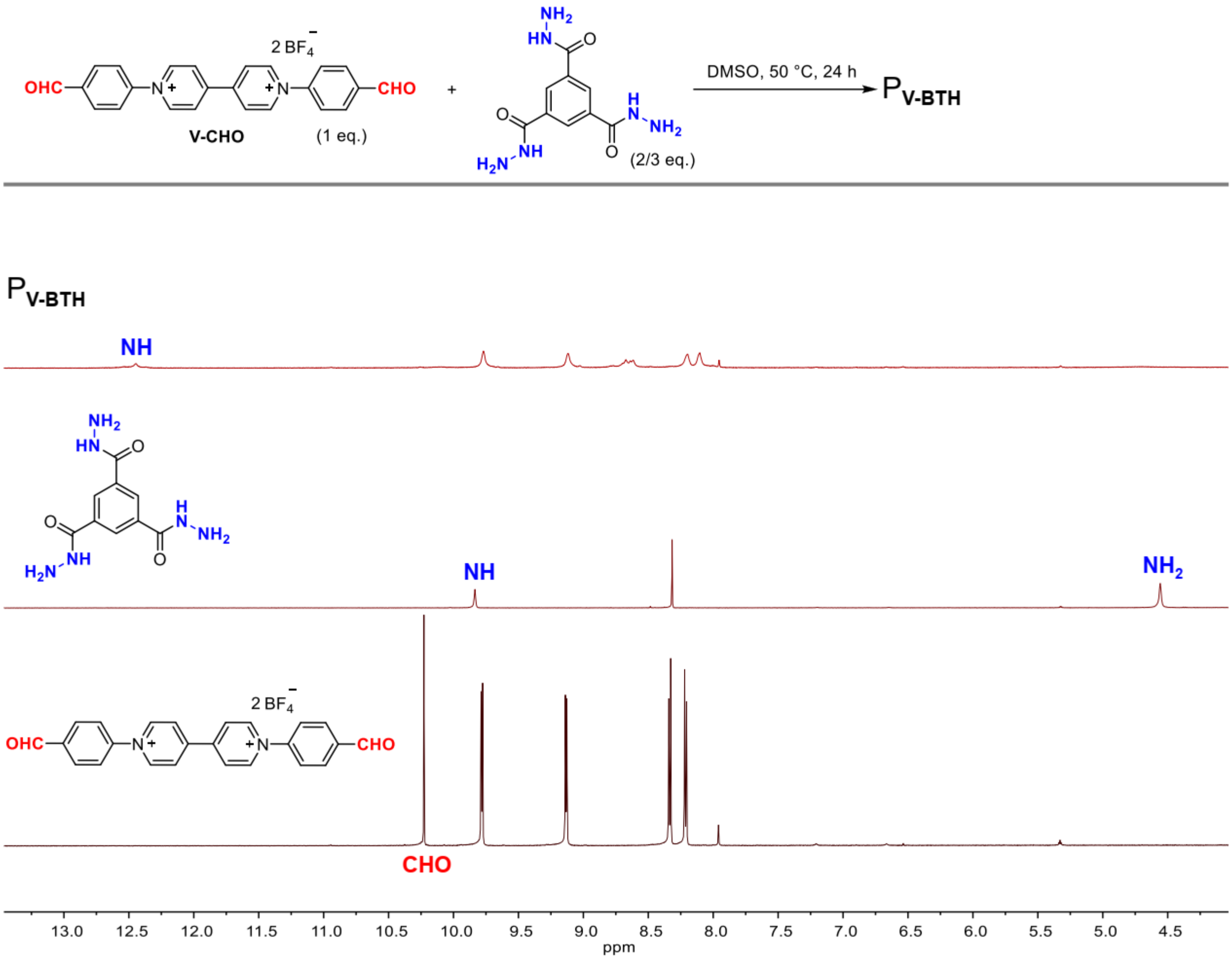

Figure S9. Monitoring the reactivity of **V-CHO** and benzene-1,3,5-tricarbohydrazide (**BTH**) in d6-DMSO. Test condition: To a solution of **V-CHO** (2 mg/mL, 1 eq.) in d6-DMSO, was added **BTH** (2/3 eq.) in d6-DMSO. The mixture was maintained at 50 °C for 24 h.

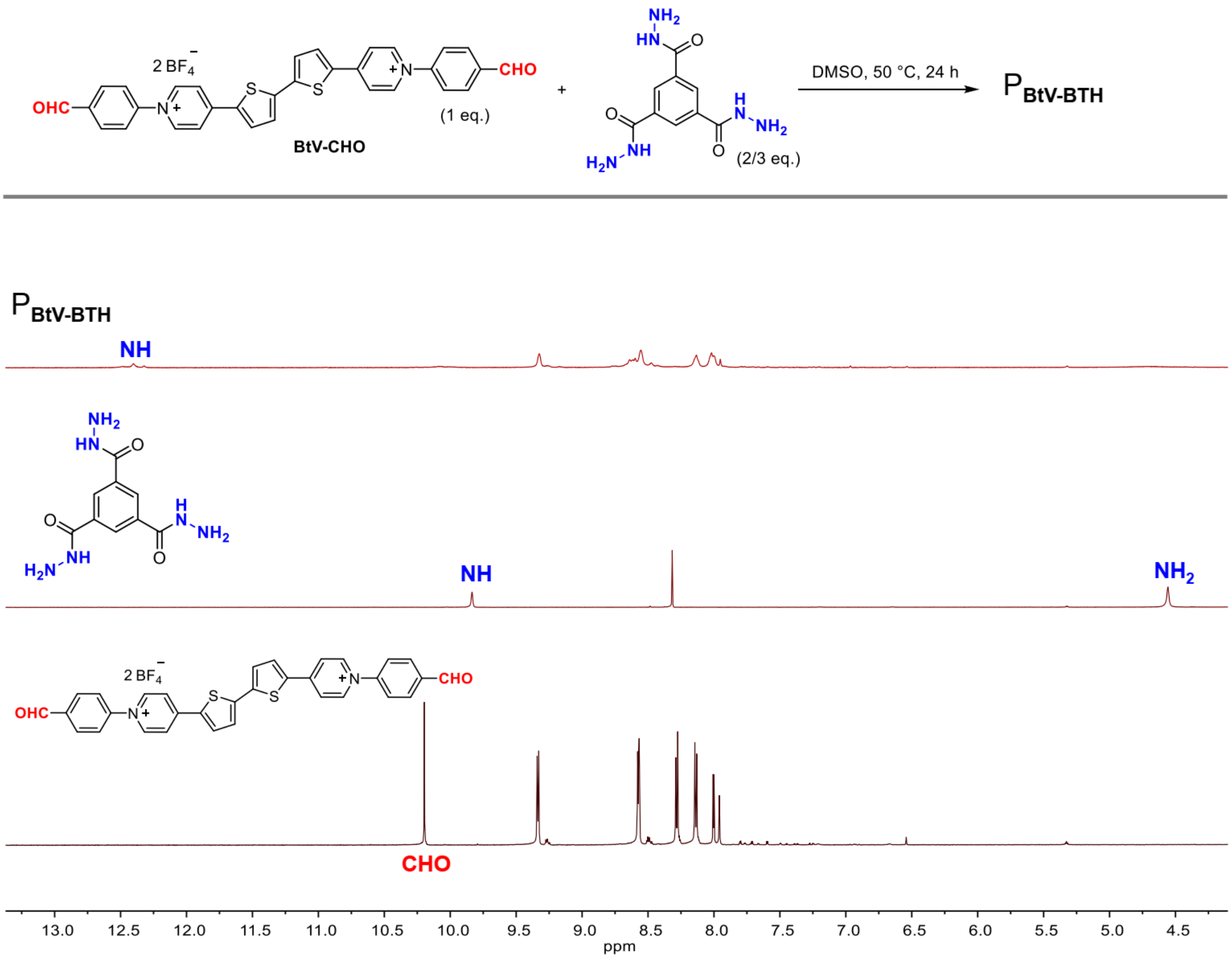

Figure S10. Monitoring the reactivity of **BtV-CHO** and benzene-1,3,5-tricarbohydrazide (**BTH**) in d6-DMSO. Test condition: To a solution of **BtV-CHO** (2 mg/mL, 1 eq.) in d6-DMSO, was added **BTH** (2/3 eq.) in d6-DMSO. The mixture was maintained at 50 °C for 24 h.

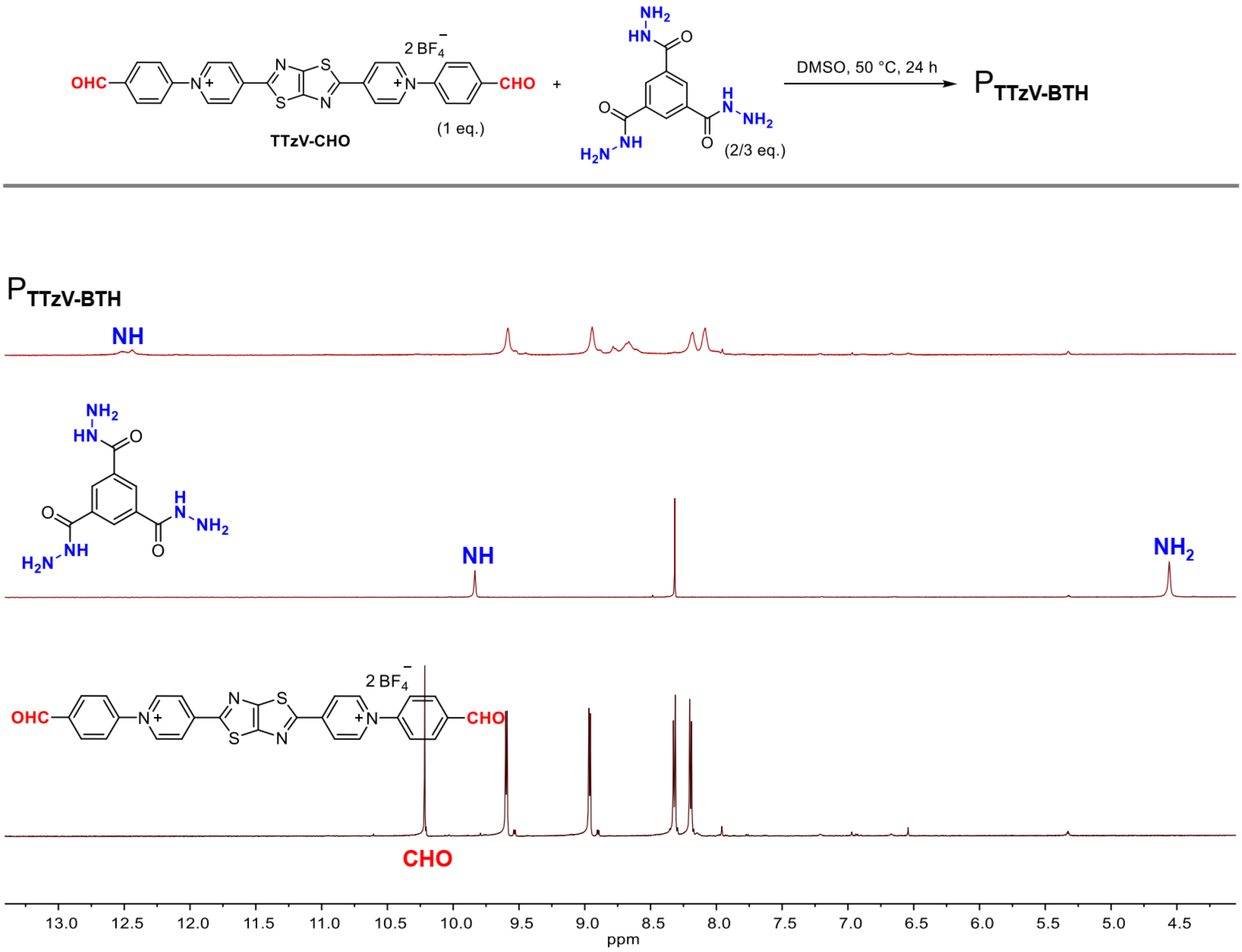

Figure S11. Monitoring the reactivity of **TTzV-CHO** and benzene-1,3,5-tricarbohydrazide (**BTH**) in d6-DMSO. Test condition: To a solution of **TTzV-CHO** (2 mg/mL, 1 eq.) in d6-DMSO, was added **BTH** (2/3 eq.) in d6-DMSO. The mixture was maintained at 50 °C for 24 h.

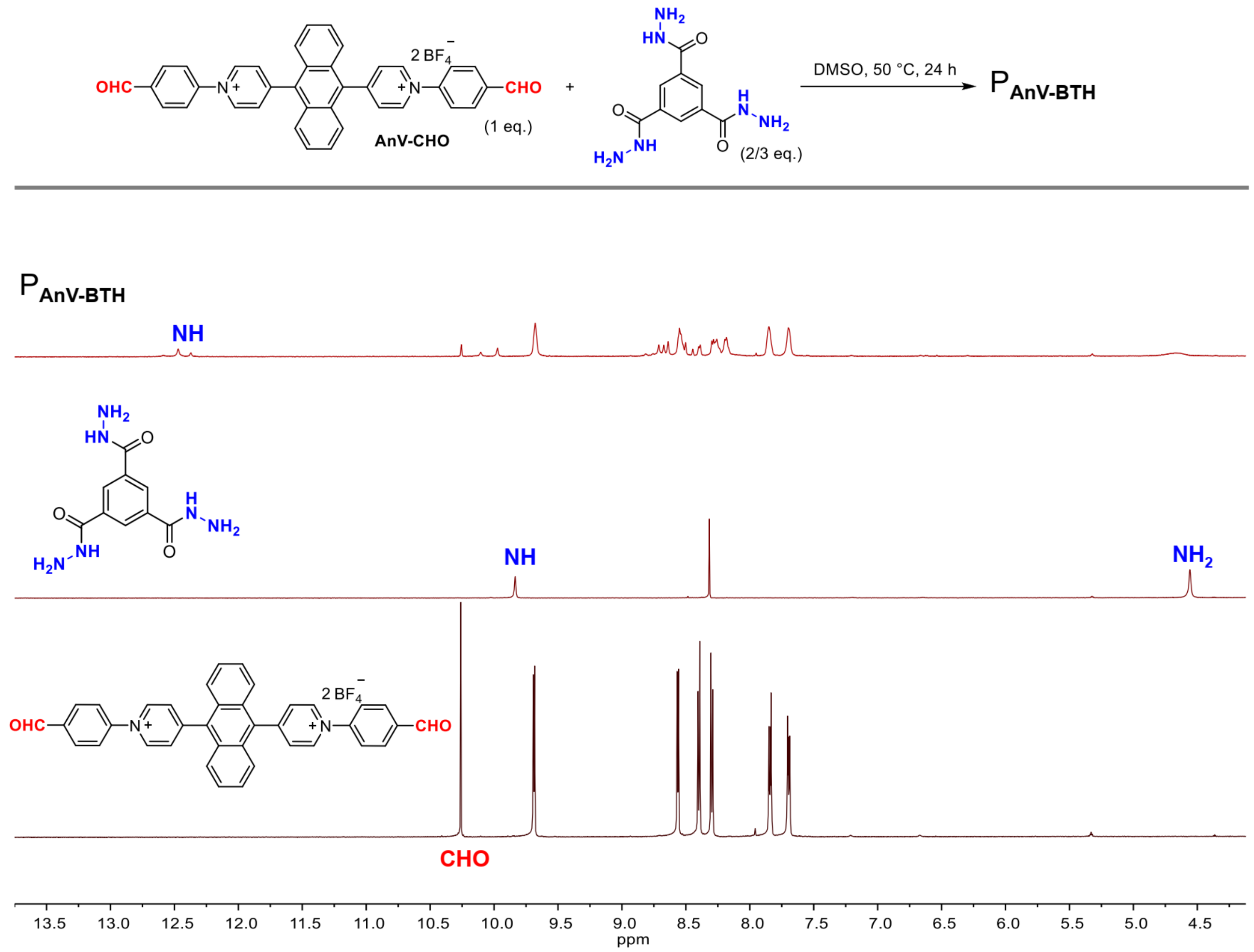

Figure S12. Monitoring the reactivity of **AnV-CHO** and benzene-1,3,5-tricarbohydrazide (**BTH**) in d6-DMSO. Test condition: To a solution of **AnV-CHO** (2 mg/mL, 1 eq.) in d6-DMSO, was added **BTH** (2/3 eq.) in d6-DMSO. The mixture was maintained at 50 °C for 24 h.

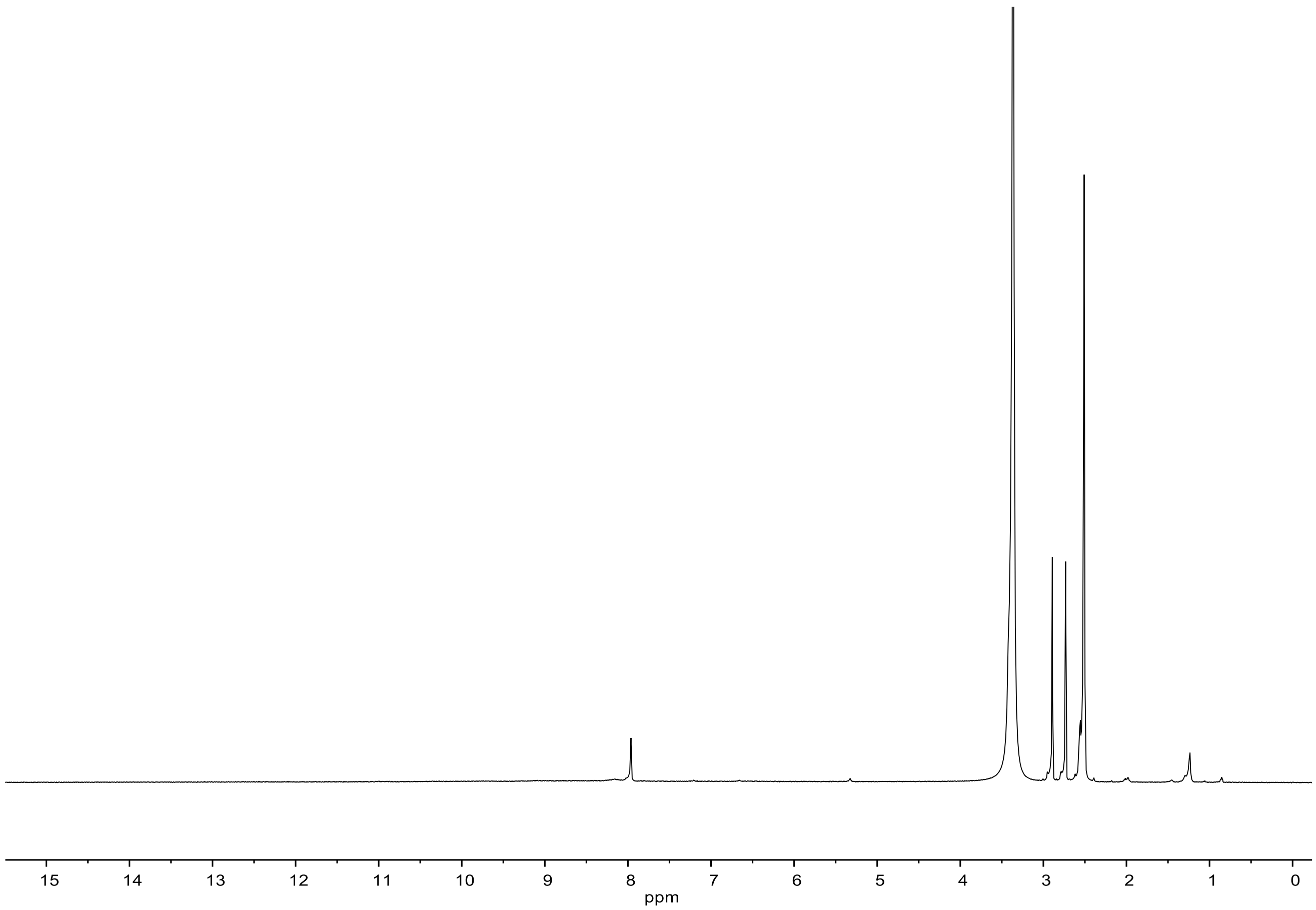

Figure S13. Monitoring the reactivity of **V-CHO** and benzene-1,3,5-tricarbohydrazide (**BTH**) in d6-DMSO. Test condition: To a solution of **V-CHO** (10 mg/mL, 1 eq.) in d6-DMSO, was added **BTH** (2/3 eq.) in d6-DMSO. The mixture was maintained at 50 °C for 24 h.

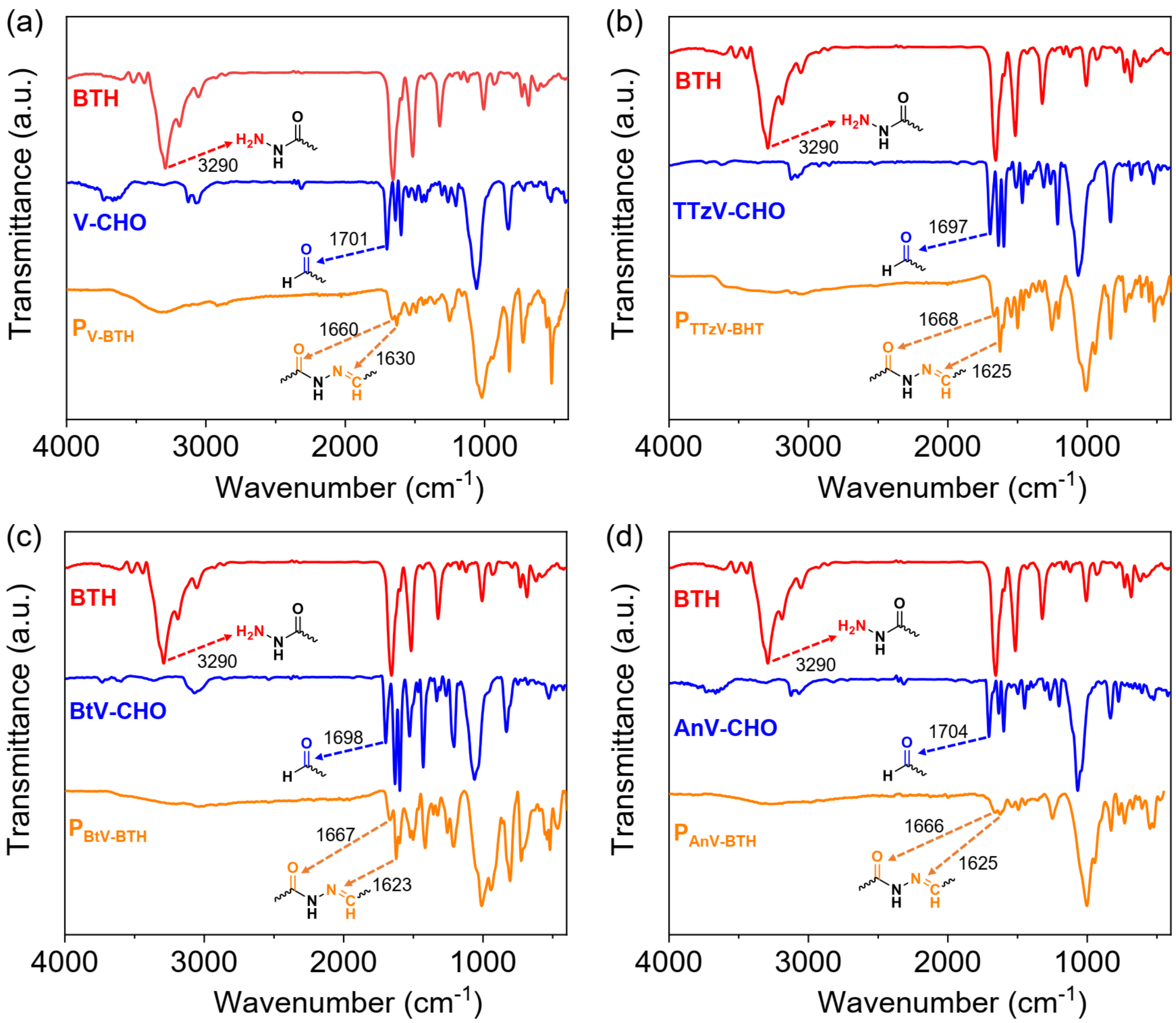

Figure S14. FT-IR spectra of (a) **BTH**, **V-CHO** and $\mathbf{P_{V\text{-}BTH}}$, (b) **BTH**, **TTzV-CHO** and $\mathbf{P_{TTzV\text{-}BTH}}$, (c) **BTH**, **BtV-CHO** and $\mathbf{P_{BtV\text{-}BTH}}$, and (d) **BTH**, **AnV-CHO** and $\mathbf{P_{AnV\text{-}BTH}}$.

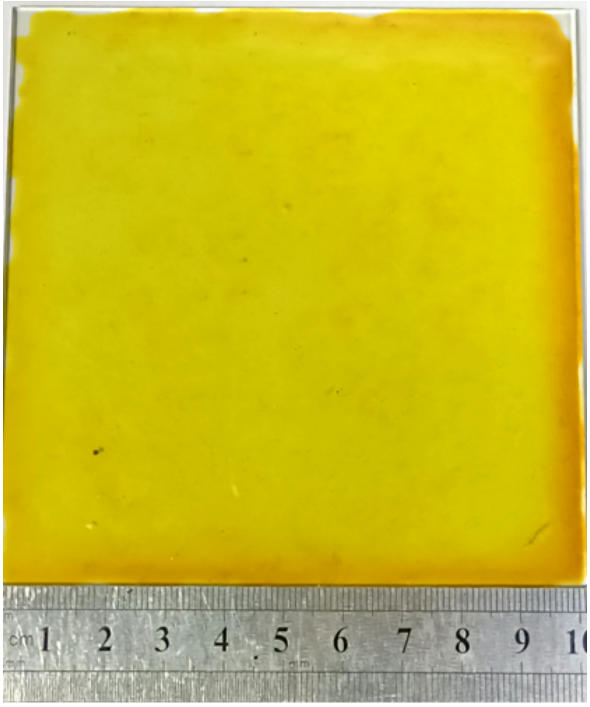

Figure S15. Large scale fabrication of the $\mathbf{P_{V\text{-}BTH}}$ thin film (10 cm × 10 cm) on glass.

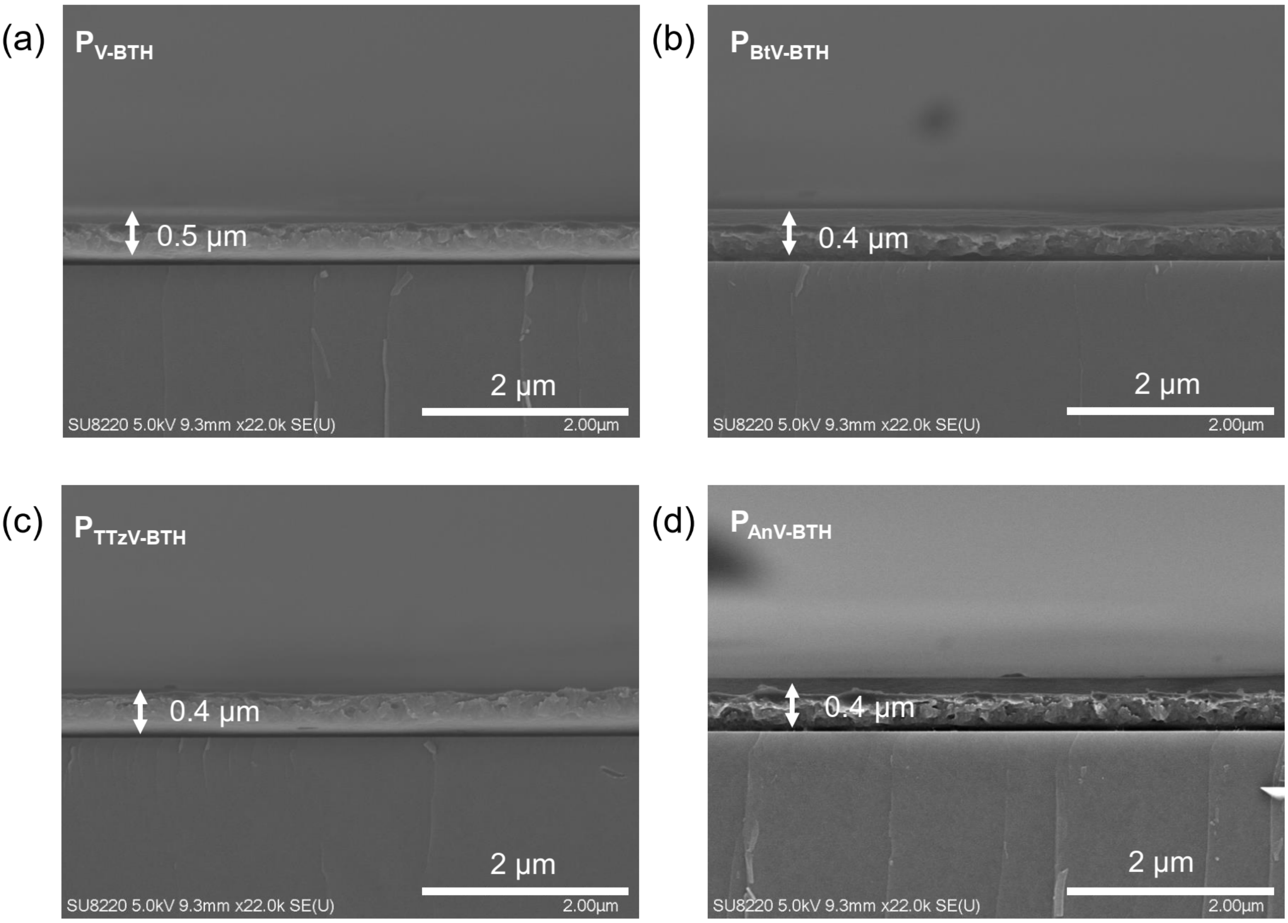

Figure S16. Cross-sectional SEM images of (a) **P$_{V\text{-}BTH}$**, (b) **P$_{BtV\text{-}BTH}$**, (c) **P$_{TTzV\text{-}BTH}$** and (d) **P$_{AnV\text{-}BTH}$** thin films on Si wafer. The areal mass weight was 0.1 mg cm^{-2}.

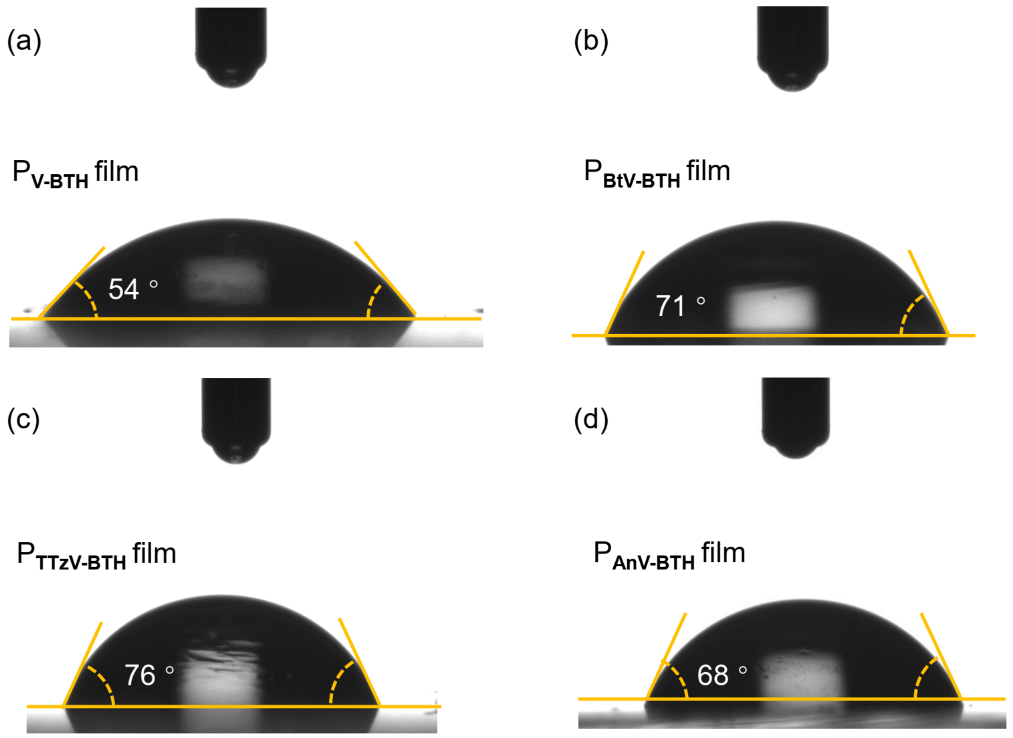

Figure S17. Photographs showing the water contact angles for the (a) **P$_{V\text{-}BTH}$**, (b) **P$_{BtV\text{-}BTH}$**, (c) **P$_{TTzV\text{-}BTH}$**, and (d) **P$_{AnV\text{-}BTH}$** films.

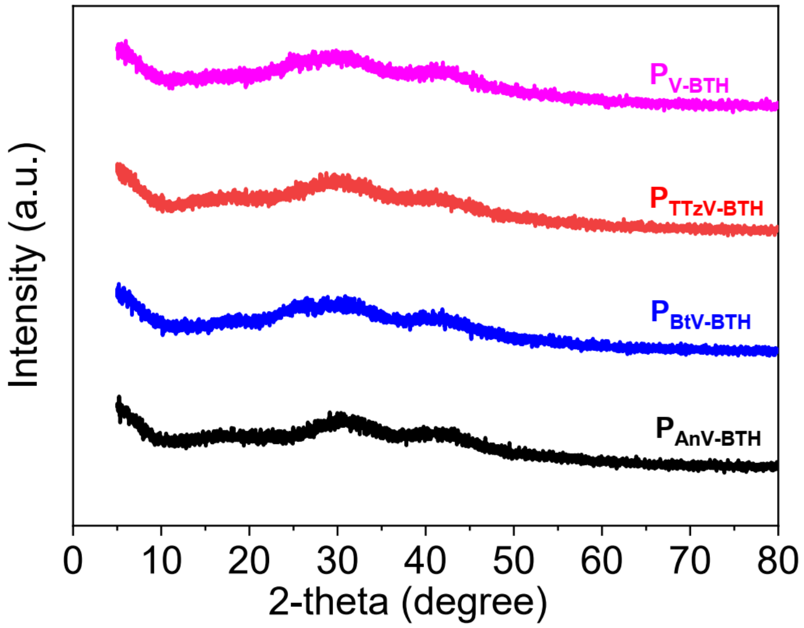

Figure S18. XRD of four **P$_{\pi V\text{-}BTH}$** films.

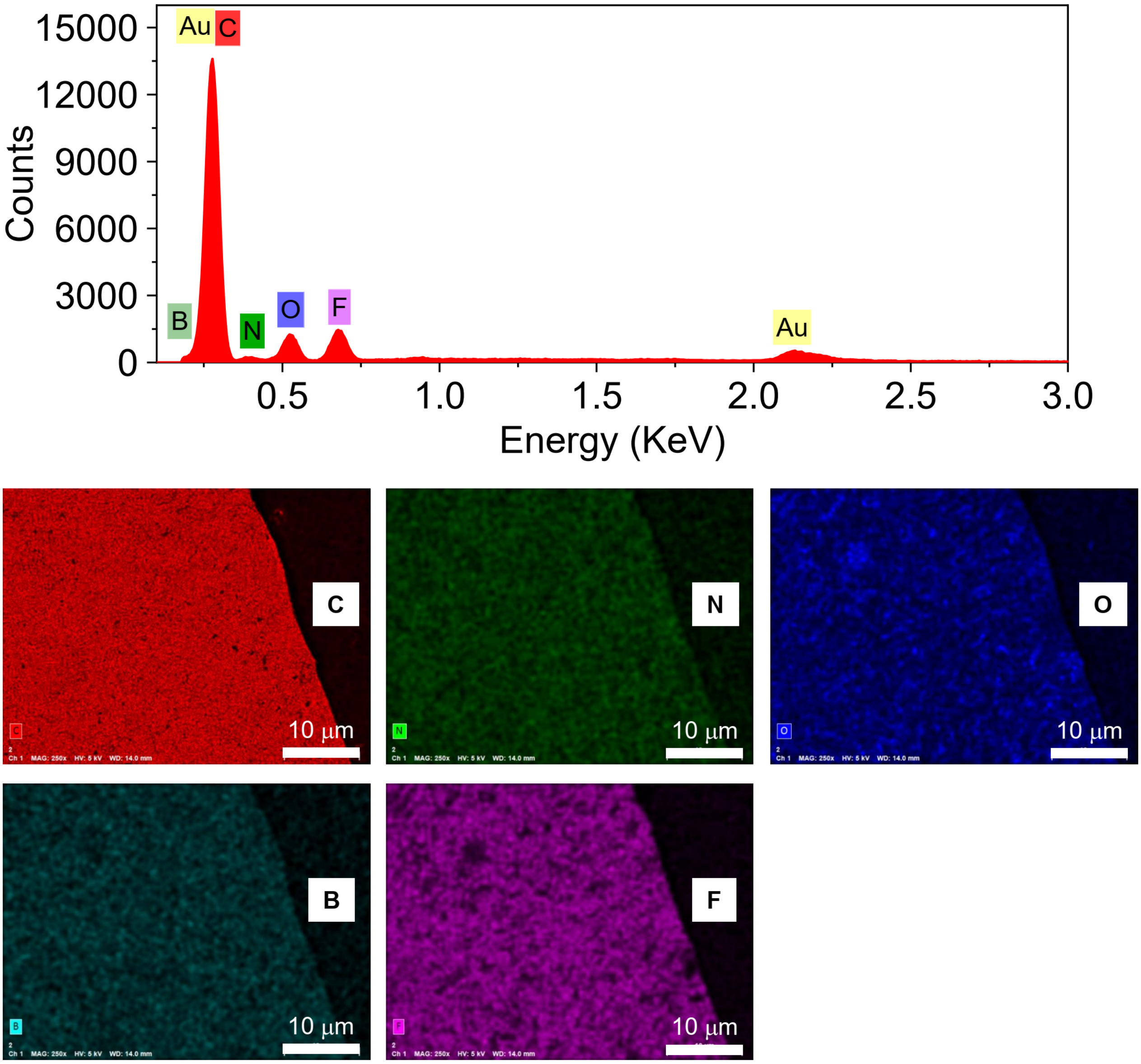

Figure S19. SEM image and EDX elemental mappings of $\mathbf{P_{V\text{-}BTH}}$ film.

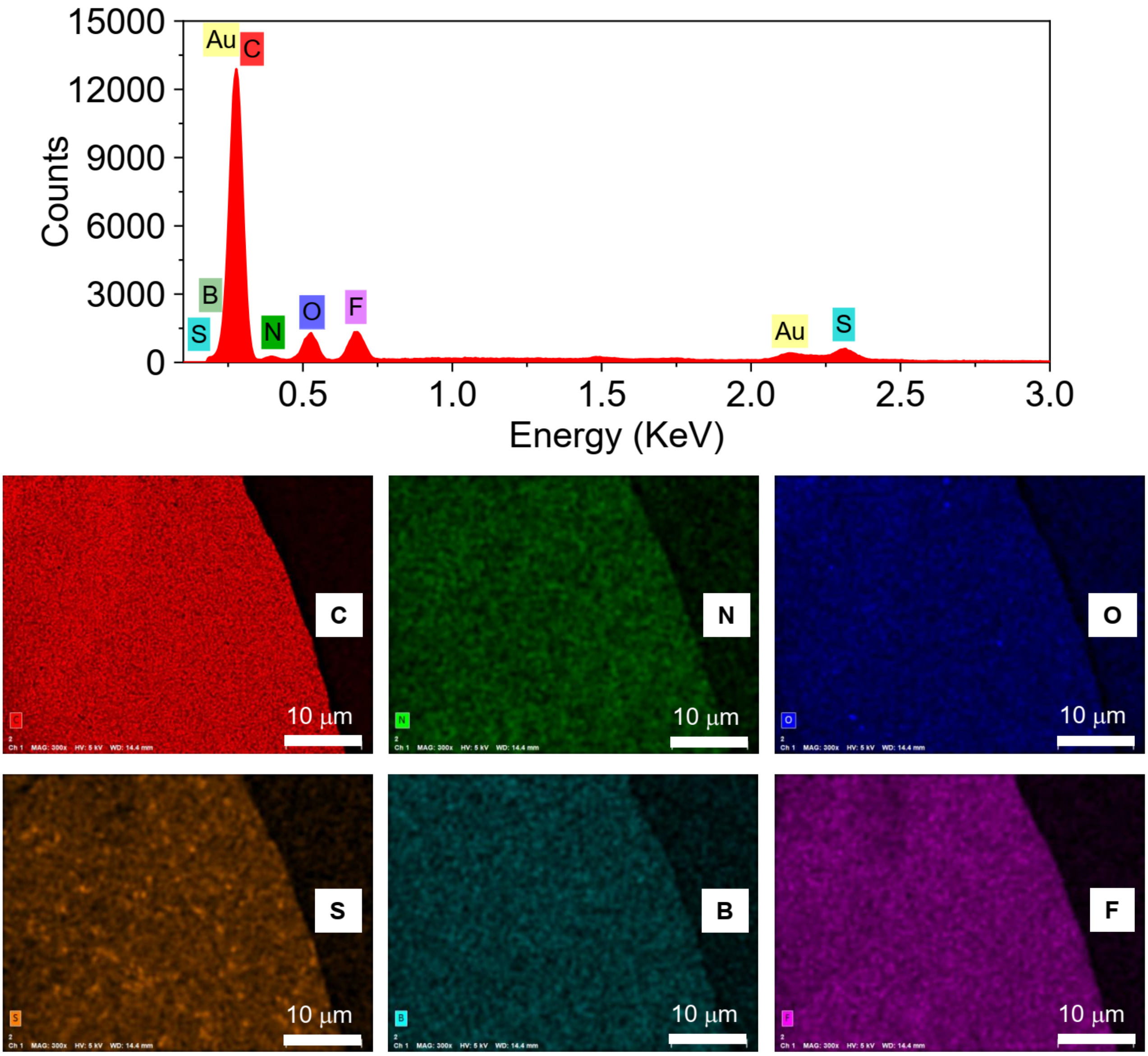

Figure S20. SEM image and EDX elemental mappings of $\mathbf{P_{BtV\text{-}BTH}}$ film.

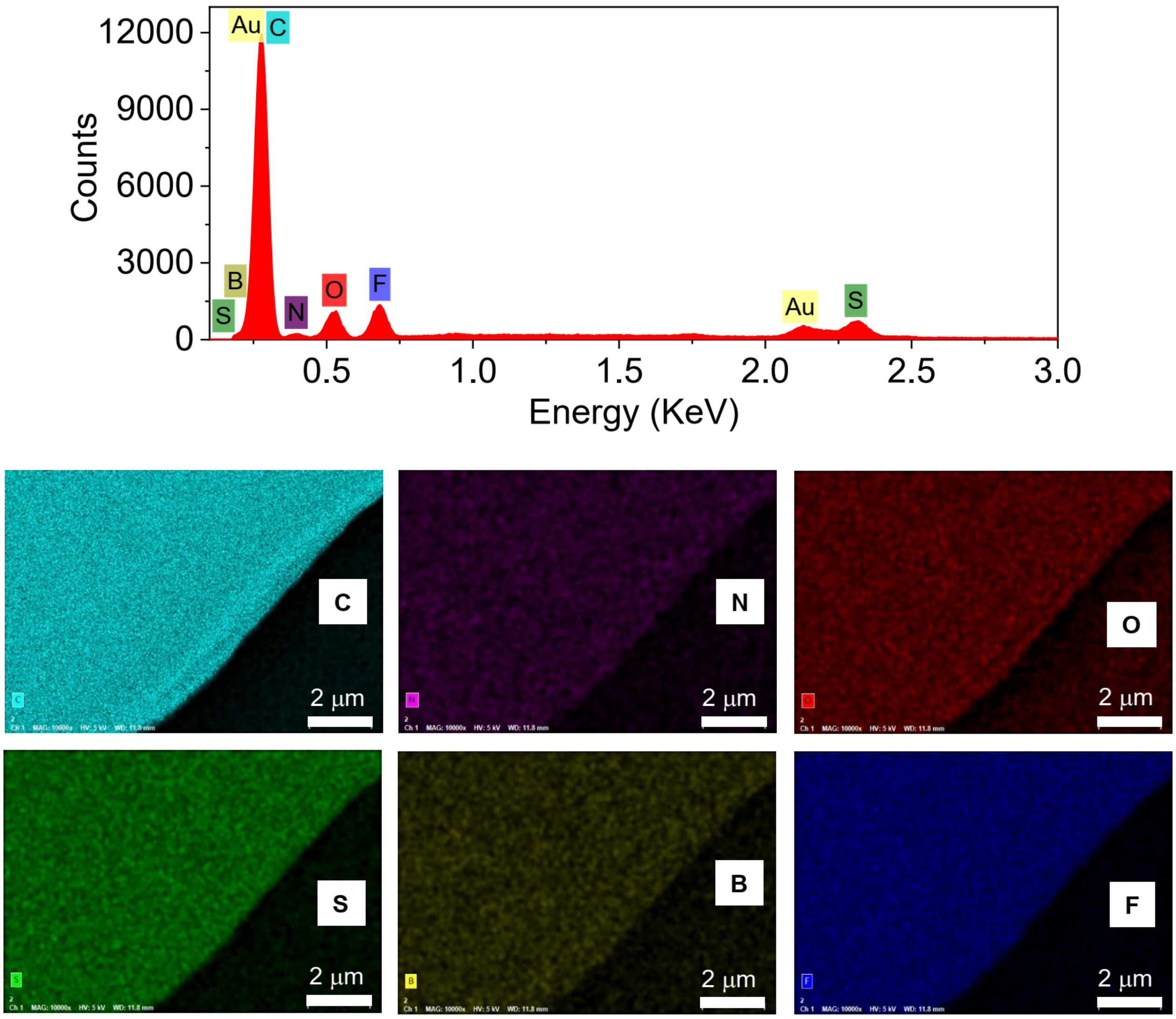

Figure S21. SEM image and EDX elemental mappings of $\mathbf{P_{TTzV\text{-}BTH}}$ film.

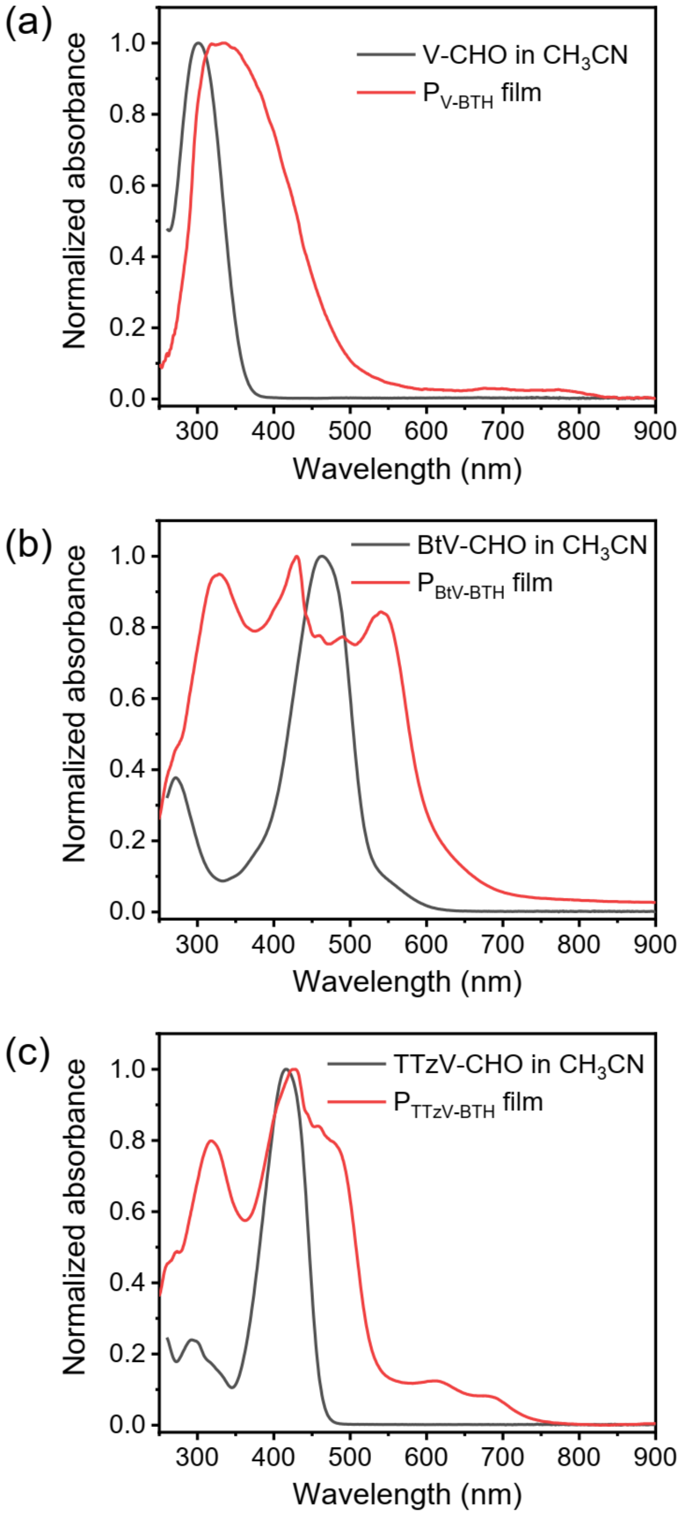

Figure S22. UV-vis comparison of **πV-CHO** in CH_3CN solution and $\mathbf{P_{\pi V\text{-}BTH}}$ films.

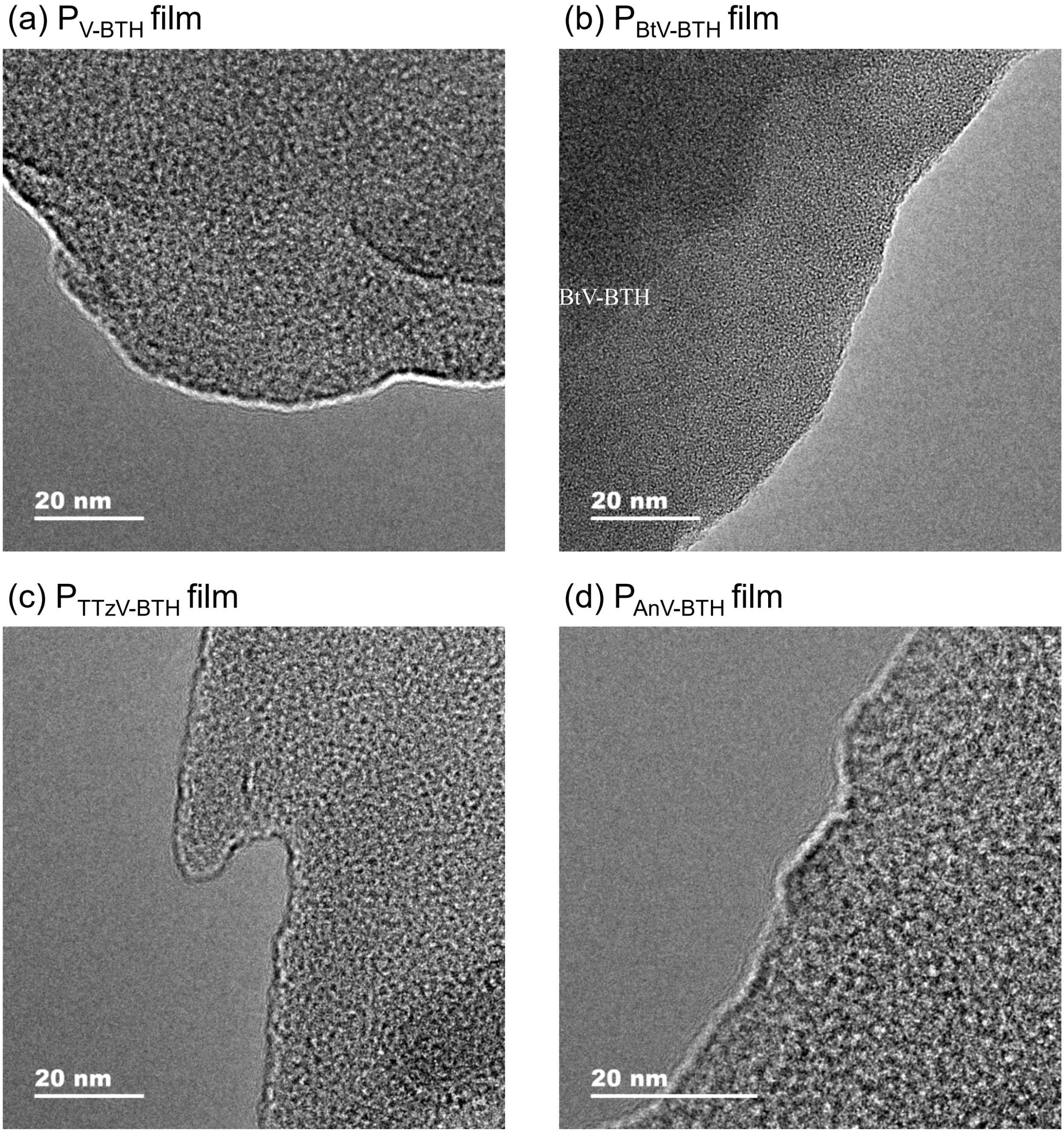

Figure S23. High resolution TEM images of (a) $\mathbf{P_{V\text{-}BTH}}$, (b) $\mathbf{P_{BtV\text{-}BTH}}$, (c) $\mathbf{P_{TTzV\text{-}BTH}}$ and (d) $\mathbf{P_{AnV\text{-}BTH}}$ films.

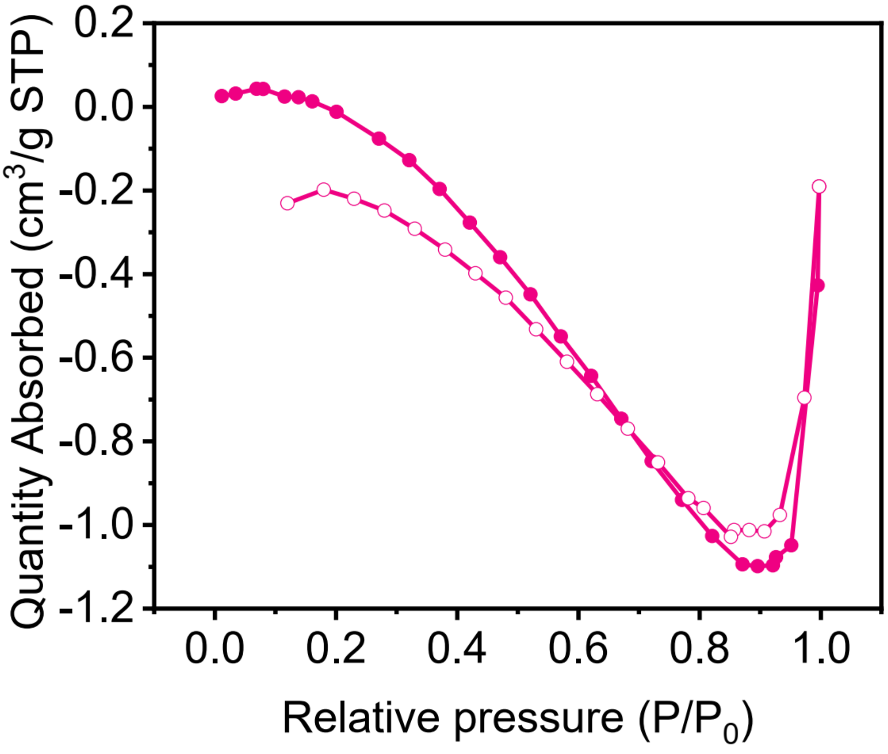

Figure S24. N_2 gas adsorption isotherms of $\mathbf{P_{V\text{-}BTH}}$ at 77 K.

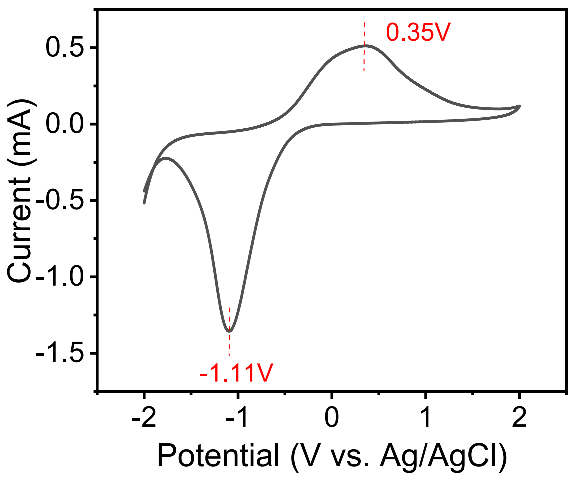

Figure S25. CV of $\mathbf{P_{V\text{-}BTH}}$ film in 0.1 M $LiClO_4$/propylene carbonate (PC) electrolyte solution.

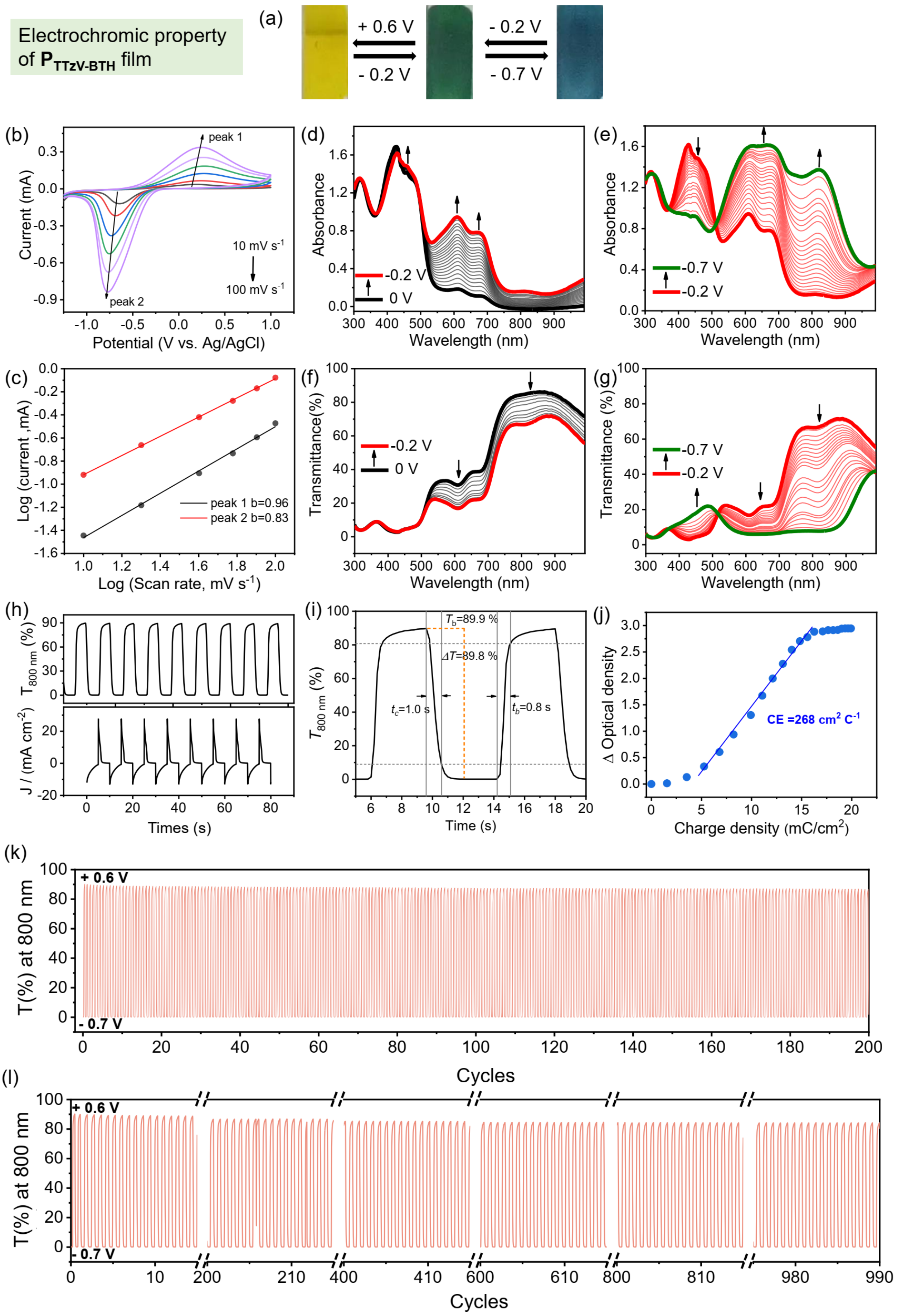

Electrochromic property of $P_{TTzV-BTH}$ film
(a)
+ 0.6 V
- 0.2 V
- 0.2 V
- 0.7 V
(b)
peak 1
peak 2
10 mV s-1
100 mV s-1
Current (mA)
Potential (V vs. Ag/AgCl)
(c)
Log (current ,mA)
Log (Scan rate, mV s-1)
peak 1 b=0.96
peak 2 b=0.83
(d)
Absorbance
Wavelength (nm)
-0.2 V
0 V
(e)
Absorbance
Wavelength (nm)
-0.7 V
-0.2 V
(f)
Transmittance(%)
Wavelength (nm)
-0.2 V
0 V
(g)
Transmittance (%)
Wavelength (nm)
-0.7 V
-0.2 V
(h)
T800 nm (%)
J / (mA cm-2)
Times (s)
(i)
T800 nm (%)
Time (s)
Tb=89.9 %
ΔT=89.8 %
tc=1.0 s
tb=0.8 s
(j)
Δ Optical density
Charge density (mC/cm2)
CE =268 cm2 C-1
(k)
T(%) at 800 nm
Cycles
+ 0.6 V
- 0.7 V
(l)
T(%) at 800 nm
Cycles
+ 0.6 V
- 0.7 V

Figure S26. Electrochromic properties of the $\mathbf{P_{TTzV\text{-}BTH}}$ film. (a) Reversible color changes of the $\mathbf{P_{TTzV\text{-}BTH}}$ film during reduction and oxidation. (b) CV curves of the $\mathbf{P_{TTzV\text{-}BTH}}$ film measured in 0.1 M LiCl aqueous electrolyte at scan rates of 10, 20, 40, 60, 80, and 100 mV s^{-1}. (c) Log *i* versus log *v* plots to determine the *b* values of different peaks. (d-e) UV–vis spectra changes of $\mathbf{P_{TTzV\text{-}BTH}}$ film in 0.1 M LiCl aqueous electrolyte recorded with applied voltage of (d) 0 V to -0.2 V and (e) -0.2 to -0.7 V (V vs Ag/AgCl). (f-g) Transmission spectra changes of $\mathbf{P_{TTzV\text{-}BTH}}$ film in 0.1 M LiCl aqueous electrolyte recorded with applied voltage of (d) 0 V to -0.2 V and (e) -0.2 V to -0.7 V (V vs Ag/AgCl). (h) Plots of transmittance at 800 nm and current versus time with alternating voltage of -0.7 V and +0.6 V. (i) Coloration/bleaching transmission spectrum of $\mathbf{P_{TTzV\text{-}BTH}}$ film at 800 nm with an applied voltage of -0.7 V and +0.6 V. (j) Plots of the optical density versus charge density and the slope as coloration efficiency. (k-l) Cycling stability of $\mathbf{P_{TTzV\text{-}BTH}}$ film between -0.7 V and +0.6 V. The first 200 cycles recorded at 800 nm transmittance are plotted in (k), and the total 1000 cycles are shown in (l).

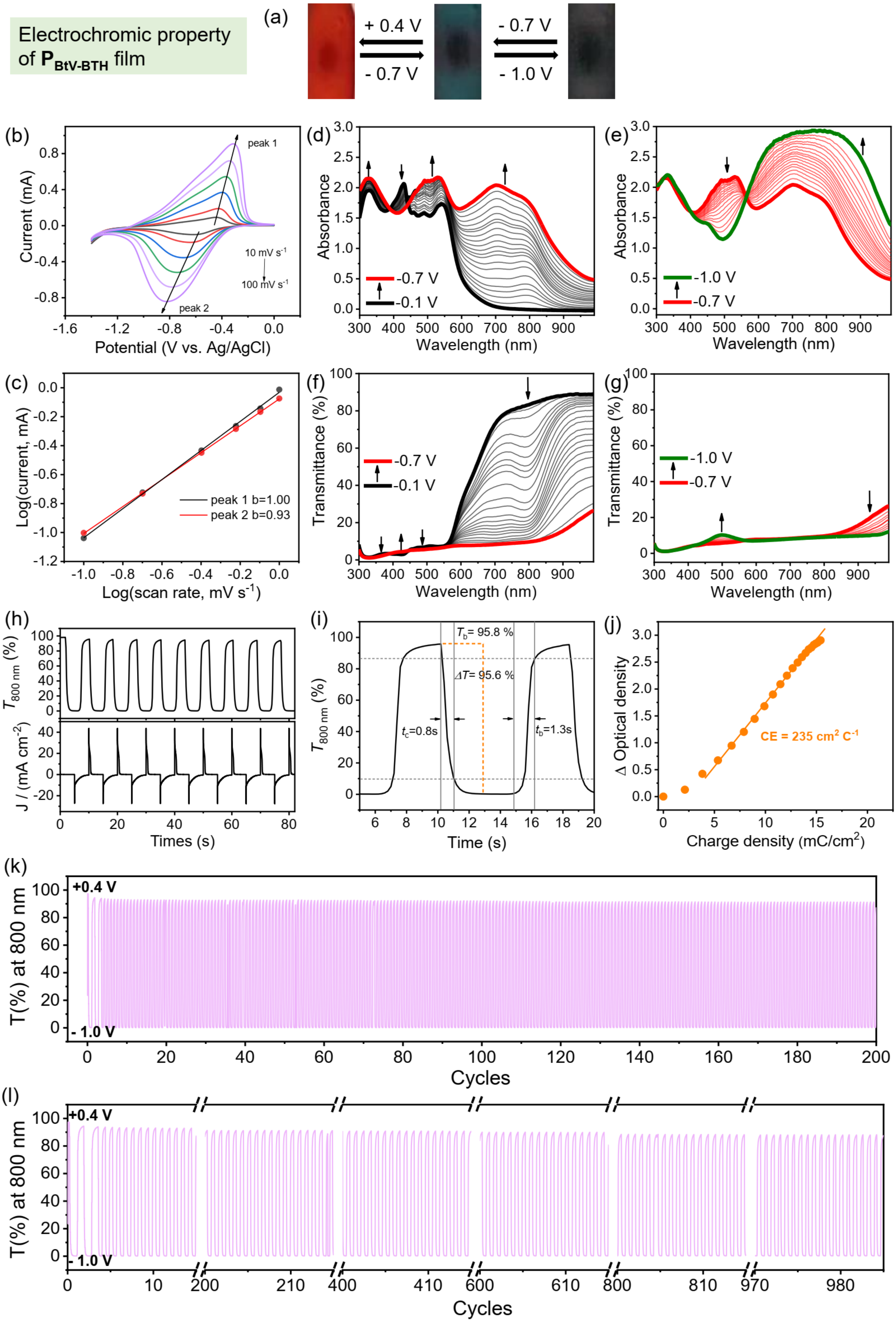
Electrochromic property of $P_{BtV-BTH}$ film
(a)
+ 0.4 V
- 0.7 V
- 0.7 V
- 1.0 V
(b)
peak 1
peak 2
10 mV s-1
100 mV s-1
Current (mA)
Potential (V vs. Ag/AgCl)
(c)
Log(current, mA)
Log(scan rate, mV s-1)
peak 1 b=1.00
peak 2 b=0.93
(d)
Absorbance
Wavelength (nm)
-0.7 V
-0.1 V
(e)
Absorbance
Wavelength (nm)
-1.0 V
-0.7 V
(f)
Transmittance (%)
Wavelength (nm)
-0.7 V
-0.1 V
(g)
Transmittance (%)
Wavelength (nm)
-1.0 V
-0.7 V
(h)
T800 nm (%)
J / (mA cm-2)
Times (s)
(i)
Tb= 95.8 %
ΔT= 95.6 %
tc=0.8s
tb=1.3s
T800 nm (%)
Time (s)
(j)
Δ Optical density
CE = 235 cm2 C-1
Charge density (mC/cm2)
(k)
+0.4 V
- 1.0 V
T(%) at 800 nm
Cycles
(l)
+0.4 V
- 1.0 V
T(%) at 800 nm
Cycles

Figure S27. Electrochromic properties of the $\mathbf{P_{BtV\text{-}BTH}}$ film. (a) Reversible color changes of the $\mathbf{P_{BtV\text{-}BTH}}$ film during reduction and oxidation. (b) CV curves of the $\mathbf{P_{BtV\text{-}BTH}}$ film measured in 0.1 M LiCl aqueous electrolyte at scan rates of 10, 20, 40, 60, 80, and 100 mV s^{-1}. (c) Log i versus log v plots to determine the b values of different peaks. (d-e) UV–vis spectra changes of $\mathbf{P_{BtV\text{-}BTH}}$ film in 0.1 M LiCl aqueous electrolyte recorded with applied voltage of (d) -0.1 V to -0.7 V and (e) -0.7 V to -1.0 V (V vs Ag/AgCl). (f-g) Transmission spectra changes of $\mathbf{P_{BtV\text{-}BTH}}$ film in 0.1 M LiCl aqueous electrolyte recorded with applied voltage of (d) -0.1 V to -0.7 V and (e) -0.7 V to -1.0 V (V vs Ag/AgCl). (h) Plots of transmittance at 800 nm and current versus time with alternating voltage of -1.0 V and +0.4 V. (i) Coloration/bleaching transmission spectrum of $\mathbf{P_{BtV\text{-}BTH}}$ film at 800 nm with an applied voltage of -1.0 V and +0.4 V. (j) Plots of the optical density versus charge density and the slope as coloration efficiency. (k-l) Cycling stability of $\mathbf{P_{BtV\text{-}BTH}}$ film between -1.0 V and +0.4 V. The first 200 cycles recorded at 800 nm transmittance are plotted in (k), and the total 1000 cycles are shown in (l).

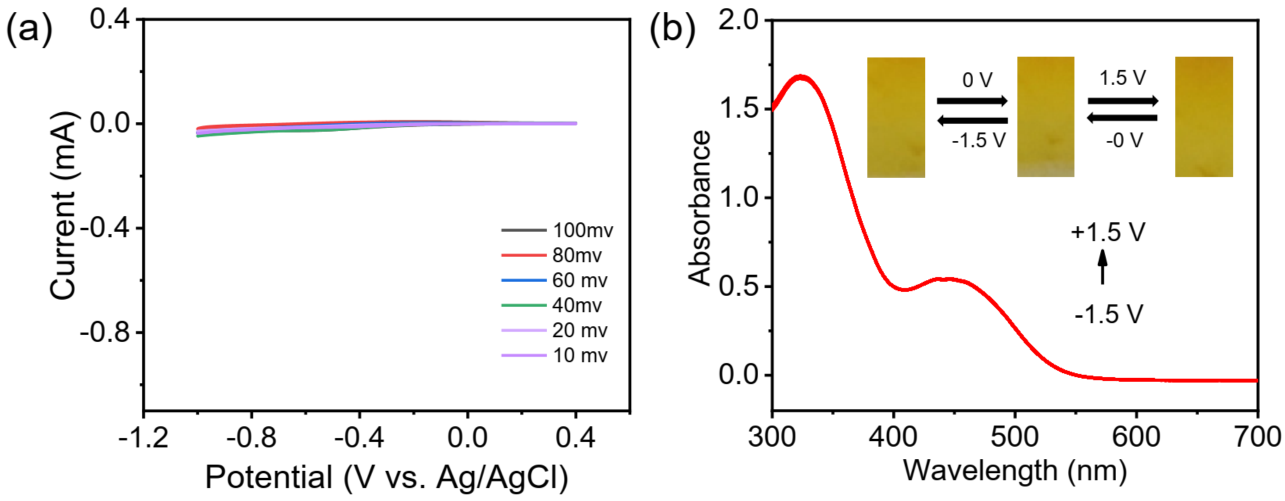

Figure S28. (a) CV curves of the $\mathbf{P_{AnV\text{-}BTH}}$ film measured in 0.1 M LiCl aqueous electrolyte at scan rates of 10, 20, 40, 60, 80, and 100 mV s^{-1}. (b) UV–vis spectra changes of $\mathbf{P_{AnV\text{-}BTH}}$ film in 0.1 M LiCl aqueous electrolyte recorded with applied voltage of -1.5 V to +1.5 V vs Ag/AgCl).

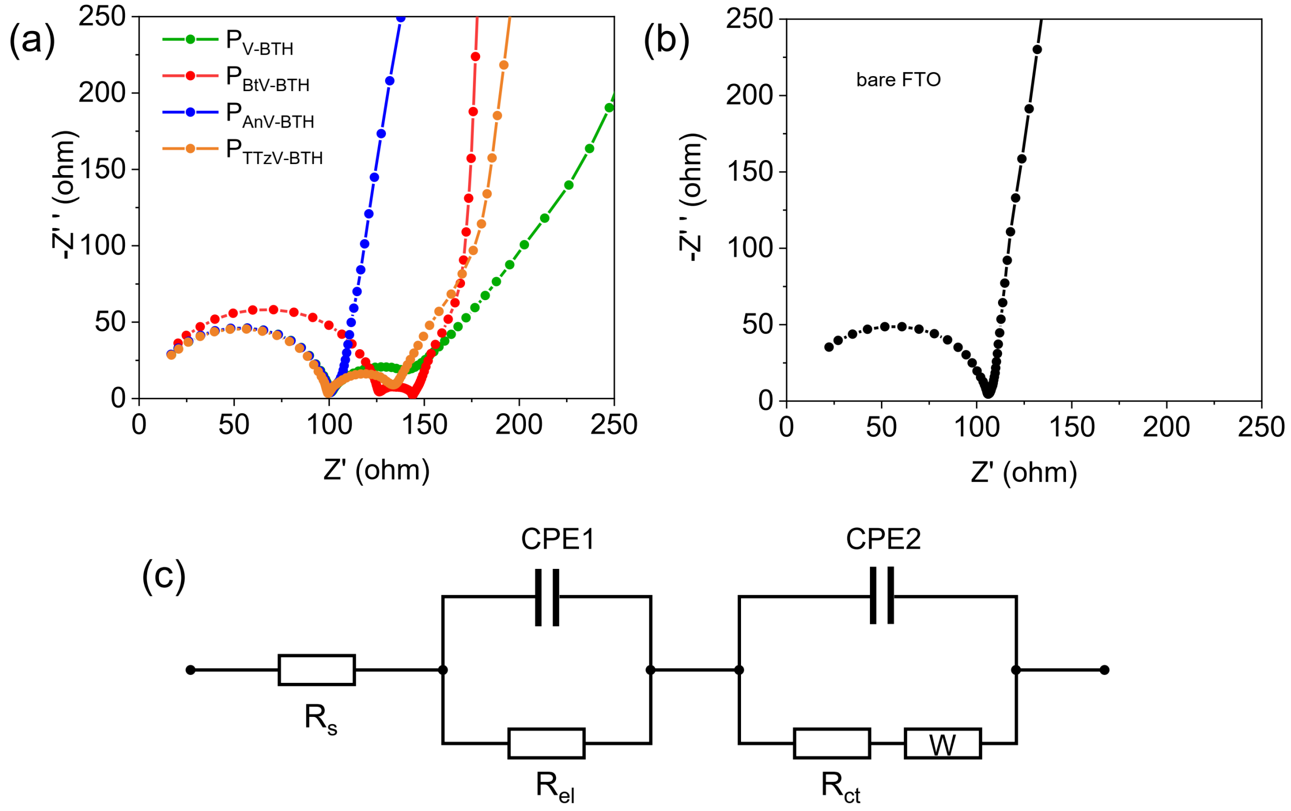

Figure S29. Electrochemical impedance spectrum (EIS) of (a) $\mathbf{P_{\pi V\text{-}BTH}}$ films coated FTO and (b) bare FTO in the frequency range of 10^6 Hz to 0.01 Hz. (c) Equivalent circuit of $\mathbf{P_{\pi V\text{-}BTH}}$ films.

As shown in Figure S28a, $\mathbf{P_{V\text{-}BTH}}$, $\mathbf{P_{TTzV\text{-}BTH}}$ and $\mathbf{P_{BtV\text{-}BTH}}$ films coated FTO have two semicircle-regions in the Nyquist curves (left higher frequency region and right-lower frequency region). The high frequency semicircle comes from the capacitance of electrode, which accounts for charge accumulation at the interface of the FTO electrode and the electrolyte solution interface. This is confirmed by the control experiment, with an FTO electrode without EC film. As shown in Figure S28b, the bare FTO electrode only showed one semicircle in the high region, with a resistance (R_{el}) of ca. 100 Ω. The second semicircle at intermediate frequencies is ascribed to electron transfer across the interface between the EC films and the electrode. The frequency intercept on the real axis represents the charge-transfer resistance (R_{ct}) at the film-electrode interface. The R_{ct} values for $\mathbf{P_{V\text{-}BTH}}$, $\mathbf{P_{TTzV\text{-}BTH}}$ and $\mathbf{P_{BtV\text{-}BTH}}$ films are determined to be 43, 35 and 20 Ω. Compared to $\mathbf{P_{V\text{-}BTH}}$ film, the smaller R_{ct} values for $\mathbf{P_{TTzV\text{-}BTH}}$ and $\mathbf{P_{BtV\text{-}BTH}}$ films indicate improved electronic conductivity, which was tentatively attributed to the larger conjugated viologen skeletons. In contrast, $\mathbf{P_{AnV\text{-}BTH}}$ film did not show a second semicircle region, probably because of its poor

electron conductivity, which is consistent with its poorest EC performance. The equivalent circuit is presented in Figure S28c.

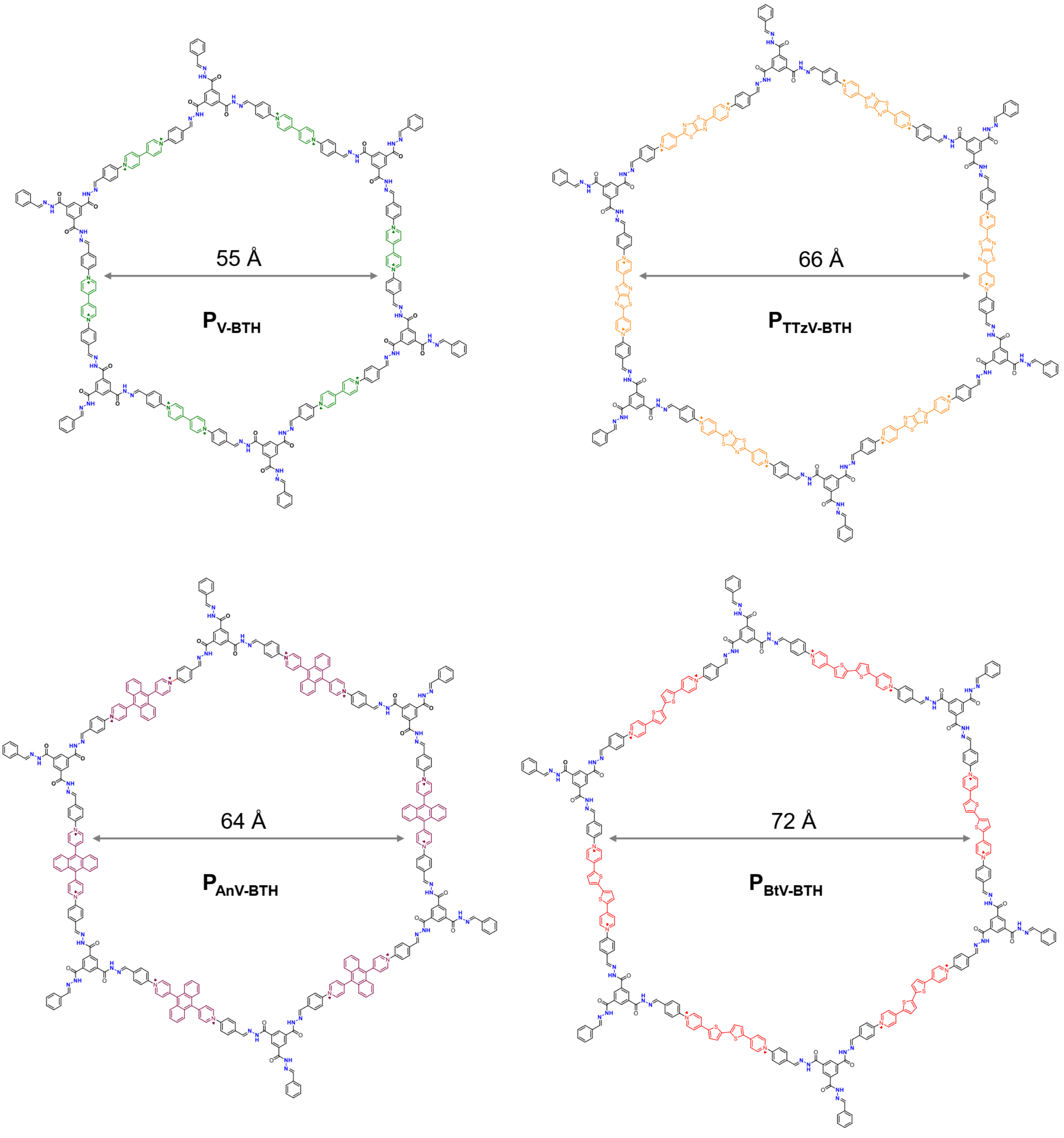

Figure S30. Proposed (idealized) pore structure of the four $\mathbf{P_{\pi V\text{-}BTH}}$ networks and the calculated pore sizes.

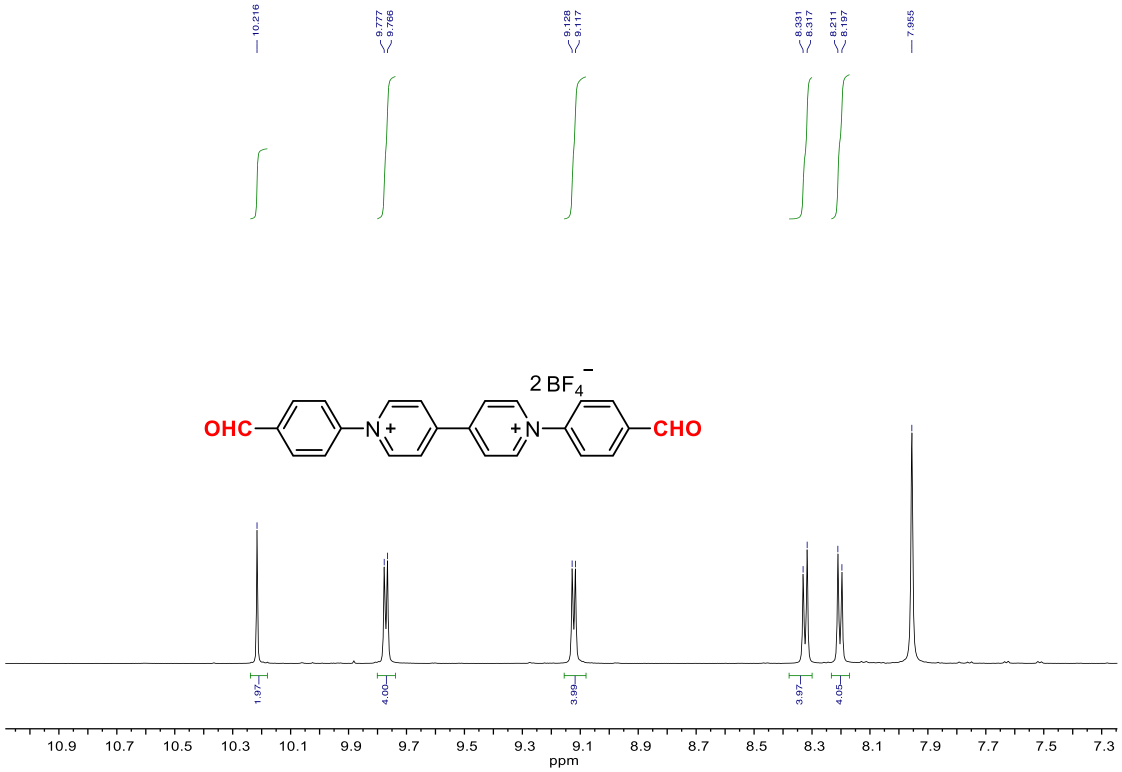

Figure S31. ^{1}H NMR spectrum of **V-CHO** in *d*6-DMSO.

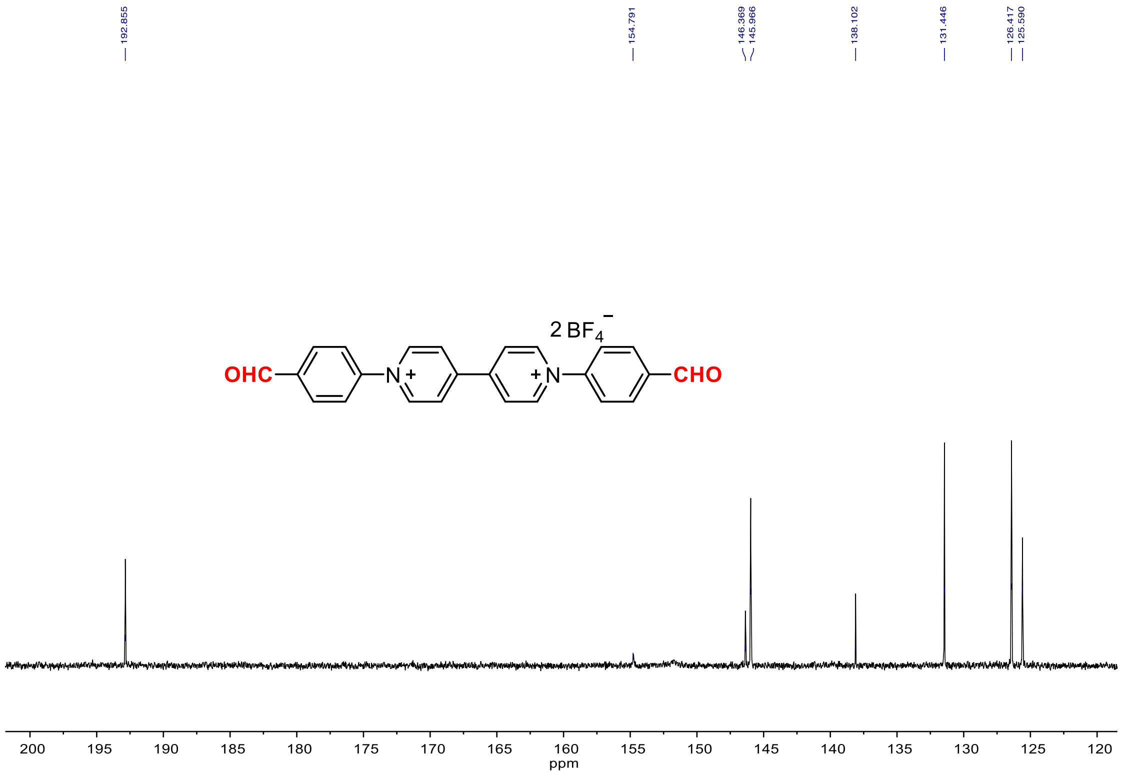

Figure S32. ^{13}C NMR spectrum of **V-CHO** in *d*6-DMSO.

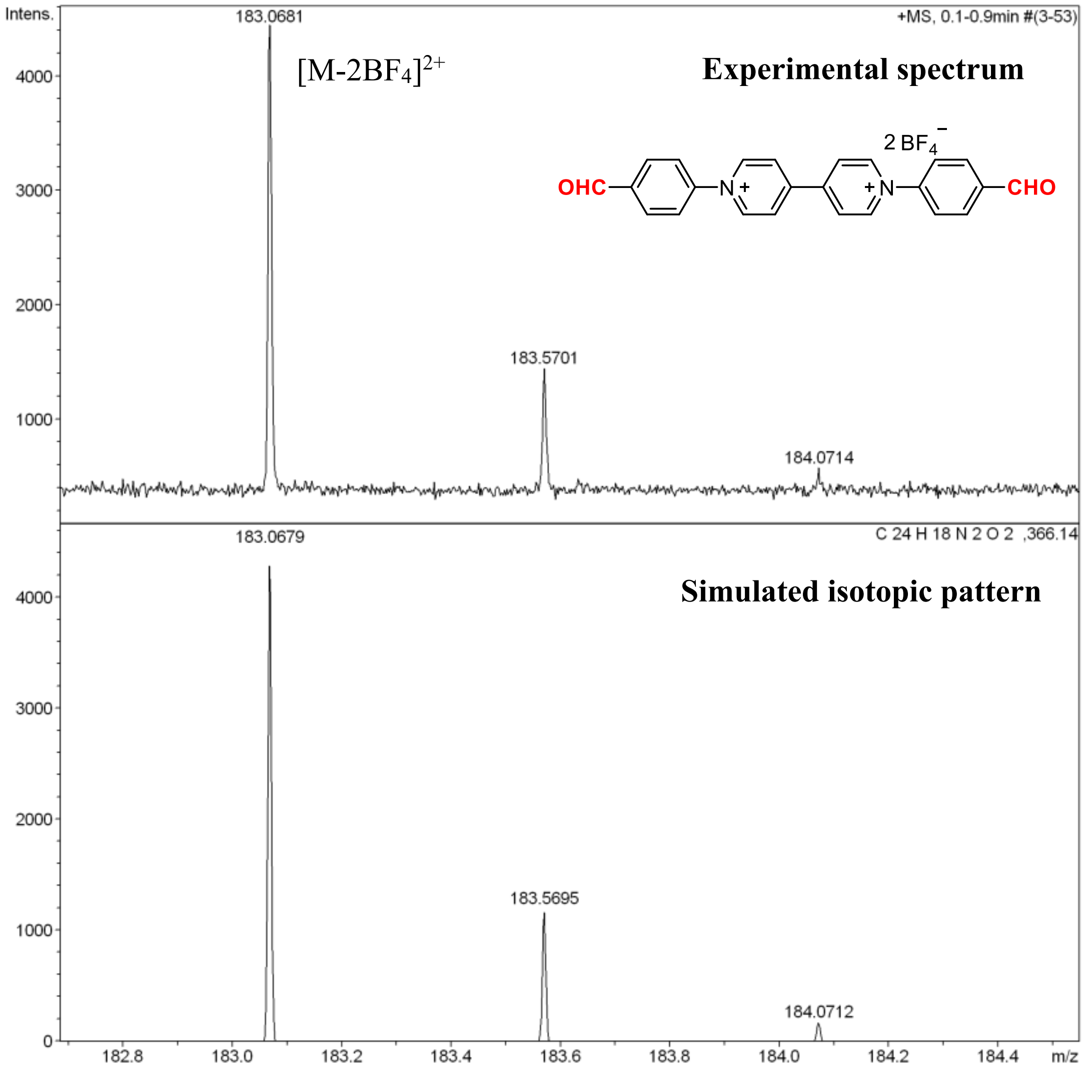

Figure S33. ESI-MS spectrum of **V-CHO**.

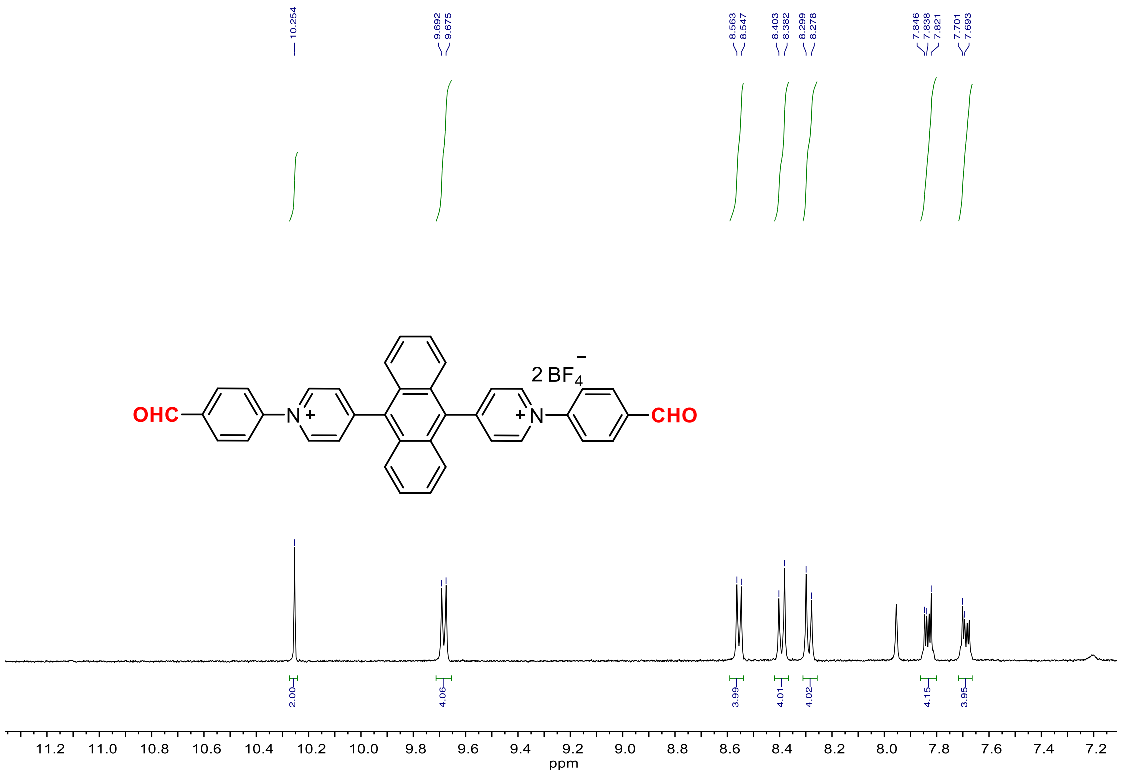

Figure S34. ^{1}H NMR spectrum of **AnV-CHO** in *d*6-DMSO.

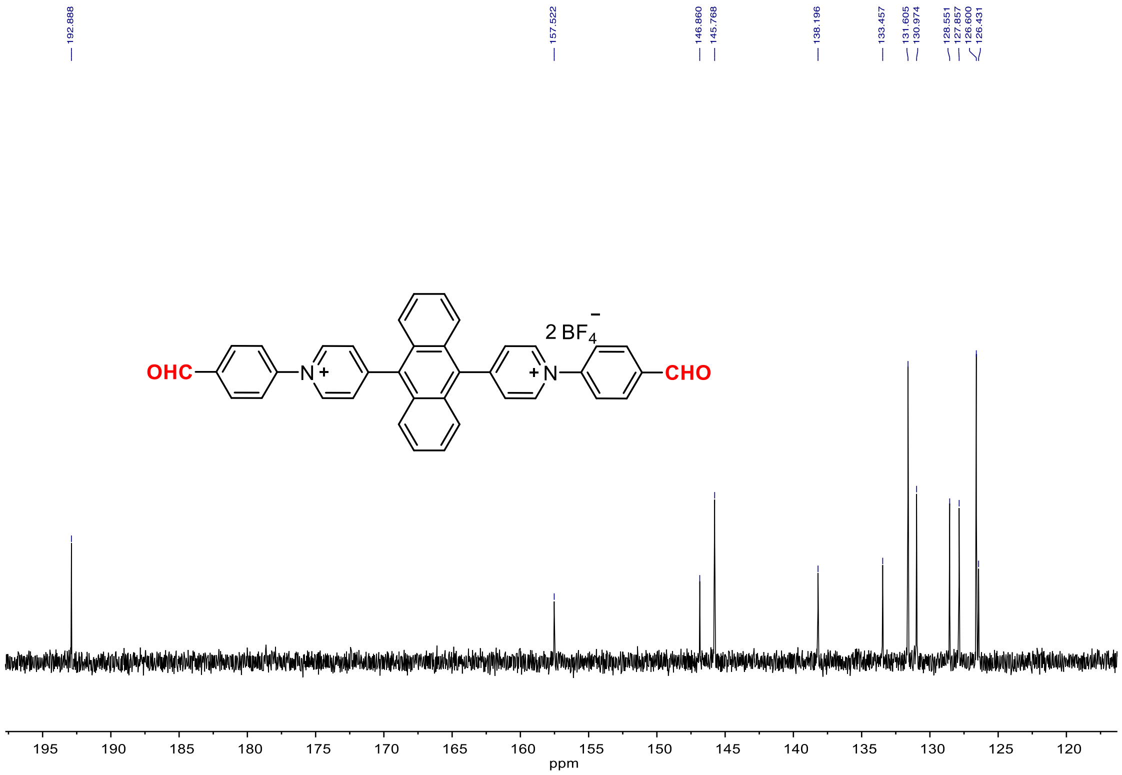

Figure S35. ^{13}C NMR spectrum of **AnV-CHO** in *d*6-DMSO.

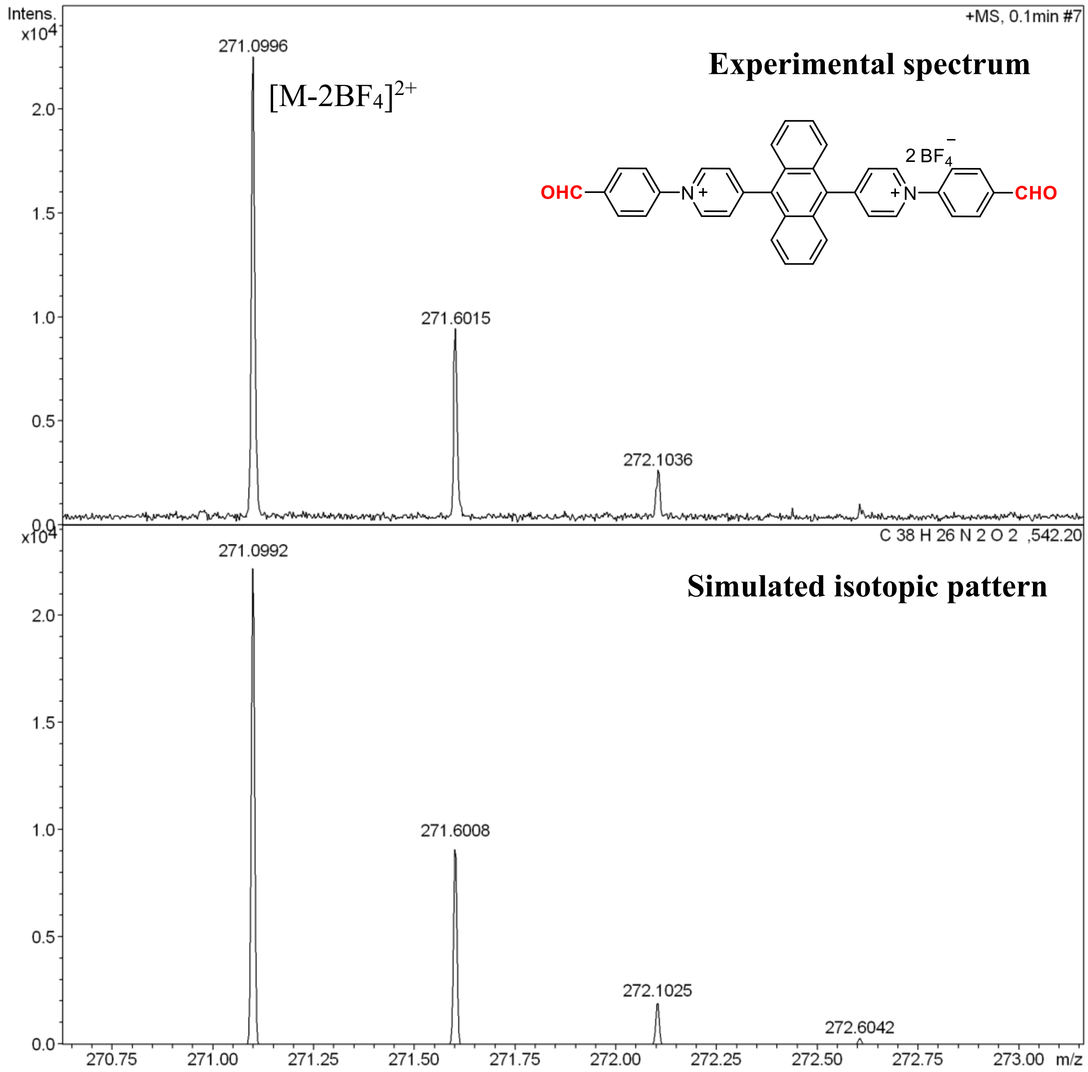

Figure S36. ESI-MS spectrum of **AnV-CHO.**

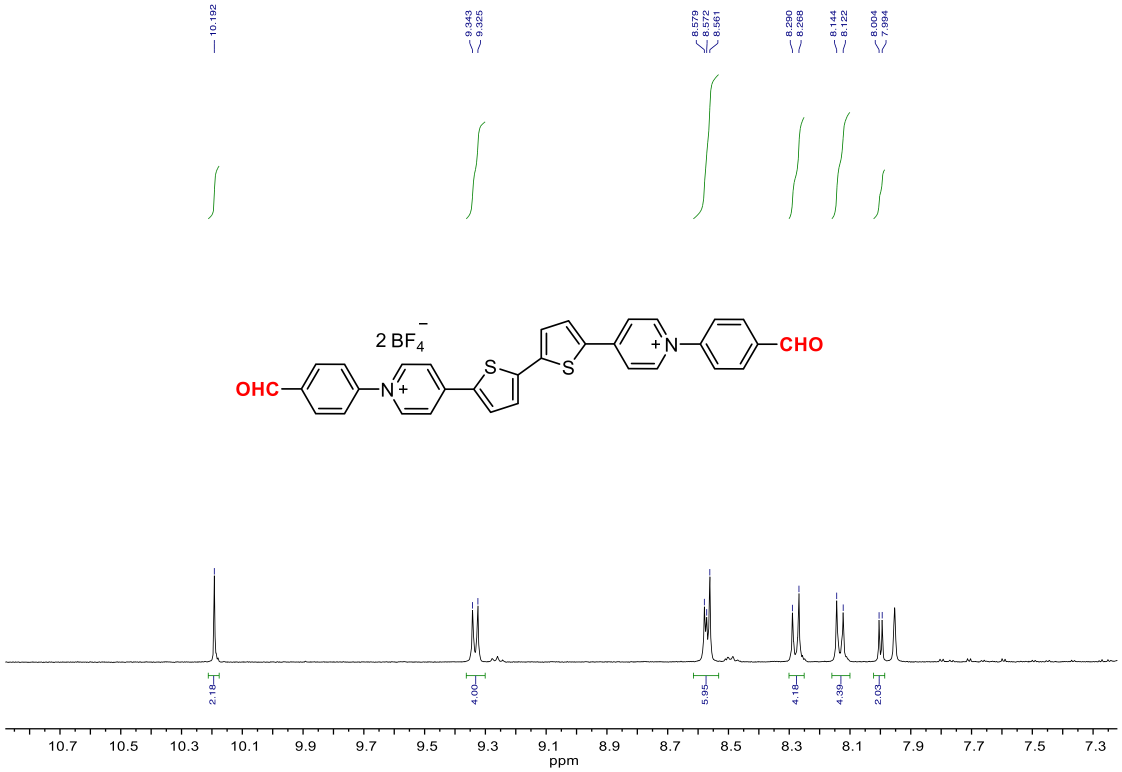

Figure S37. ^{1}H NMR spectrum of **BtV-CHO** in *d*6-DMSO.

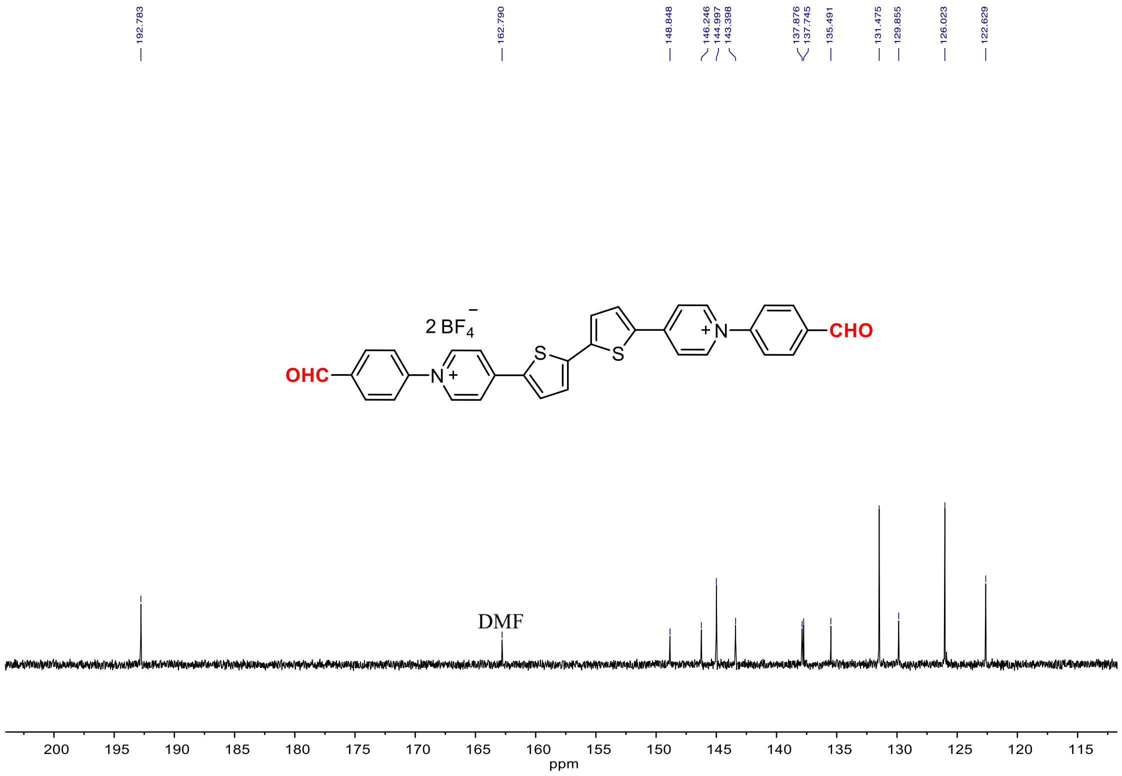

Figure S38. ^{13}C NMR spectrum of **BtV-CHO** in *d*6-DMSO.

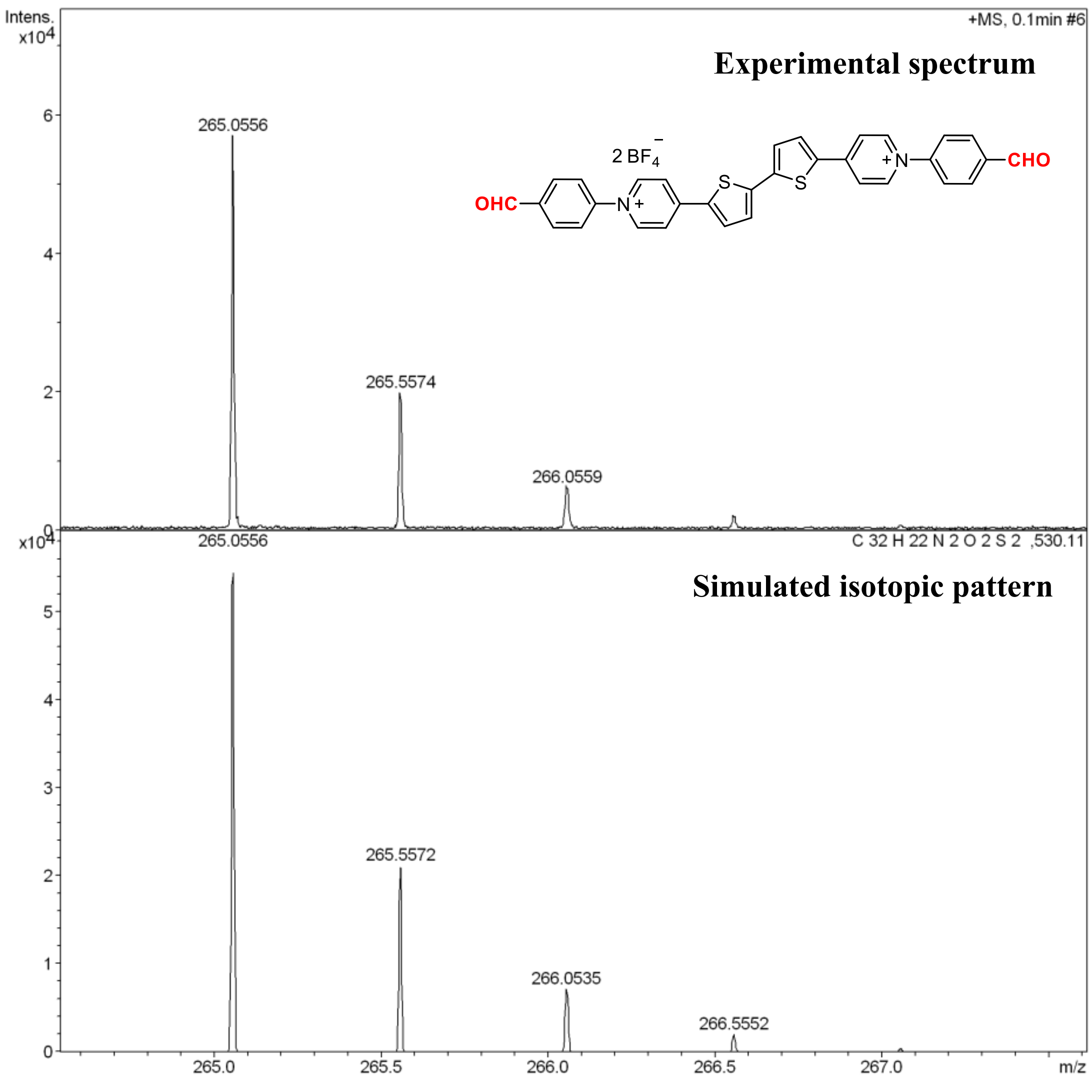

Figure S39. ESI-MS spectrum of **BtV-CHO.**

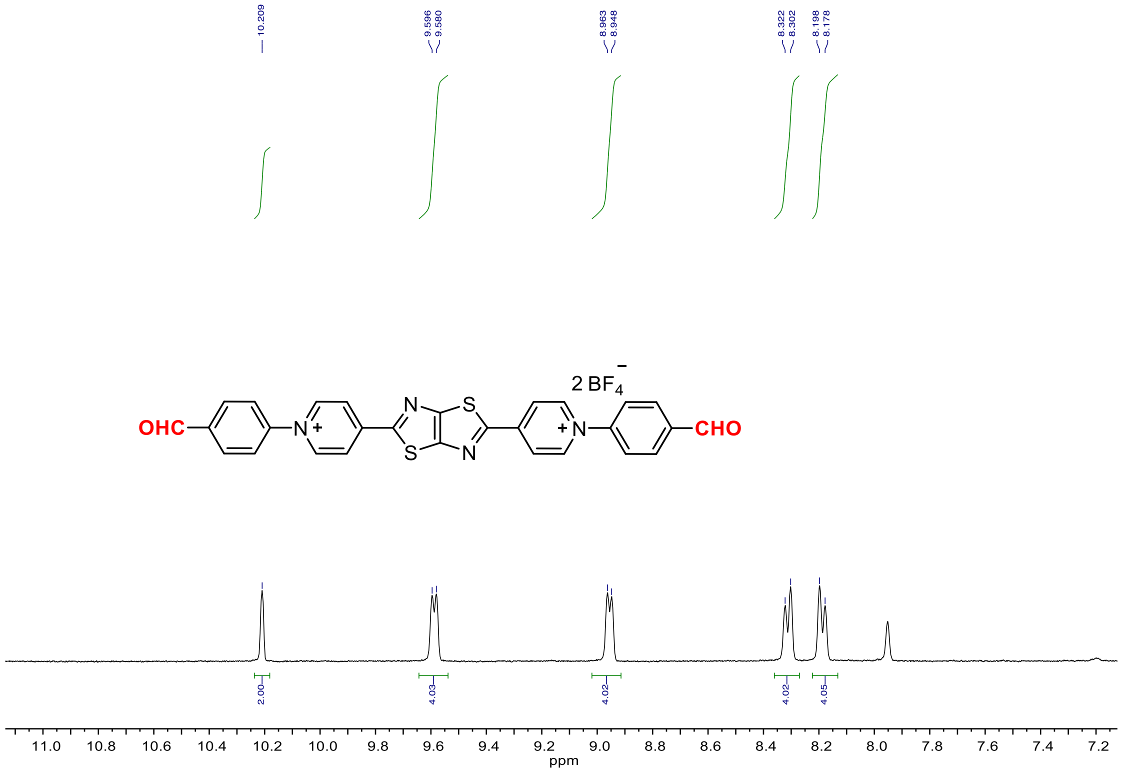

Figure S40. ^{1}H NMR spectrum of **TTzV-CHO** in *d*6-DMSO.

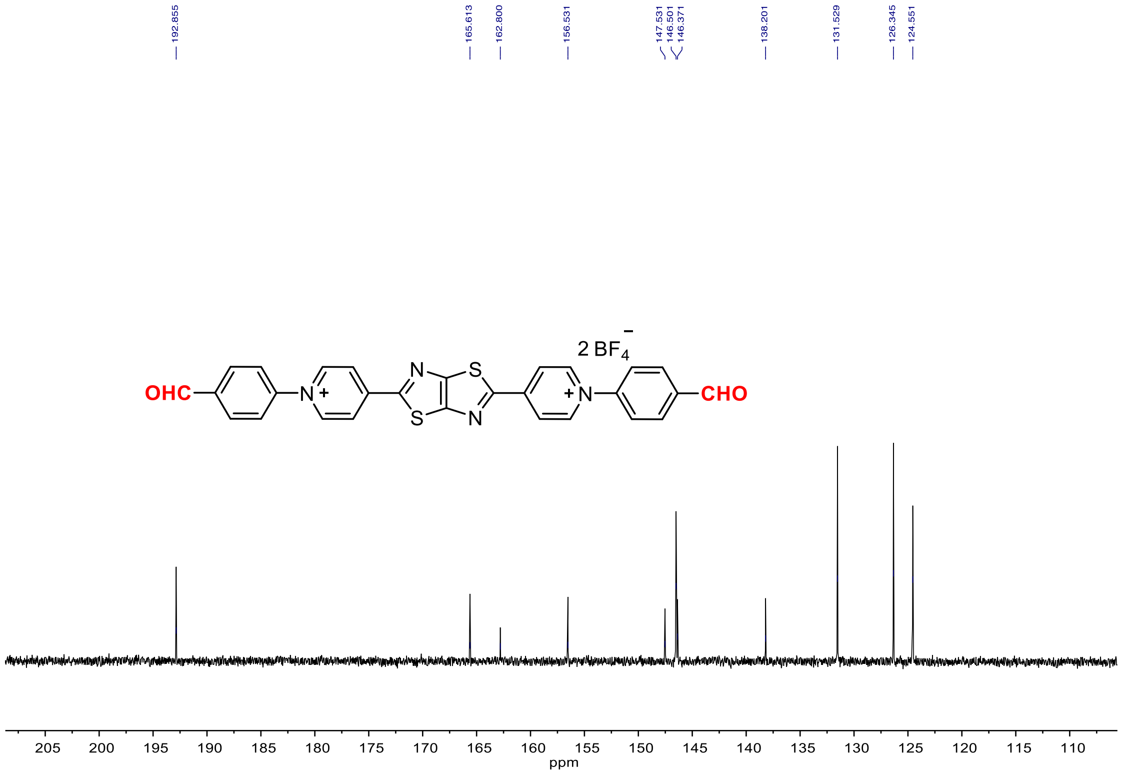

Figure S41. ^{13}C NMR spectrum of **TTzV-CHO** in *d*6-DMSO.

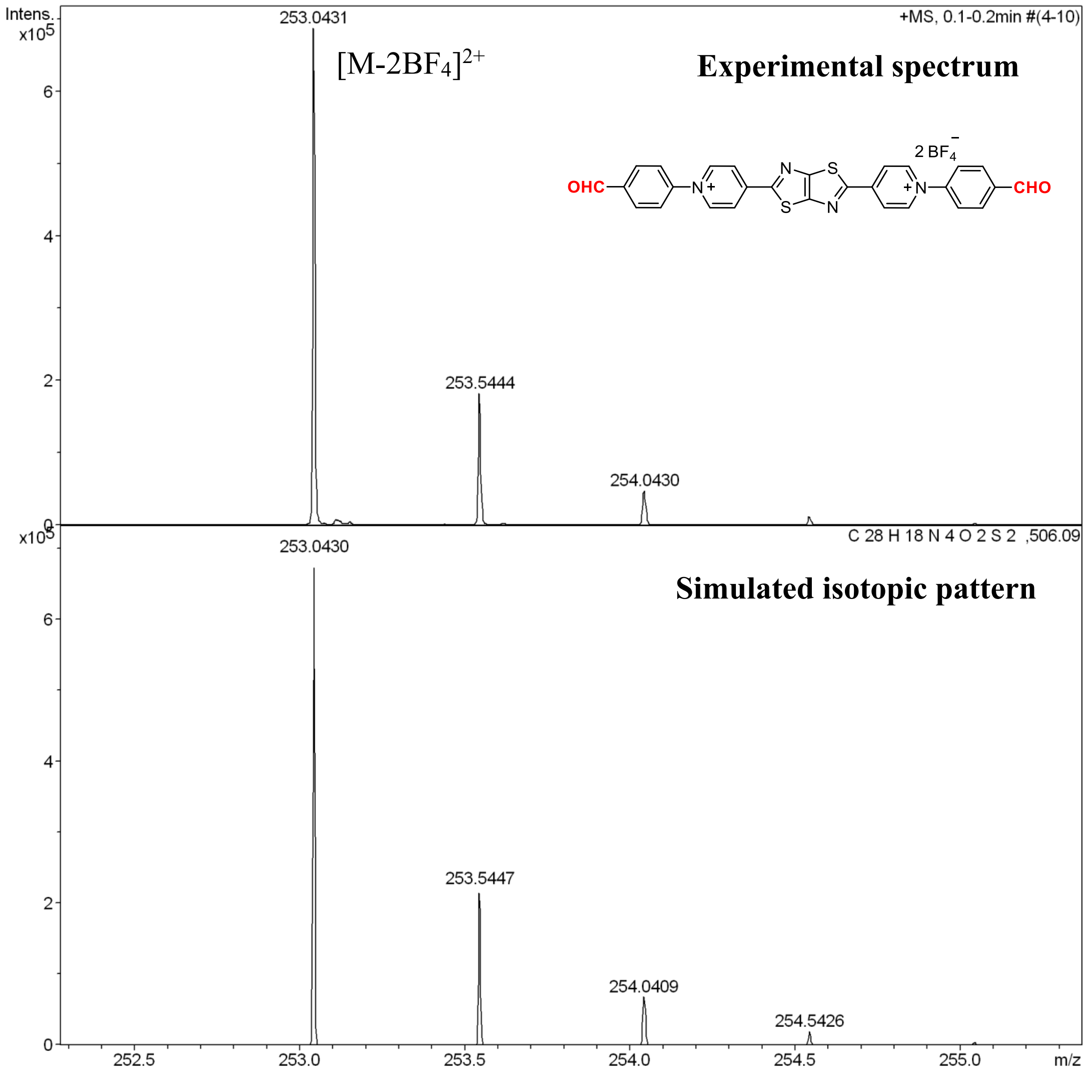

Figure S42. ESI-MS spectrum of **TTzV-CHO**.

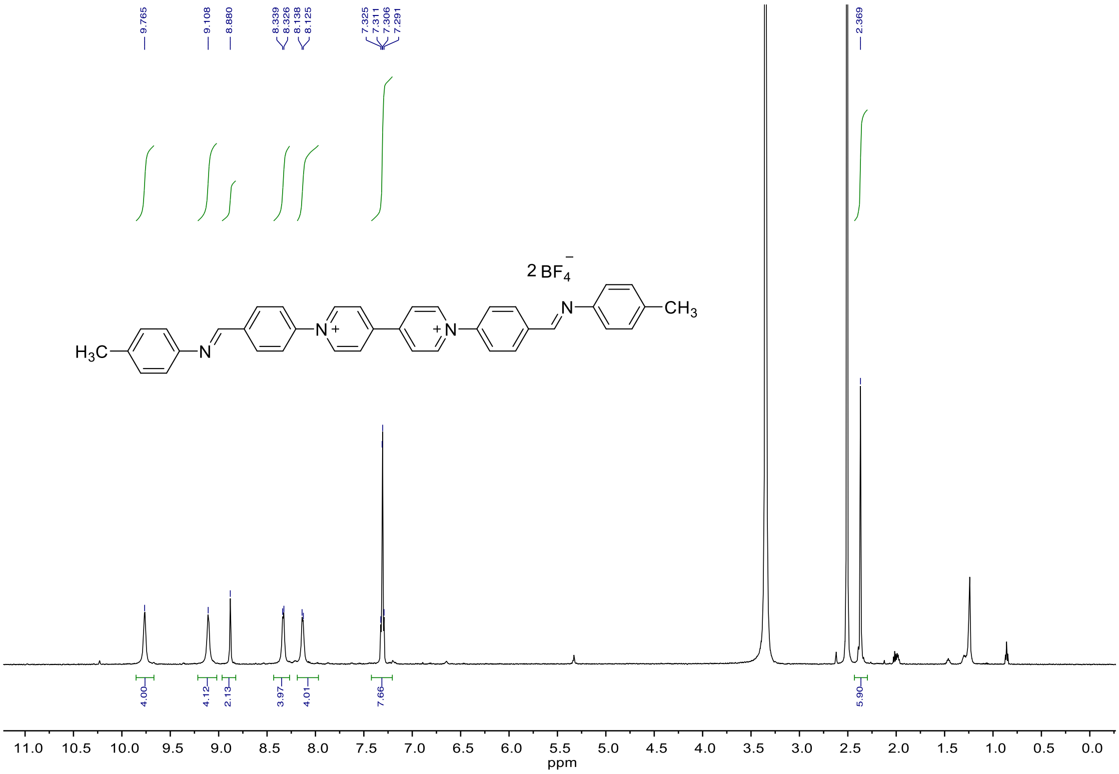

Figure S43. ^{1}H NMR spectrum of **V-CN** in *d*6-DMSO.

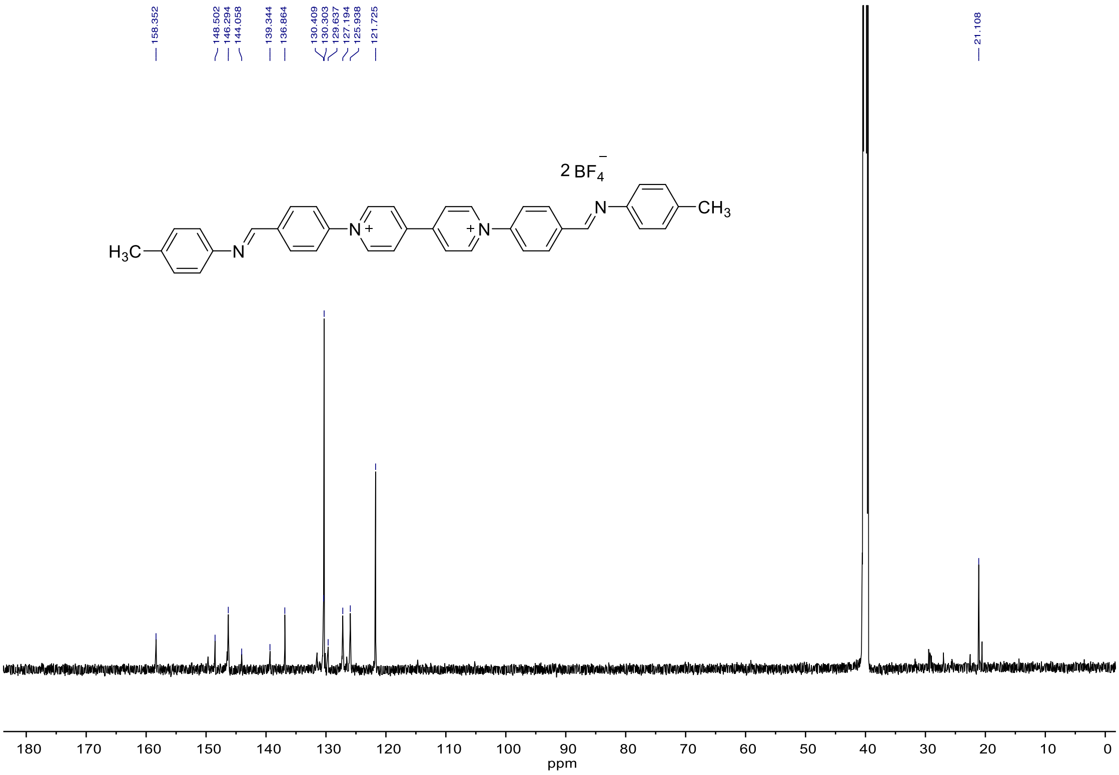

Figure S44. ^{13}C NMR spectrum of **V-CN** in *d*6-DMSO.

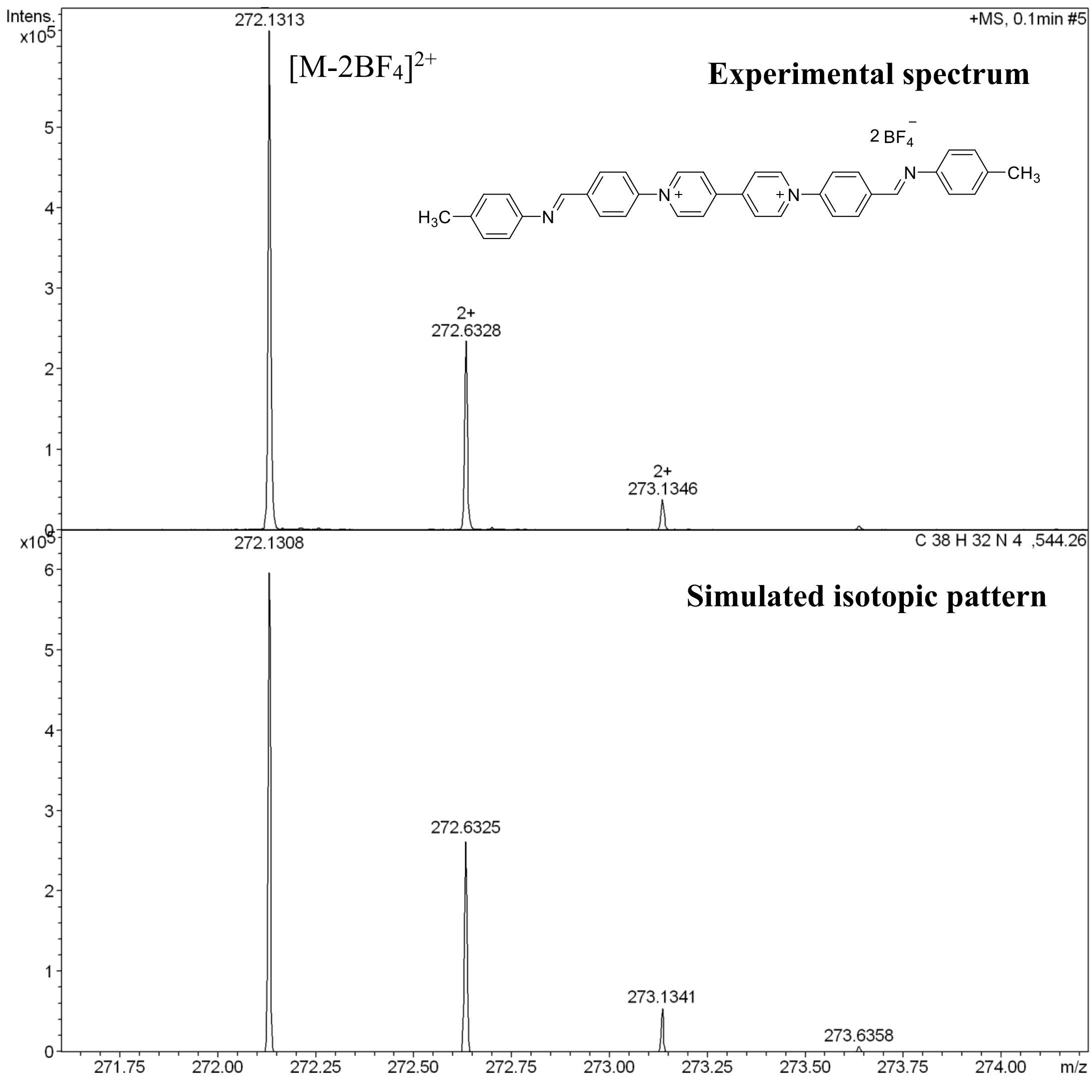

Figure S45. ESI-MS spectrum of **V-CN**.

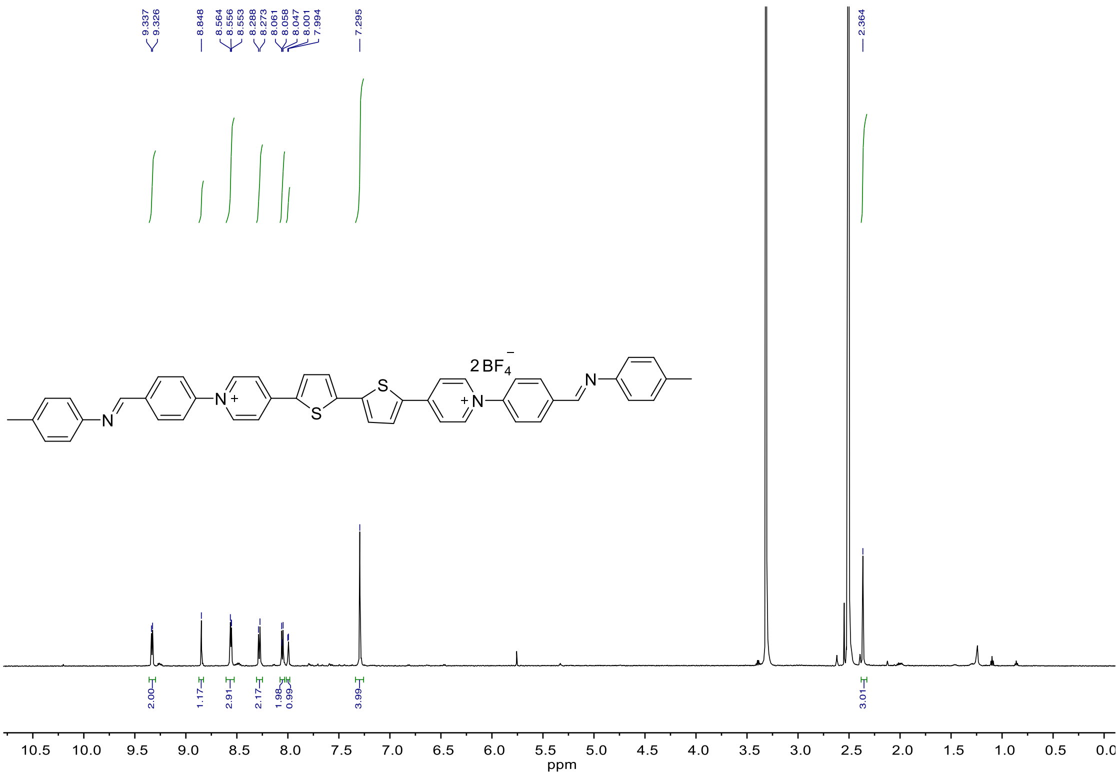

Figure S46. ^{1}H NMR spectrum of **BtV-CN** in *d*6-DMSO.

Table S2. Comparison of performance of the state-of-art viologen based EC materials.

EC materials	V_c / V_b (V)	t_c / t_b (s)	ΔT% (at nm)	stability (cycles /ΔT% drop)	CE[f] (cm^2 C^{-1})	ref
**P_{V-BTH}**	-0.4/+0.8	1.4/1.7	70.0 (600)	1000 /14%	73	This work
**$P_{TTzV-BTH}$**	-0.7/+0.6	1.0/0.8	89.8 (800)	1000 /6%	268	This work
**$P_{BtV-BTH}$**	-1.0/+0.4	0.8/1.3	95.6 (800)	1000 /7%	235	This work
V2DP	-2.0/+1.0	2.8/1.2	25(557)	840 /60%	989	S7
aPI	-2.0/+1.0	4.3/2.1	31(690)	nd	558	S7
POV	0/+0.8	1.2/2.4	38(720)	200/8%	122.6	S8
TEG-[C4C3bpy]TFSI)	0/+1.8	72/nd	10.1(630)	1000/40%	36.1	S9
TEG-[C6C3bpy]TFSI)	0/+2.0	14/nd	21.6(605)	1000/3%	76.8	S9
PEG-[C4C3bpy]TFSI)	0/+2.4	59/nd	18.3(616)	1000/55%	12.3	S9
PSVT	0/+0.8	2.8/2.2	31(700)	100/3.5%	288	S10
PSPT	0/+0.6	4.6/3.7	53(700)	100/0.7%	224	S10
TPA-V	-1.4/0	19/28.5	30(494)	500/1%	398	S11
PCP4	0/1	3.8/3.6	56(660)	1000/58%	nd	S12
PCP6	0/1	5.5/5.8	32(660)	1000/28%	nd	S12
BV/Fc/PVA-10	0/1.3V	6.1/7.7	66(605)	5000/20%	62.61	S13
BBV/Fc/PVA-10	0/1.3V	7.4/3.9	71(610)	5000/5%	102.62	S13
VBV	0/0.5V	3.4/3.1	63.7(570)	1000/3%	nd	S14
[OTxHV-POSS][PF_6]$_{16}$	-2.0/0	9.0/3.0	46.6(508)	3000/3%	366.6	S15
[OPhHV-POSS][PF_6]$_{16}$	-2.0/0	10.0/8.0	24.0(578)	3000/3%	137.3	S15

[OHV-POSS][PF_6]$_{16}$	-2.0/0	8.6/8.8	67.2(532)	3000/10%	307.8	S15
[OHHV-POSS][Br]8[Cl]8	-2.0/0	21.1/18.0	70(604)	300/10%	260.61	S16
PIL	0/4	55/nd	66.9(700)	nd	73.2	S17
poly(NIPAM)n-HV	0/2.7	0.5/3.7	85(555)	nd	85.4	S18

nd: Not determined.

References

S1. Liu, L.; Qu, W.-D.; Dong, K.-X.; Qi, Y.; Gong, W.-T.; Ning, G.-L.; Cui, J.-N. An anthracene extended viologen-incorporated ionic porous organic polymer for efficient aerobic photocatalysis and antibacterial activity. *Chem. Commun.* **2021**, *57*, 3339-3342.

S2. Demay-Drouhard, P.; Baumgartner, T. Highly Luminescent 4-Pyridyl-Extended Dithieno[3,2-*b*:2′,3′-*d*]phospholes. *J. Org. Chem.* **2020**, *85*, 14627-14633.

S3. Luo, J.; Hu, B.; Debruler, C.; Liu, T. L. A π-Conjugation Extended Viologen as a Two-Electron Storage Anolyte for Total Organic Aqueous Redox Flow Batteries. *Angew. Chem. Int. Ed.* **2018**, *57*, 231-235.

S4. Iinuma, M.; Moriyama, K.; Togo, H. Simple and Practical Method for Preparation of [(Diacetoxy)iodo]arenes with Iodoarenes and *m*-Chloroperoxybenzoic Acid. *Synlett* **2012**, *23*, 2663.

S5. Sundalam, S. K.; Stuart, D. R. Base Mediated Synthesis of Alkyl-aryl Ethers from the Reaction of Aliphatic Alcohols and Unsymmetric Diaryliodonium Salts. *J. Org. Chem.* **2015**, *80*, 6456-6466.

S6. Frisch, M. J.; Trucks, G. W.; Schlegel, H. B.; Scuseria, G. E.; Robb, M. A.; Cheeseman, J. R.; Scalmani, G.; Barone, V.; Mennucci, B.; Petersson, G. A.; Nakatsuji, H.; Caricato, M.; Li, X.; Hratchian, H. P.; Izmaylov, A. F.; Bloino, J.; Zheng, G.; Sonnenberg, J. L.; Hada, M.; Ehara, M.; Toyota, K.; Fukuda, R.; Hasegawa, J.; Ishida, M.; Nakajima, T.; Honda, Y.; Kitao, O.; Nakai, H.; Vreven, T.; Montgomery, J. A., Jr.; Peralta, J. E.; Ogliaro, F.; Bearpark, M.; Heyd, J. J.; Brothers, E.; Kudin, K. N.; Staroverov, V. N.; Kobayashi, R.; Normand, J.; Raghavachari, K.; Rendell, A.; Burant, J. C.; Iyengar, S. S.; Tomasi, J.; Cossi, M.; Rega, N.; Millam, J. M.; Klene, M.; Knox, J. E.; Cross, J. B.; Bakken, V.; Adamo, C.; Jaramillo, J.; Gomperts, R.; Stratmann, R. E.; Yazyev, O.; Austin, A. J.; Cammi, R.; Pomelli, C.; Ochterski, J. W.; Martin, R. L.; Morokuma, K.; Zakrzewski, V. G.; Voth, G. A.; Salvador, P.; Dannenberg, J. J.; Dapprich, S.; Daniels, A. D.; Farkas, Ö.; Foresman, J. B.; Ortiz, J. V.; Cioslowski, J.; Fox, D. J. Gaussian09, Revision A.02, Gaussian, Inc.: Wallingford CT, 2009.

S7. Wang, Z.; Jia, X.; Zhang, P.; Liu, Y.; Qi, H.; Zhang, P.; Kaiser, U.; Reineke, S.; Dong, R.; Feng, X. Viologen-Immobilized 2D Polymer Film Enabling Highly Efficient Electrochromic Device for Solar-Powered Smart Window. *Adv. Mater.* **2022**, *34*, 2106073.

S8. Zhou, Y.; Liu, X.; Jia, X.; Chao, D. Dual-electrochromic polymer bearing oligoaniline and viologen pendants: Synthesis and properties. *Eur. Polym. J.* **2019**, *111*, 43-48.

S9. Puguan, J. M. C.; Kim, H. A switchable single-molecule electrochromic device derived from a viologen-tethered triazolium-based poly(ionic liquid). *J. Mater. Chem. A* **2019**, *7*, 21668-21673.

S10. Wang, Y.; Jia, X.; Berda, E. B.; Zhao, J.; Liu, X.; Chao, D. Design and synthesis of multicolor electrochromic polymers based on oligoaniline and viologen/phenothiazine groups. *Eur. Polym. J.* **2020**, *138*, 109979.

S11. Kim, K.-W.; Lee, J. K.; Tang, X.; Lee, Y.; Yeo, J.; Moon, H. C.; Lee, S. W.; Kim, S. H. Novel triphenylamine containing poly-viologen for voltage-tunable multi-color electrochromic device. *Dyes Pigm.* **2021**, *190*, 109321.

S12. Huang, J.-J.; Liang, H.-I.; Lin, H.-A.; Luo, S.-C. Corannulene Extended Viologen-Based Ambipolar Polymers for Transparent-to-Color Electrochromic Supercapacitors. *ACS Appl. Electron. Mater.* **2022**, *4*, 4996-5007.

S13. Tang, S.; Zheng, R.; Niu, J.; Wan, Z.; Jia, C.; Weng, X.; Deng, L. All-in-one electrochromic gel consist of benzylboronic acid viologen with superior long-term stability and self-healing property. *Sol. Energy Mater. Sol. Cells* **2023**, *257*, 112353.

S14. Kao, S.-Y.; Lu, H.-C.; Kung, C.-W.; Chen, H.-W.; Chang, T.-H.; Ho, K.-C. Thermally Cured Dual Functional Viologen-Based All-in-One Electrochromic Devices with Panchromatic Modulation. *ACS Appl. Mater. Interfaces* **2016**, *8*, 4175-4184.

S15. Pande, G. K.; Heo, J. S.; Choi, J. H.; Eom, Y. S.; Kim, J.; Park, S. K.; Park, J. S. RGB-to-black multicolor electrochromic devices enabled with viologen functionalized polyhedral oligomeric silsesquioxanes. *Chem. Eng. J.* **2021**, *420*, 130446.

S16. Sun, F.; Eom, J. H.; Kim, D. Y.; Pande, G. K.; Ju, H.; Chae, H. G.; Park, J. S. Large-Area Flexible Electrochromic Devices with High-Performance and Low-Power Consumption Enabled by Hydroxyhexyl Viologen-Substituted Polyhedral Oligomeric Silsesquioxane. *ACS Sustain. Chem. Eng.* **2023,** *11*, 5756-5763.

S17. Rathod, P. V.; Puguan, J. M. C.; Kim, H. Self-bleaching dual responsive poly(ionic liquid) with optical bistability toward climate-adaptable solar modulation. *Chem. Eng. J.* **2021**, *422*, 130065.

S18. Chavan, P. V.; Rathod, P. V.; Lee, J.; Kostjuk, S. V.; Kim, H. Active and passive modulation of solar light transmittance in a uniquely multifunctional dual-band single molecule for smart window applications. *J. Energy Chem.* **2024,** *88*, 293-305.
